# Mitotic activity: A systematic literature review of the assessment methodology and prognostic value in canine tumors

**DOI:** 10.1177/03009858241239565

**Published:** 2024-03-27

**Authors:** Christof A. Bertram, Taryn A. Donovan, Alexander Bartel

**Affiliations:** 1 University of Veterinary Medicine Vienna, Vienna, Austria; 2 Schwarzman Animal Medical Center, New York, NY; 3 Freie Universität Berlin, Berlin, Germany

**Keywords:** dog, mitosis, mitotic count, mitotic index, neoplasia, outcome, survival, prognosis

## Abstract

One of the most relevant prognostic indices for tumors is cellular proliferation, which is most commonly measured by the mitotic activity in routine tumor sections. The goal of this systematic review was to analyze the methods and prognostic relevance of histologically measuring mitotic activity that have been reported for canine tumors in the literature. A total of 137 articles that correlated the mitotic activity in canine tumors with patient outcome were identified through a systematic (PubMed and Scopus) and nonsystematic (Google Scholar) literature search and eligibility screening process. Mitotic activity methods encompassed the mitotic count (MC, number of mitotic figures per tumor area) in 126 studies, presumably the MC (method not specified) in 6 studies, and the mitotic index (MI, number of mitotic figures per number of tumor cells) in 5 studies. A particularly high risk of bias was identified based on the available details of the MC methods and statistical analyses, which often did not quantify the prognostic discriminative ability of the MC and only reported *P* values. A significant association of the MC with survival was found in 72 of 109 (66%) studies. However, survival was evaluated by at least 3 studies in only 7 tumor types/groups, of which a prognostic relevance is apparent for mast cell tumors of the skin, cutaneous melanoma, and soft tissue tumor of the skin and subcutis. None of the studies using the MI found a prognostic relevance. This review highlights the need for more studies with standardized methods and appropriate analysis of the discriminative ability to prove the prognostic value of the MC and MI in various tumor types. Future studies are needed to evaluate the influence of the performance of individual pathologists on the appropriateness of prognostic thresholds and investigate methods to improve interobserver reproducibility.

Dogs with malignant tumors exhibit a variable clinical course based on certain tumor and patient characteristics.^94^ One of the most relevant tumor characteristics regarding prognostication is cellular proliferation.^43,94^ While most measurement methods of tumor proliferation require immunohistochemistry (such as the Ki67 index) or special stains (such as the AgNOR score), the most practical approach is to measure mitotic activity in routine hematoxylin and eosin–stained tumor sections.^142^

The histological measurement methods for mitotic activity (quantification of mitotic figures, ie, cells in the M phase of cell division with histologically distinct features) vary widely in previous studies.^94^ Two broad categories can be distinguished: (1) the mitotic count (MC) represents the absolute number of mitotic figures per tumor area and (2) the mitotic index (MI) represents the proportion of mitotic figures among all tumor cells per tumor area.^95^ While there have been efforts, starting in 2016, to standardize the measurement method of the MC,^43,94,95^ many studies were published before those guidelines were available. An overview of the previously applied methods is needed to better understand current practice and to direct future recommendations.

A vast number of studies have evaluated mitotic activity (mostly the MC) as a prognostic test in several canine tumor types. While mitotic activity is generally considered to be associated with the biological behavior of tumors and outcome of tumor patients,^43,94^ there are currently no recommendations on which tumor types mitotic activity should be assessed routinely as a solitary prognostic test. Due to the methodological differences in prognostic studies and the intrinsic bias of observational studies, validation of research findings and summaries through systematic review (and ideally meta-analysis) are needed for each tumor type.^18,93^

The goal of this systematic review was to analyze the methods and prognostic relevance of histologically measuring mitotic activity in canine tumors that have been reported in the literature. We provide an overview of the literature, as well as recommendations for routine diagnostic practice and future research goals.

## Material and Methods

This systematic review was conducted similar to a previous systematic review on mitotic activity in feline tumors using the same literature search protocol (with modified search terms and a single literature reviewer), data extraction, and article evaluation (risk of bias) criteria.^15^

## Literature Search

**Figure 1.**
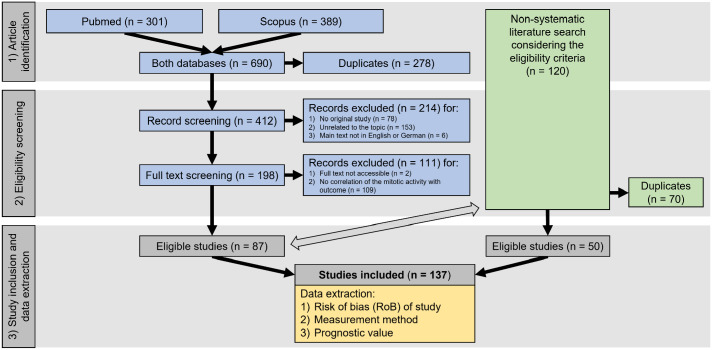
PRISMA flow diagram^110^ of the literature search divided into systematic (blue boxes) and non-systematic (green boxes) article identification and eligibility screening, followed by study inclusion with subsequent data extraction. The double-sided arrow indicates comparison of the identified articles for removal of duplicates.

References were identified by 1 author (CAB) through systematic (predetermined search terms) and nonsystematic (with numerous search terms) searches to ensure literature saturation (Fig. 1), consistent with the recommendations by the Preferred Reporting Items for Systematic Reviews and Meta-Analyses (PRISMA) statement.^110^ Eligibility screening was performed by a single author (CAB) using a 2-step procedure (Fig. 1).

Systematic literature identification was carried out in 2 databases, namely PubMed (1950 to present) and Scopus (1970 to present), on 30 April 2022 using the following predetermined search terms to build search strings on 2 topics for who (animal) and what (prognostic test): (dog OR dogs OR canine) AND (mitotic count OR mitotic index). Duplicates were removed using the literature management software Endnote X9.3.3 after sorting the articles alphabetically by their title. Subsequently, the 2-step eligibility screening was carried out in Rayyan,^109^ a Web app for collaborative systematic literature reviews, using the inclusion/exclusion criteria as provided in Table 1. Articles that had reported the prognostic value of the MC or MI (histologically determined) as a solitary prognostic test for potentially malignant tumor types in dogs were included. The artificial intelligence application in Rayyan was not used for this systematic review.

**Table 1.** Summary of the inclusion/exclusion criteria for the 2 eligibility screening steps applied to the identified references.

Screening Step	Decision Category		Inclusion Criteria	Exclusion Criteria
Title-abstract	1) Study design		Original study, peer-reviewed	Case reports, reviews
	2) Topic3) Language of main text	a) Species	Dog / Canine	Other species
		b) Tumor	Spontaneous tumors	Experimentally induced tumors
			Malignant tumors with potential for metastasis	Benign tumors
		c) Prognostic test	Mitotic count (MC), mitotic index (MI)	No mitotic activity measurement
		d) Examination method	Histology	Cytology
			English or German	Other language
Full text	1) Article accessibility		Article accessible	Article inaccessible
	2) Topic	a) Patient outcome	Correlation of the MC/MI with survival, tumor progression, metastasis, or recurrence	No correlation of the MC/MI with patient follow-up

A nonsystematic literature search in Google Scholar and the perusal of citing references (“cited by” search in Google Scholar) and cited references were conducted in 2022 for several weeks until 30 April 2022, resulting in a thorough evaluation of the available literature on prognostication of canine tumors. The nonsystematic literature search intended to find articles that were missed by the systematic search due to the lack of search terms being included in the title, abstract, or keywords of some articles. The search terms for Google Scholar were numerous and included “outcome,” “prognosis,” “survival,” and relevant tumor types such as “mast cell tumor,” “soft tissue sarcoma,” “melanoma,” and so on. The title and abstract of relevant articles (as sorted by Google Docs) were screened. The full text of articles of potential interest were downloaded and evaluated for the aforementioned eligibility criteria. Only articles that met these criteria were included. Finally, duplicates to the systematic literature search were excluded.

## Data Extraction and Analysis

Information regarding the publication (paper identification, year of publication, journal), tumor type evaluated, measurement methods of the MC or MI, and prognostic value of mitotic activity was extracted from each article in the same way as previously described.^15^ Statistical significance of the prognostic value was based on the reported *P* values with *P* ≤ .05 being indicative of significant results.

Risk of bias of each study was evaluated (low, moderate, and high) specifically for the information regarding the mitotic activity using a previously developed protocol^15^ (Supplemental Table S1) for objectivity and transparency. The overall risk of bias was based on 4 domains: (1) study population, (2) outcome assessment, (3) mitotic activity methods, and (4) data analysis. As detailed in Supplemental Table S1, domain 1 (study population) was mostly based on the sample size per outcome event with a minimum of 7 events for a moderate risk of bias and 15 events for a low risk of bias, as well as the presumed representativeness of the study population and the availability of descriptions of the patient and tumor characteristics. The thresholds for the sample size were modified from the recommendations for multivariable statistical models (at least 10 cases per event for each variable),^158^ considering that this systematic review evaluated mitotic activity as a solitary test and accounting for the small sample size available for studies on rare tumor types. Domain 2 (outcome assessment) evaluated the methods of obtaining patient outcome information (type of outcome metrics, follow-up method and period, and confirmation of events), as well as the potential bias resulting from treatment regimens of the patients. Domain 3 (mitotic activity method) was based on completeness of the methods description and the assumed consistency of the applied measurement methods for evaluation of the study cases. Domain 4 (data analysis) focused on the use of appropriate statistical analysis to measure prognostic accuracy of the MC and MI.^18^

## Results

### Study Selection

The article identification and eligibility screening process are summarized in Fig. 1. Through the systematic literature search, 87 eligible articles out of 412 unique references were identified. Fifty additional articles were found during nonsystematic literature search, adding up to a total of 137 articles evaluated in this systematic review.^1–3,5,7–11,17,19–21,23–29,31–37,39–42,44–49,52–65,69–75,78–84,86–92,96–108,111–123,125–141,143–151,153–157,159–166^

### Study Characterization

All of the included articles were written in English. One-third (42/137, 31%) of the publications were published in journals focused on veterinary pathology (*Veterinary Pathology*, *Journal of Comparative Pathology*, and *Journal of Veterinary Diagnostic Investigation*).

**Figure 2.**
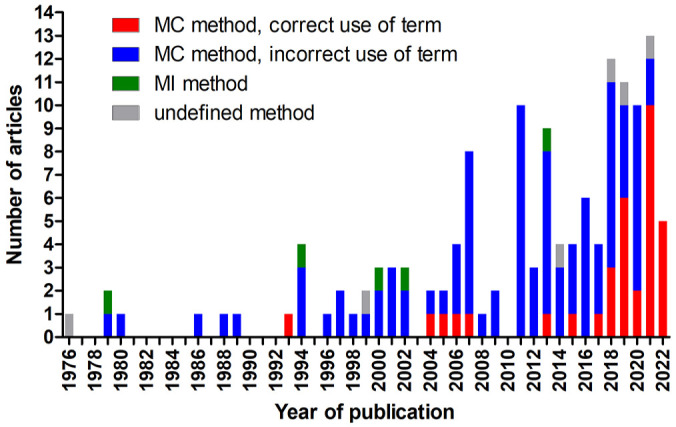
Stacked bar chart of the number of publications included in this systematic review per year of publication. The year 2022 includes publications until 30 April. MC, mitotic count; MI, mitotic index.

Based on the described methods, 126 articles evaluated the MC (number of mitotic figures per tumor area),^2,3,5,7–11,17,19–21,23–29,31–37,39–42,44–49,52–65,69,70,72–75,78–84,86–92,96,99–108,114–123,125–127,129–132,134–137,139–141,143–151,153–157,159–163,165,166^ 5 evaluated the MI (proportion of mitotic figures per number of tumor cells),^98,113,128,138,164^ and 6 did not specify the mitotic activity method in their paper.^1,71,97,111,112,133^ We assume that the articles without method specification performed the MC based on the mitotic activity values reported in the “Results” section,^71,111^ the prognostic threshold used to classify cases,^112,133^ or because the mitotic activity was likely determined as part of a grading system, which uses MC (based on the original method descriptions).^1,97^ For 3 of those 6 studies, confirmation of the MC method was obtained through personal communications with the authors.^1,111,112^ Thus, these 132 articles (96.4%) were used for analysis of the MC. The number of articles published per year included in this review increased over time with more than 10 articles published per year between 2018 and 2021 (Fig. 2).

While the 5 references that determined the MI always used the preferred term “mitotic index,” the other 132 articles employing the MC used various and sometimes multiple terms, including mitotic index (*N* = 83), mitotic count (*N* = 40), mitotic rate (*N* = 13), number of mitoses (*N* = 3), number of mitotic figures (*N* = 1), number of mitotic cells (*N* = 1), mitotic figures (*N* = 1), and mitoses (*N* = 1). Since publishing recommendations on the terminology for MC in 2016,^95^ the frequency of the use of correct terminology has improved. While only 12 of the 82 articles (15%) published before 2017 used the correct term, 28 of the 55 articles (51%) after 2016 used the recommended terminology (Fig. 2). Usage of the correct term after 2016 was even higher when the journal had a veterinary pathology focus (12/14, 86%) compared with other journals (16/41, 39%). Similarly, articles published in the journal focused on veterinary oncology (*Veterinary and Comparative Oncology*) reported the correct term for the MC more often after 2016 as compared with before 2017 (9/18 vs 0/10 studies, respectively).

## Mitotic Count

The 132 studies using MC evaluated numerous tumor types, while some studies included several tumor types or tumor locations or lacked relevant information on these tumor specifications (Table 2).

**Table 2.** Number of articles evaluating the mitotic count per tumor type/group and tumor specifications based on tumor types or locations.

Tumor Type/Group	Number of References	Tumor Specifications of the Articles
Mast cell tumors	29 (22%)	Skin (N = 24, cutaneous and subcutaneous), skin and mucocutaneous (N = 2), intramuscular (N = 1), oral mucosa (N = 1), unspecified location (N = 1)
Sarcomas, non-osteogenic	21 (16%)	Skin (N = 13), gastrointestinal (N = 4), visceral (N = 1), smooth muscle tumor (N = 1), appendicular, axial skeleton soft tissue and visceral (N = 1), unspecified location (N = 1)
Melanocytic tumors	20 (15%)	Oral (N = 9), cutaneous (N = 4), oral and cutaneous (N = 5), (intra)ocular (N = 2)
Mammary tumors	14 (11%)	Malignant tumors (N = 8), any (N = 4), carcinoma (N = 1), neuroendocrine carcinoma (N = 1)
Osteosarcoma	9 (7%)	Appendicular (N = 5), mandibular (N = 1), surface (N = 1), any (N = 2)
Lymphoma	8 (6%)	Multicentric (N = 2), diffuse large B-cell (N = 1), diffuse small B-cell (N = 1), Burkitt-like (N = 1), indolent (N = 1), small intestinal (N = 1), any (1)
Hemangiosarcoma	7 (5%)	Splenic (N = 2), cutaneous (N = 1), subcutaneous and intramuscular (N = 1), other than skin (N = 1), non-visceral (N = 1), falciform fat (N = 1)
Apocrine gland anal sac adenocarcinoma	5 (4%)	–
Splenic tumors	4 (3%)	Mesenchymal/stromal sarcoma (N = 2), fibrohistocytic nodules (N = 2)
Pulmonary tumors	3 (2%)	–
Renal cell carcinoma	2	–
Insulinoma	2	–
Squamous cell carcinoma	2	Skin (N = 2)
Salivary gland tumors	1	–
Glial tumors	1	–
Synovial sarcoma	1	–
Thymic tumors	1	–
Pheochromocytoma	1	–
Esophageal sarcoma	1	–

### Risk of Bias

The risk of bias of each of the 4 domains and the overall risk of bias is summarized in Table 3 and listed for each reference in Supplemental Table S2. Most studies (70/132, 53%) had a high overall risk of bias based on a high risk in at least 1 of the 4 domains. One study included some feline cases in the study population,^132^ which poses a high risk of bias as equality in the extent of the association of the MC with outcome between different species should not be expected. Data analysis (domain 4) was often restricted to a statistical test of significance, and in many studies, nonsignificant results were reported as “*P* > .05” or as “not significant,” while the actual *P* values were not provided (Supplemental Tables S5–S7).

**Table 3.** Summary of the risk of bias evaluation for 132 studies on the mitotic count (MC) based on 4 risk of bias domains (D1—D4).

Risk of Bias Category	Number and percent of articles				
	D1: Study Population	D2: Outcome Assessment	D3: MC Methods	D4: Data Analysis	Overall (D1—D4)
Low 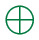	28 (21%)	17 (13%)	19 (14%)	6 (5%)	4 (3%)
Moderate 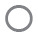	68 (52%)	92 (70%)	20 (15%)	61 (46%)	58 (44%)
High 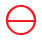	36 (27%)	23 (17%)	93 (71%)	65 (49%)	70 (53%)

The overall risk of bias was based on the 4 domains. The number and percentages of the 3 risk of bias categories add up to 132 articles and 100%, respectively, for each column (ie, for each risk of bias domain).

### Methods

The MCs were taken from pathology records in 19 of the 132 studies (14%), partially taken from pathology reports and newly determined in 2 of the 132 studies (2%), and likely newly determined by the same pathologist(s) for the study following the study protocol in the remaining 111 studies (84%). Multiple pathologists assessed all study cases in 20 of the 111 studies, most of which aggregated the multirater evaluations using various methods, including averaging and consensus. One study employed semi-quantitative scoring for assessing mitotic density,^92^ while the rest enumerated mitotic figures. Special staining methods were used in 1 study (toluidine blue).^116^ Seven of the 20 studies (35%) on melanoma specified that they used bleached slides (for all cases or for heavily pigmented cases), and 2 studies (10%) assigned an MC of 0 when nuclei were obscured by pigmentation. One study evaluated the MC in digital whole-slide images (20× and 40× scan magnification) and glass slides,^159^ while the remaining studies are presumed to have used light microscopy. The use of automated image analysis was not reported in any study.

**Figure 3.**
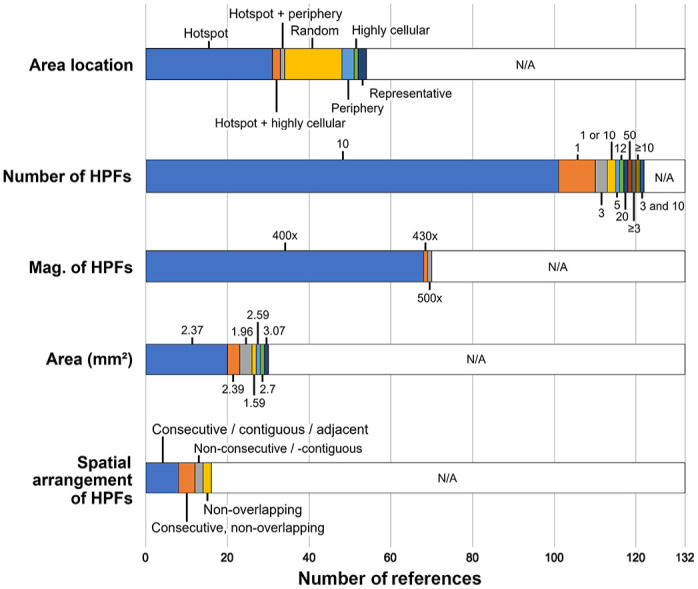
Stacked bar chart for the key methodical aspects of the mitotic count applied in the 132 studies. HPFs, high-power fields; Mag., magnification; N/A, not available.

A summary of the key methodical aspects of the MC applied in the 132 studies is depicted in Fig. 3 and detailed for each study in Supplemental Table S3. Generally, the proportion of studies that reported the details on the MC methods increased for studies published after 2016 and in journals with a focus on pathology (see summary of Supplemental Table S3).

### Prognostic Value

The outcome metrics evaluated in the 132 studies were overall or tumor-specific survival (*N* = 113, 86%), disease progression (occurrence of metastasis or local recurrence; *N* = 40, 30%), metastasis (*N* = 26, 20%), recurrence (*N* = 22, 17%, particularly for soft tissue tumors), and recurrence of hypoglycemia in insulinoma (*N* = 2, 2%). The number of complete cases varied between 6 and 384 (median: 50 cases; mean: 64 cases). The prognostic relevance of the MC determined by different pathologists was only ascertained in 1 study,^130^ whereas the other studies used MC values by 1 study pathologist or a consensus of pathologists.

**Figure 4.**
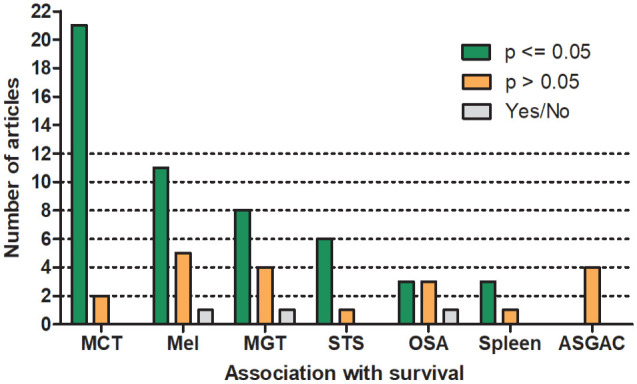
Number of studies that did or did not reach prognostic significance regarding the association of the mitotic count with survival for tumor types with more than 3 studies. Yes/No describes studies with significant association in only a subset of cases or pathologists. MCT, mast cell tumor of the skin (cutaneous and subcutaneous); Mel, cutaneous and oral melanoma; MGT, mammary gland tumors; STS, soft tissue sarcoma of the skin; OSA, osteosarcoma; Spleen, splenic stromal sarcoma and fibrohistiocytic nodules; ASGAC, anal sac gland adenocarcinoma.

For all tumor types/groups combined, a prognostic value (mostly determined using statistical significance) of mitotic activity was found in 55% to 63% of the studies regarding the different outcome metrics (Table 4 and Supplemental Tables S4–S7). The association of the MC with survival for the tumor types/groups with 3 or more studies is summarized in Fig. 4. However, the discriminant ability of the prognostic test could not be properly evaluated for many studies due to the lack of appropriate statistical analysis.

**Table 4.** Summary of the prognostic significance (mostly comprising the *P* value approach) of 132 studies on the mitotic count regarding survival, disease progression (metastasis or recurrence), metastasis, and recurrence.

Prognostic Significance	Number of Articles				
	Survival	Disease Progression	Metastasis	Tumor Recurrence	Recurrence of Clinical Signs
Yes	69 (63%)	21 (55%)	14 (58%)	11 (55%)	1 (100%)
Yes/No	3 (3%)	0	1 (4%)	0	0
No	37 (34%)	17 (45%)	9 (38%)	9 (45%)	0
ND	4	2	2	2	1
N/A	19	92	106	110	130

Yes/No, prognostic significance was found for only a subset of the cases or pathologists; ND, interpretation of the prognostic significance is not provided (individual patient data available); N/A, outcome metric not available.

**Figure 5.**
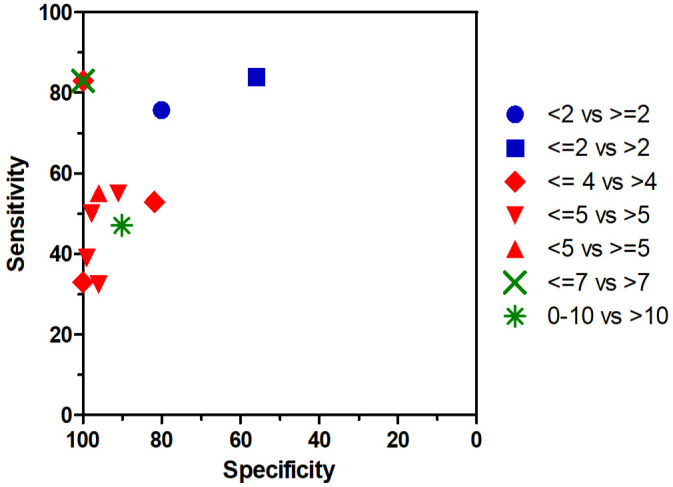
Summary of the sensitivity and specificity for survival in canine mast cell tumors of the skin based on different cut-off ranges. The data is reported in 9 studies (3 with 2 cut-offs).^10,11,20,53,57,62,149,155,157^

Survival was evaluated in 23 studies on mast cell tumors of skin (cutaneous and subcutaneous). A shorter survival was found for cases with higher MCs in 21 of 23 studies (91%), while the results in 2 of 23 studies (9%) did not reach statistical significance. A relevant discriminant ability of the MC is suggested by the area under the receiver operating characteristic curve values of 0.78, 0.79 and 0.82.^10,62,155^ The sensitivity and specificity values for the different proposed cutoff stratifications, reported in 9 individual studies, are illustrated in Fig. 5. Higher MCs were significantly associated with shorter disease-free intervals in 8 of 10 studies (80%) and with occurrence of metastasis or recurrence in 4 of 6 studies (67%).

For cutaneous and oral melanoma, higher MCs indicated shorter survival in 11 of 17 studies (65%). Interestingly, a prognostic significance was found in the analysis of only cutaneous melanoma in 4 of 5 studies (80%), analysis of only oral melanoma in 4 of 9 studies (44%), and analysis of both locations in 4 of 4 studies (100%). For cutaneous and oral melanoma, a good discriminant ability of the MC is inferred, based on the provided area under the curve values of 0.78 and 0.86.^9,114^ Shorter disease progression was significantly associated with higher MCs in 3 of 5 studies (60%).

Studies on soft tissue tumors of the skin and subcutis reached statistical significance regarding higher MCs and shorter survival in 6 of 7 studies (86%). The discriminant ability of the MC is not well demonstrated in these studies. Recurrence was associated with higher MCs in 7 of 10 studies (70%).

Studies on splenic stromal sarcoma and the former broader category “fibrohistiocytic nodules” found a significant prognostic association of higher MC values with shorter survival in 3 of 4 (75%) instances. The failure to reach significance in 1 study could be due to a small study population of 8 tumors.

### MI: Risk of Bias, MI Methods, and Prognostic Value

The MI was determined in 5 studies that each evaluated a different tumor type: skeletal osteosarcoma,^98^ multicentric lymphoma,^113^ malignant mammary tumors,^128^ mast cell tumors of the skin,^138^ and aortic body tumors.^164^ The overall risk of bias of these articles was judged to be high (*n* = 4) or moderate (*n* = 1), particularly pertaining to the data analysis domain (Supplemental Table S8).

The MI determination methods varied among studies. Four studies described the staining method, of which 2 used hematoxylin and eosin,^113,164^ 1 employed toluidine blue,^128^ and 1 utilized anti-Proliferating Cell Nuclear Antigen (PCNA) immunolabeled slides.^138^ The proportion of mitotic figures was calculated among various numbers of tumor cells: 500,^113^ 1000 (in PCNA hotspot^138^ or peripheral areas^98^), at least 10,000,^164^ and all cells within 10 hotspot high-power fields (at 25× magnification).^128^ Two studies created photomicrographs for counting,^128,164^ with 1 also using software for cell number estimation.^128^

None of the studies reached statistical significance for the association of the MI with survival time (*N* = 2), metastasis (*N* = 3), or relapse-free interval (*N* = 1; Supplemental Table S9). Only 1 study on mast cell tumors of the skin determined that higher MIs were significantly associated with tumor recurrence.^138^

## Discussion

The prognostic relevance of mitotic activity has been evaluated in many studies on canine tumors, enabling this extensive systematic review. Canine studies on this topic were more numerous than feline studies, encompassing more than 3 times the numbers of articles,^15^ and studies for other animal species are almost nonexistent.^6,67^ This underscores an apparently greater research interest in canine tumors. Nevertheless, the findings of this systematic review on canine tumors were similar to the findings of the previous systematic review on feline tumors regarding the risk of bias in the studies.^15^ For many tumor types, the prognostic relevance of mitotic activity is still not convincingly proven, considering the study limitations and lack of validation studies, as will be discussed below. Since our literature search for this systematic review, several articles on the MC have been published,^14,22,30,38,50,51,66,76,77,85,124,152^ and the number of new references is expected to markedly increase with time, given the enormous increase of research interest over the last decade. Repetition of this systematic review will be required when new evidence-based literature allows new conclusions to be drawn on the prognostic value of the MC and MI in canine tumors.

The MC reflects the routine method of measuring mitotic activity with microscopic tumor evaluation. However, a wide variety of MC methods have been applied in previous studies, and often the methods have not been described in sufficient detail. Each study should describe the key aspects of the MC methods, including the selection of the region of interest, the area (in mm^2^) assessed, and the spatial arrangement of fields evaluated. While the best method regarding reproducibility and prognostic ability is currently unknown, standardization, as previously proposed,^94^ may improve comparability between studies and unify the diagnostic workflow for different tumor types, that is, diagnostic pathologists need to apply the MC methods described in the study upon which they base their prognostic interpretation. However, future studies are needed to determine which of these methods have the highest prognostic value and reproducibility between pathologists.^130^

Many laboratories have completely switched to digital microscopy^16^ and despite the lack of fine focus in digital images (unless scanned with z-stacking), high consistency with light microscopy has been shown for MCs by several studies.^43^ Nevertheless, only a few prognostic studies on canine tumors have used digital microscopy and associated the digital counts with outcome.^14,159^ Digital microscopy has particular requirements when quantifying mitotic activity formerly based on light microscopy, such as the differences in size of high-power fields, which is dependent on the monitor size and display resolutions.^16,68^ Further studies are needed to determine the minimum quality of digital images (resolution and so on) to ensure that mitotic figures are not confused with imposters (such as necrotic cells) and whether the established prognostic thresholds are appropriate. However, digital images also introduce new possibilities for standardized assessment of mitotic figures, such as counting tools in viewing software and image analysis algorithms.^12,13^ In particular, deep learning–based algorithms are considered promising for improving time efficiency, reproducibility, and accuracy for this task (computer-assisted MCs),^4,12^ and further research on the reliable application of these algorithms and the prognostic benefit as compared with the routine approach is needed.

Tumor cell proliferation is 1 key driver of tumorigenesis, and thus, the MC is often assumed to correlate with outcome. However, a surprisingly high proportion of studies did not find a prognostic value (using hypothesis testing) for the MC in canine tumors. It should be noted that interpretation of these results is difficult because many studies restricted their analysis to tests of significance (*P* value approach), which cannot be used to establish a prognostic value (effect size) or lack thereof.^18^ A *P* value above .05 could still indicate a useful prognostic value, particularly if the study population and/or event rate available for analysis was low. Not providing the actual *P* value (instead “not significant” or “*P* > .05” is often reported) hinders interpretation and comparison of the data. We recommend correlating the MC with relevant endpoints by multiple statistical methods including the Kaplan-Meier curves, hazard ratios, sensitivity, and specificity. Cutoff agnostic methods, like receiver operating characteristic curves and their area under the curves, are particularly preferred.^18^

Of note, conflicting findings between studies were found for most of the evaluated tumor types/groups. This highlights the general high risk of bias of observational studies and the need for several validation studies before sufficient evidence of the prognostic value of the MC can be guaranteed. Possible explanations for the lack of prognostic relevance in these studies include small study populations, heterogeneous tumor groups (different tumor entities or locations), variable MC methods, and, as discussed above, flawed statistical methods. While we used uniform thresholds to evaluate the risk of bias of the size of the study population, an appropriate sample size used for a study may vary from our criteria based, among others, on the incidence of the tumor. A small study population (defined as including <7 events for our risk of bias criteria) will have the risk of not being representative for the tumor type regarding the association between histological features and biological behavior. However, even studies with a larger study population (defined as including ≥15 events for our risk of bias criteria) have potential sources of bias and the results need to be validated by subsequent studies that use an independent study population.

We have noted that the results for the prognostic relevance of the MC were quite variable between different studies (as demonstrated for mast cell tumors). Besides the aforementioned limitations, the differences in the results can be explained by variability between pathologists in assessing the MC. While a high degree of inconsistency between pathologists has been shown by several studies,^4,12,13,159^ the influence on prognostication is largely unexplored.^130^ For example, it has been determined that some pathologists have a higher sensitivity (as opposed to precision) when distinguishing mitotic figures from imposters resulting in higher MCs, whereas other pathologists have a higher precision (as opposed to sensitivity) resulting in lower MCs.^4,12^ We argue that this variability between pathologists might have an important influence on the prognostically most meaningful cutoff values, possibly resulting in unexpected performance of the prognostic test when applied routinely by various pathologists in a diagnostic setting. A particularly high degree of divergence in prognostic classification can be expected for tumors with borderline mitotic activity and a patchy distribution throughout the tumor section.^13^ Several studies included in this systematic review used multiple pathologists to determine the MC values for each case; however, only 1 study compared the prognostic ability of the individual pathologists’ evaluations.^130^ Further studies on this topic are needed to better understand the impact of a realistic routine diagnostic setting as compared with a standardized research setting with 1 pathologist. We strongly recommend the appropriateness of prognostic thresholds be validated by multiple pathologists and new approaches/methods to improve interobserver reproducibility, such as interlaboratory training and ring trials or computer-assisted assessment, be developed and evaluated.^12^

Based on our systematic review, we conclude that the MC has a prognostic value for canine mast cell tumors of the skin, cutaneous melanoma, soft tissue tumors of the skin and subcutis, and splenic stromal sarcoma. Thus, determination of the MC is recommended during routine diagnostic evaluation of these tumor types. While the discriminant ability seems to be good for mast cell tumors of the skin^10,62,155^ and cutaneous melanoma,^114^ it requires further evaluation for the other tumor types. The results for oral melanomas are inconsistent between studies; however, those studies with a lower risk of bias and appropriate analysis of discriminant ability suggest a prognostic value of the MC.^7,24,59,117,118,156,159^ For anal sac gland adenocarcinoma, the MC truly seems to have little prognostic value based on several studies.^103,115,141,147^ The results for the prognostic value of the MC are conflicting and not sufficiently supported by statistical analysis of discriminant ability for mammary tumors,^25,26,28,29,40,41,45,81,84,98,119,126,127^ and osteosarcoma^2,34,35,58,99,123,130^ and unproven for all the other tumor types considering the lack of validation studies.

In contrast to the MC, the MI has been rarely evaluated in the literature, which is most likely explained by the inability to apply this to routine diagnostic service. Improved time efficiency of the MI assessment, and thus applicability for routine diagnostics, may be achieved in the future through the use of automated image analysis for tumor cell enumeration. It seems logical that the mitotic activity measurement is more representative for the case when set in relation to the cellular density, particularly in tumor types (such as mammary carcinoma) that exhibit variable cellular density due to extensive extracellular matrix, cystic spaces, inflammation, or edema. Surprisingly, the few studies on canine tumors that evaluated MI did not find a significant association with survival or metastasis, in contrast to the studies on feline mammary tumors.^15^ However, the canine studies did not compare the MI with the MC, and validation studies for each tumor type are not available.

## Conclusions

Mitotic activity is a relevant prognostic test that has been evaluated in many studies on canine tumors. While the MI is rarely determined and its prognostic value is largely unexplored, the discriminant ability of the MC with regard to patient outcome has been well demonstrated in some canine tumors (particularly mast cell tumors of the skin, cutaneous and oral melanoma, and soft tissue tumors of the skin and subcutis). Limitations of current studies include small case numbers, combined evaluation of heterogeneous tumor groups, unavailable details of the MC methods, statistical analysis restricted to the *P* value approach (often without reporting the actual *P* value), and prognostic cutoffs based on single pathologist’s evaluations. Repetition of this systematic review will be needed in several years to update conclusions and recommendations. We highlight the need for development and validation of methods that improve observer reproducibility, such as deep learning–based algorithms (computer-assisted prognosis).

## Supplemental Material

sj-pdf-1-vet-10.1177_03009858241239565 – Supplemental material for Mitotic activity: A systematic literature review of the assessment methodology and prognostic value in canine tumors Supplemental material, sj-pdf-1-vet-10.1177_03009858241239565 for Mitotic activity: A systematic literature review of the assessment methodology and prognostic value in canine tumors by Christof A. Bertram, Taryn A. Donovan and Alexander Bartel in Veterinary Pathology

## References

[1] Able H Wolf-Ringwall A Rendahl A , et al. Computed tomography radiomic features hold prognostic utility for canine lung tumors: an analytical study. PLoS ONE. 2021;16:e0256139. doi:10.1371/journal.pone.0256139. PMC8370631 34403435

[2] Amsellem PM Selmic LE Wypij JM , et al. Appendicular osteosarcoma in small-breed dogs: 51 cases (1986-2011). J Am Vet Med Assoc. 2014;245:203–210. doi:10.2460/javma.245.2.203. 24984131

[3] Aresu L Agnoli C Nicoletti A , et al. Phenotypical characterization and clinical outcome of canine Burkitt-like lymphoma. Front Vet Sci. 2021;8:647009. doi:10.3389/fvets.2021.647009. 33816589 PMC8010238

[4] Aubreville M Stathonikos N Bertram CA , et al. Mitosis domain generalization in histopathology images—the MIDOG challenge. Med Image Anal. 2023;84:102699. doi:10.1016/j.media.2022.102699. 36463832

[5] Avallone G Pellegrino V Muscatello LV , et al. Canine smooth muscle tumors: a clinicopathological study. Vet Pathol. 2022;59:244–255. doi:10.1177/03009858211066862. 34955045

[6] Bacci B Stent AW Walmsley EA. Equine intestinal lymphoma: clinical-pathological features, immunophenotype, and survival. Vet Pathol. 2020;57:369–376. doi:10.1177/0300985820906889. 32202217

[7] Baja AJ Kelsey KL Rusler DM , et al. A retrospective study of 101 dogs with oral melanoma treated with a weekly or biweekly 6 Gy × 6 radiotherapy protocol. Vet Comp Oncol. 2022;20:623–631. doi:10.1111/vco.12815. 35338766 PMC9539951

[8] Berger EP Johannes CM Jergens AE , et al. Retrospective evaluation of toceranib phosphate (Palladia®) use in the treatment of gastrointestinal stromal tumors of dogs. J Vet Intern Med. 2018;32:2045–2053. doi:10.1111/jvim.15335. 30307656 PMC6271363

[9] Bergin IL Smedley RC Esplin DG , et al. Prognostic evaluation of Ki67 threshold value in canine oral melanoma. Vet Pathol. 2011;48:41–53. doi:10.1177/0300985810388947. 21123859

[10] Berlato D Murphy S Laberke S , et al. Comparison of minichromosome maintenance protein 7, Ki67 and mitotic index in the prognosis of intermediate Patnaik grade cutaneous mast cell tumours in dogs. Vet Comp Oncol. 2018;16:535–543. doi:10.1111/vco.12412. 29989314

[11] Berlato D Murphy S Monti P , et al. Comparison of mitotic index and Ki67 index in the prognostication of canine cutaneous mast cell tumours. Vet Comp Oncol. 2015;13:143–150. doi:10.1111/vco.12029. 23489679

[12] Bertram CA Aubreville M Donovan TA , et al. Computer-assisted mitotic count using a deep learning-based algorithm improves interobserver reproducibility and accuracy. Vet Pathol. 2022;59:211–226. doi:10.1177/03009858211067478. 34965805 PMC8928234

[13] Bertram CA Aubreville M Gurtner C , et al. Computerized calculation of mitotic count distribution in canine cutaneous mast cell tumor sections: mitotic count is area dependent. Vet Pathol. 2020;57:214–226. doi:10.1177/0300985819890686. 31808382

[14] Bertram CA Bartel A Donovan TA , et al. Atypical mitotic figures are prognostically meaningful for canine cutaneous mast cell tumors. Vet Sci. 2024;11:5. 10.3390/vetsci11010005 PMC10821277 38275921

[15] Bertram CA Donovan TA Bartel A . Mitotic activity: a systematic review of the assessment methodology and prognostic value in feline tumors. Vet Pathol. 2024. doi:10.1177/03009858241239566. 10.1177/03009858241239565 PMC11370189 38533804

[16] Bertram CA Stathonikos N Donovan TA , et al. Validation of digital microscopy: review of validation methods and sources of bias. Vet Pathol. 2022;59:26–38. doi:10.1177/03009858211040476. 34433345 PMC8761960

[17] Boos GS Bassuino DM Wurster F , et al. Retrospective canine skin peripheral nerve sheath tumors data with emphasis on histologic, immunohistochemical and prognostic factors. Pesqui Vet Bras. 2015;35:965–974. doi:10.1590/S0100-736X2015001200005.

[18] Boracchi P Roccabianca P Avallone G , et al. Kaplan-Meier curves, Cox model, and P-values are not enough for the prognostic evaluation of tumor markers: statistical suggestions for a more comprehensive approach. Vet Pathol. 2021;58:795–808. doi:10.1177/03009858211014174. 33977800

[19] Bostock DE. Prognosis after surgical excision of canine melanomas. Vet Pathol. 1979;16:32–40. 462717 10.1177/030098587901600103

[20] Bostock DE Crocker J Harris K , et al. Nucleolar organiser regions as indicators of post-surgical prognosis in canine spontaneous mast cell tumours. Br J Cancer. 1989;59:915–918. doi:10.1038/bjc.1989.193. 2500145 PMC2246742

[21] Bostock DE Dye MT. Prognosis after surgical excision of canine fibrous connective tissue sarcomas. Vet Pathol. 1980;17:581–588. doi:10.1177/030098588001700507. 7404969

[22] Bray JP Munday JS. Development of a nomogram to predict the outcome for patients with soft tissue sarcoma. Vet Sci. 2023;10:266. doi:10.3390/vetsci10040266. 37104421 PMC10146366

[23] Bray JP Polton GA McSporran KD , et al. Canine soft tissue sarcoma managed in first opinion practice: outcome in 350 cases. Vet Surg. 2014;43:774–782. doi:10.1111/j.1532-950X.2014.12185.x. 24724565

[24] Camerino M Giacobino D Manassero L , et al. Prognostic impact of bone invasion in canine oral malignant melanoma treated by surgery and anti-CSPG4 vaccination: a retrospective study on 68 cases (2010-2020). Vet Comp Oncol. 2022;20:189–197. doi:10.1111/vco.12761. 34392602 PMC9290081

[25] Canadas A França M Pereira C , et al. Canine mammary tumors: comparison of classification and grading methods in a survival study. Vet Pathol. 2019;56:208–219. doi:10.1177/0300985818806968. 30381007

[26] Carvalho MI Pires I Prada J , et al. Assessing the interleukin 35 immunoexpression in malignant canine mammary tumors: association with clinicopathological parameters and prognosis. Anticancer Res. 2019;39:2077–2083. doi:10.21873/anticanres.13319. 30952752

[27] Carvalho MI Pires I Prada J , et al. Ki-67 and PCNA expression in canine mammary tumors and adjacent nonneoplastic mammary glands: prognostic impact by a multivariate survival analysis. Vet Pathol. 2016;53:1138–1146. doi:10.1177/0300985816646429. 27162119

[28] Carvalho S Stoll AL Priestnall SL , et al. Retrospective evaluation of COX-2 expression, histological and clinical factors as prognostic indicators in dogs with renal cell carcinomas undergoing nephrectomy. Vet Comp Oncol. 2017;15:1280–1294. doi:10.1111/vco.12264. 27578604

[29] Chen YC Chen YY Liao JW , et al. Expression and prognostic value of c-met in canine mammary tumours. Vet Comp Oncol. 2018;16:670–676. doi:10.1111/vco.12439. 30129270

[30] Cherzan NL Fryer K Burke B , et al. Factors affecting prognosis in canine subcutaneous mast cell tumors: 45 cases. Vet Surg. 2023;52:531–537. doi:10.1111/vsu.13944. 36788161

[31] Chiti LE Ferrari R Boracchi P , et al. Prognostic impact of clinical, haematological, and histopathological variables in 102 canine cutaneous perivascular wall tumours. Vet Comp Oncol. 2021;19:275–283. doi:10.1111/vco.12673. 33386693

[32] Chiti LE Ferrari R Roccabianca P , et al. Surgical margins in canine cutaneous soft-tissue sarcomas: a dichotomous classification system does not accurately predict the risk of local recurrence. Animals (Basel). 2021;11:2367. doi:10.3390/ani11082367. 34438827 PMC8388623

[33] Cleland NT Morton J Delisser PJ. Outcome after surgical management of canine insulinoma in 49 cases. Vet Comp Oncol. 2021;19:428–441. doi:10.1111/vco.12628. 32558184

[34] Cook MR Lorbach J Husbands BD , et al. A retrospective analysis of 11 dogs with surface osteosarcoma. Vet Comp Oncol. 2022;20:82–90. doi:10.1111/vco.12741. 34033204

[35] Coyle VJ Rassnick KM Borst LB , et al. Biological behaviour of canine mandibular osteosarcoma. A retrospective study of 50 cases (1999-2007). Vet Comp Oncol. 2015;13:89–97. doi:10.1111/vco.12020. 23410097

[36] Crownshaw AH McEntee MC Nolan MW , et al. Evaluation of variables associated with outcomes in 41 dogs with incompletely excised high-grade soft tissue sarcomas treated with definitive-intent radiation therapy with or without chemotherapy. J Am Vet Med Assoc. 2020;256:783–791. doi:10.2460/javma.256.7.783. 32176583

[37] Del Alcazar CM Mahoney JA Dittrich K , et al. Outcome, prognostic factors and histological characterization of canine gastrointestinal sarcomas. Vet Comp Oncol. 2021;19:578–586. doi:10.1111/vco.12696. 33774909

[38] Dettwiler M Mauldin EA Jastrebski S , et al. Prognostic clinical and histopathological features of canine cutaneous epitheliotropic T-cell lymphoma. Vet Pathol. 2023;60:162–171. doi:10.1177/03009858221140818. 36541607

[39] Dobson JM Blackwood LB McInnes EF , et al. Prognostic variables in canine multicentric lymphosarcoma. J Small Anim Pract. 2001;42:377–384. doi:10.1111/j.1748-5827.2001.tb02485.x. 11518416

[40] Dolka I Czopowicz M Gruk-Jurka A , et al. Diagnostic efficacy of smear cytology and Robinson’s cytological grading of canine mammary tumors with respect to histopathology, cytomorphometry, metastases and overall survival. PLoS ONE. 2018;13:e0191595. doi:10.1371/journal.pone.0191595. PMC5779680 29360854

[41] Dolka I Król M Sapierzyński R. Evaluation of apoptosis-associated protein (Bcl-2, Bax, cleaved caspase-3 and p53) expression in canine mammary tumors: an immunohistochemical and prognostic study. Res Vet Sci. 2016;105:124–133. doi:10.1016/j.rvsc.2016.02.004. 27033920

[42] Donnelly L Mullin C Balko J , et al. Evaluation of histological grade and histologically tumour-free margins as predictors of local recurrence in completely excised canine mast cell tumours. Vet Comp Oncol. 2015;13:70–76. doi:10.1111/vco.12021. 23451809

[43] Donovan TA Moore FM Bertram CA , et al. Mitotic figures-normal, atypical, and imposters: a guide to identification. Vet Pathol. 2021;58:243–257. doi:10.1177/0300985820980049. 33371818

[44] Dunn JK Bostock DE Herrtage ME , et al. Insulin-secreting tumours of the canine pancreas: clinical and pathological features of 11 cases. J Small Anim Pract. 1993;34:325–331. doi:10.1111/j.1748-5827.1993.tb02704.x.

[45] Dutra AP Azevedo Júnior GM Schmitt FC , et al. Assessment of cell proliferation and prognostic factors in canine mammary gland tumors. Arq Bras Med Vet Zootec. 2008;60:1403–1412.

[46] Edmondson EF Hess AM Powers BE. Prognostic significance of histologic features in canine renal cell carcinomas: 70 nephrectomies. Vet Pathol. 2015;52:260–268. doi:10.1177/0300985814533803. 24829287

[47] Elliott JW Cripps P Blackwood L , et al. Canine oral mucosal mast cell tumours. Vet Comp Oncol. 2016;14:101–111. doi:10.1111/vco.12071. 24215587

[48] Elston LB Sueiro FA Cavalcanti JN , et al. The importance of the mitotic index as a prognostic factor for survival of canine cutaneous mast cell tumors: a validation study. Vet Pathol. 2009;46:362–364. doi:10.1354/vp.46-2-362. 19261652

[49] Ettinger SN Scase TJ Oberthaler KT , et al. Association of argyrophilic nucleolar organizing regions, Ki-67, and proliferating cell nuclear antigen scores with histologic grade and survival in dogs with soft tissue sarcomas: 60 cases (1996-2002). J Am Vet Med Assoc. 2006;228:1053–1062. doi:10.2460/javma.228.7.1053. 16579784

[50] Evenhuis JV Oates A Hoyer N , et al. A retrospective study of canine oral extramedullary plasmacytoma over a 15-year period (July 2004-July 2019): treatment, histologic parameters and clinical outcomes. Vet Comp Oncol. 2023;21:302–314. doi:10.1111/vco.12888. 36808816

[51] Ferrari R Marconato L Boracchi P , et al. Splenic stromal sarcomas in dogs: outcome and clinicopathological prognostic factors in 32 cases. Vet Comp Oncol. 2023;**22**:12–21. doi:10.1111/vco.12941. 37918913

[52] Flood-Knapik KE Durham AC Gregor TP , et al. Clinical, histopathological and immunohistochemical characterization of canine indolent lymphoma. Vet Comp Oncol. 2013;11:272–286. doi:10.1111/j.1476-5829.2011.00317.x. 22296667

[53] Gill V Leibman N Monette S , et al. Prognostic indicators and clinical outcome in dogs with subcutaneous mast cell tumors treated with surgery alone: 43 cases. J Am Anim Hosp Assoc. 2020;56:215–225. doi:10.5326/JAAHA-MS-6960. 32412337

[54] Gillespie V Baer K Farrelly J , et al. Canine gastrointestinal stromal tumors: immunohistochemical expression of CD34 and examination of prognostic indicators including proliferation markers Ki67 and AgNOR. Vet Pathol. 2011;48:283–291. doi:10.1177/0300985810380397. 20826846

[55] Giuliano EA Chappell R Fischer B , et al. A matched observational study of canine survival with primary intraocular melanocytic neoplasia. Vet Ophthalmol. 1999;2:185–190. doi:10.1046/j.1463-5224.1999.00080.x. 11397263

[56] Graves GM Bjorling DE Mahaffey E. Canine hemangiopericytoma: 23 cases (1967-1984). J Am Vet Med Assoc. 1988;192:99–102. 3343190

[57] Gregório H Raposo T Queiroga FL , et al. High COX-2 expression in canine mast cell tumours is associated with proliferation, angiogenesis and decreased overall survival. Vet Comp Oncol. 2017;15:1382–1392. doi:10.1111/vco.12280. 28467670

[58] Guim TN Bianchi MV De Lorenzo C , et al. Relationship between clinicopathological features and prognosis in appendicular osteosarcoma in dogs. J Comp Pathol. 2020;180:91–99. doi:10.1016/j.jcpa.2020.09.003. 33222881

[59] Hahn KA DeNicola DB Richardson RC , et al. Canine oral malignant melanoma: prognostic utility of an alternative staging system. J Small Anim Pract. 1994;35:251–256.

[60] Hammer A Getzy D Ogilvie G , et al. Salivary gland neoplasia in the dog and cat: survival times and prognostic factors. J Am Anim Hosp Assoc. 2001;37:478–482. doi:10.5326/15473317-37-5-478. 11563448

[61] Heller DA Stebbins ME Reynolds TL , et al. A retrospective study of 87 cases of canine soft tissue sarcomas. Int J Appl Res Vet Med. 2005;3:81–87.

[62] Horta RS Lavalle GE Monteiro LN , et al. Assessment of canine mast cell tumor mortality risk based on clinical, histologic, immunohistochemical, and molecular features. Vet Pathol. 2018;55:212–223. doi:10.1177/0300985817747325. 29338615

[63] Hughes KL Ehrhart EJ Rout ED , et al. Diffuse small B-cell lymphoma: a high-grade malignancy. Vet Pathol. 2021;58:912–922. doi:10.1177/0300985820985221. 33461440

[64] Hume CT Kiupel M Rigatti L , et al. Outcomes of dogs with grade 3 mast cell tumors: 43 cases (1997-2007). J Am Anim Hosp Assoc. 2011;47:37–44. doi:10.5326/JAAHA-MS-5557. 21164163

[65] Iwaki Y Lindley S Smith A , et al. Canine myxosarcomas, a retrospective analysis of 32 dogs (2003-2018). BMC Vet Res. 2019;15:217. doi:10.1186/s12917-019-1956-z. 31248415 PMC6595552

[66] Jeon MD Leeper HJ Cook MR , et al. Multi-institutional retrospective study of canine foot pad malignant melanomas: 20 cases. Vet Comp Oncol. 2022;20:854–861. doi:10.1111/vco.12846. 35771690

[67] Karalus W Subharat S Orbell G , et al. Equine sarcoids: A clinicopathologic study of 49 cases, with mitotic count and clinical type predictive of recurrence. Vet Pathol. 2023;0(0). doi:10.1177/03009858231209408. PMC11067406 37937724

[68] Kim D Pantanowitz L Schüffler P , et al. (Re) defining the high-power field for digital pathology. J Pathol Inform. 2020;11:33. doi:10.4103/jpi.jpi_48_20. 33343994 PMC7737490

[69] Kim SE Liptak JM Gall TT , et al. Epirubicin in the adjuvant treatment of splenic hemangiosarcoma in dogs: 59 cases (1997-2004). J Am Vet Med Assoc. 2007;231:1550–1557. doi:10.2460/javma.231.10.1550. 18021000

[70] Kirpensteijn J Kik M Rutteman GR , et al. Prognostic significance of a new histologic grading system for canine osteosarcoma. Vet Pathol. 2002;39:240–246. doi:10.1354/vp.39-2-240. 12009062

[71] Kiupel M Teske E Bostock D. Prognostic factors for treated canine malignant lymphoma. Vet Pathol. 1999;36:292–300. 10421095 10.1354/vp.36-4-292

[72] Knight BJ Wood GA Foster RA , et al. Beclin-1 is a novel predictive biomarker for canine cutaneous and subcutaneous mast cell tumors. Vet Pathol. 2022;59:46–56. doi:10.1177/03009858211042578. 34521293 PMC8679166

[73] Krick EL Kiupel M Durham AC , et al. Investigating associations between proliferation indices, c-kit, and lymph node stage in canine mast cell tumors. J Am Anim Hosp Assoc. 2017;53:258–264. doi:10.5326/JAAHA-MS-6265. 28792799

[74] Kuntz CA Dernell WS Powers BE , et al. Prognostic factors for surgical treatment of soft-tissue sarcomas in dogs: 75 cases (1986-1996). J Am Vet Med Assoc. 1997;211:1147–1151. 9364229

[75] Laprie C Abadie J Amardeilh MF , et al. MIB-1 immunoreactivity correlates with biologic behaviour in canine cutaneous melanoma. Vet Dermatol. 2001;12:139–147. doi:10.1046/j.1365-3164.2001.00236.x. 11420929

[76] Lapsley JM Wavreille V Barry S , et al. Risk factors and outcome in dogs with recurrent massive hepatocellular carcinoma: a Veterinary Society of Surgical Oncology case-control study. Vet Comp Oncol. 2022;20:697–709. doi:10.1111/vco.12824. 35488436 PMC9546275

[77] Larsen E Watson AM Muñoz Gutiérrez JF. Intranasal mast cell tumors: clinical, immunohistochemical, and molecular features in 20 dogs. Vet Pathol. 2022;59:915–921. doi:10.1177/03009858221109100. 35787192

[78] Lascelles BD Parry AT Stidworthy MF , et al. Squamous cell carcinoma of the nasal planum in 17 dogs. Vet Rec. 2000;147:473–476. doi:10.1136/vr.147.17.473. 11093398

[79] Laver T Feldhaeusser BR Robat CS , et al. Post-surgical outcome and prognostic factors in canine malignant melanomas of the haired skin: 87 cases (2003-2015). Can Vet J. 2018;59:981–987. 30197441 PMC6091115

[80] Linden D Liptak JM Vinayak A , et al. Outcomes and prognostic variables associated with primary abdominal visceral soft tissue sarcomas in dogs: a Veterinary Society of Surgical Oncology retrospective study. Vet Comp Oncol. 2019;17:265–270. doi:10.1111/vco.12456. 30666781

[81] Liu JL Chang KC Lo CC , et al. Expression of autophagy-related protein Beclin-1 in malignant canine mammary tumors. BMC Vet Res. 2013;9:75. doi:10.1186/1746-6148-9-75. 23578251 PMC3639141

[82] Loukopoulos P Robinson WF. Clinicopathological relevance of tumour grading in canine osteosarcoma. J Comp Pathol. 2007;136:65–73. doi:10.1016/j.jcpa.2006.11.005. 17270206

[83] Maas CP ter Haar G van der Gaag I , et al. Reclassification of small intestinal and cecal smooth muscle tumors in 72 dogs: clinical, histologic, and immunohistochemical evaluation. Vet Surg. 2007;36:302–313. doi:10.1111/j.1532-950X.2007.00271.x. 17547593

[84] Mainenti M Rasotto R Carnier P , et al. Oestrogen and progesterone receptor expression in subtypes of canine mammary tumours in intact and ovariectomised dogs. Vet J. 2014;202:62–68. doi:10.1016/j.tvjl.2014.06.003. 24980810

[85] Marconato L Stefanello D Solari Basano F , et al. Subcutaneous mast cell tumours: a prospective multi-institutional clinicopathological and prognostic study of 43 dogs. Vet Rec. 2023;193:e2991. doi:10.1002/vetr.2991. 37224084

[86] Martínez CM Peñafiel-Verdú C Vilafranca M , et al. Cyclooxygenase-2 expression is related with localization, proliferation, and overall survival in canine melanocytic neoplasms. Vet Pathol. 2011;48:1204–1211. doi:10.1177/0300985810396517. 21292918

[87] McNiel EA Ogilvie GK Powers BE , et al. Evaluation of prognostic factors for dogs with primary lung tumors: 67 cases (1985-1992). J Am Vet Med Assoc. 1997;211:1422–1427. 9394893

[88] McNiel EA Prink AL O’Brien TD. Evaluation of risk and clinical outcome of mast cell tumours in pug dogs. Vet Comp Oncol. 2006;4:2–8. doi:10.1111/j.1476-5810.2006.00085.x. 19754824

[89] McPhetridge JB Scharf VF Regier PJ , et al. Distribution of histopathologic types of primary pulmonary neoplasia in dogs and outcome of affected dogs: 340 cases (2010-2019). J Am Vet Med Assoc. 2021;260:234–243. doi:10.2460/javma.20.12.0698. 34851850

[90] McSporran KD. Histologic grade predicts recurrence for marginally excised canine subcutaneous soft tissue sarcomas. Vet Pathol. 2009;46:928–933. doi:10.1354/vp.08-VP-0277-M-FL. 19429989

[91] Mendez SE Sykes Crumplar SE Durham AC. Primary hemangiosarcoma of the falciform fat in seven dogs (2007-2015). J Am Anim Hosp Assoc. 2020;56:120–126. doi:10.5326/jaaha-ms-6967. 31961215

[92] Merickel JL Pluhar GE Rendahl A , et al. Prognostic histopathologic features of canine glial tumors. Vet Pathol. 2021;58:945–951. doi:10.1177/03009858211025795. 34219560 PMC10923237

[93] Meuten D Munday JS Hauck M. Time to standardize? time to validate? Vet Pathol. 2018;55:195–199. doi:10.1177/0300985817753869. 29457567

[94] Meuten DJ Moore FM Donovan TA , et al. International guidelines for veterinary tumor pathology: a call to action. Vet Pathol. 2021;58:766–794. doi:10.1177/03009858211013712. 34282984

[95] Meuten DJ Moore FM George JW. Mitotic count and the field of view area: time to standardize. Vet Pathol. 2016;53:7–9. doi:10.1177/0300985815593349. 26712813

[96] Millanta F Fratini F Corazza M , et al. Proliferation activity in oral and cutaneous canine melanocytic tumours: correlation with histological parameters, location, and clinical behaviour. Res Vet Sci. 2002;73:45–51. doi:10.1016/s0034-5288(02)00041-3. 12208106

[97] Misdorp W Hart AA. Prognostic factors in canine mammary cancer. J Natl Cancer Inst. 1976;56:779–786. doi:10.1093/jnci/56.4.779. 1255797

[98] Misdorp W Hart AA. Some prognostic and epidemiologic factors in canine osteosarcoma. J Natl Cancer Inst. 1979;62:537–545. doi:10.1093/jnci/62.3.537. 283283

[99] Moore AS Dernell WS Ogilvie GK , et al. Doxorubicin and BAY12-9566 for the treatment of osteosarcoma in dogs: a randomized, double-blind, placebo-controlled study. J Vet Intern Med. 2007;21:783–790. doi:10.1892/0891-6640(2007)21[783:dabftt]2.0.co;2. 17708400

[100] Moore AS Frimberger AE Sullivan N , et al. Histologic and immunohistochemical review of splenic fibrohistiocytic nodules in dogs. J Vet Intern Med. 2012;26:1164–1168. doi:10.1111/j.1939-1676.2012.00986.x. 22882592

[101] Moore AS Frimberger AE Taylor D , et al. Retrospective outcome evaluation for dogs with surgically excised, solitary Kiupel high-grade, cutaneous mast cell tumours. Vet Comp Oncol. 2020;18:402–408. doi:10.1111/vco.12565. 31916687

[102] Moore AS Rassnick KM Frimberger AE. Evaluation of clinical and histologic factors associated with survival time in dogs with stage II splenic hemangiosarcoma treated by splenectomy and adjuvant chemotherapy: 30 cases (2011-2014). J Am Vet Med Assoc. 2017;251:559–565. doi:10.2460/javma.251.5.559. 28828962

[103] Morello EM Cino M Giacobino D , et al. Prognostic value of ki67 and other clinical and histopathological factors in canine apocrine gland anal sac adenocarcinoma. Animals. 2021;1:1649. doi:10.3390/ani11061649. PMC8228493 34199347

[104] Nakagaki KYR Nunes MM Garcia APV , et al. Neuroendocrine carcinomas of the canine mammary gland: histopathological and immunohistochemical characteristics. Front Vet Sci. 2020;7:621714. doi:10.3389/fvets.2020.621714. 33469557 PMC7813755

[105] Newman SJ Mrkonjich L Walker KK , et al. Canine subcutaneous mast cell tumour: diagnosis and prognosis. J Comp Pathol. 2007;136:231–239. doi:10.1016/j.jcpa.2007.02.003. 17399734

[106] Nóbrega DF Sehaber VF Madureira R , et al. Canine cutaneous haemangiosarcoma: biomarkers and survival. J Comp Pathol. 2019;166:87–96. doi:10.1016/j.jcpa.2018.10.181. 30691610

[107] O’Connell K Thomson M. Evaluation of prognostic indicators in dogs with multiple, simultaneously occurring cutaneous mast cell tumours: 63 cases. Vet Comp Oncol. 2013;11:51–62. doi:10.1111/j.1476-5829.2011.00301.x. 22235766

[108] Ogilvie GK Powers BE Mallinckrodt CH , et al. Surgery and doxorubicin in dogs with hemangiosarcoma. J Vet Intern Med. 1996;10:379–384. doi:10.1111/j.1939-1676.1996.tb02085.x. 8947871

[109] Ouzzani M Hammady H Fedorowicz Z , et al. Rayyan—a web and mobile app for systematic reviews. Syst Rev. 2016;5:210. Accessed March 7, 2024. https://rayyan.ai/. 27919275 10.1186/s13643-016-0384-4 PMC5139140

[110] Page MJ McKenzie JE Bossuyt PM , et al. The PRISMA 2020 statement: an updated guideline for reporting systematic reviews. BMJ. 2021;372:n71. doi:10.1136/bmj.n71. PMC8005924 33782057

[111] Pazzi P Kavkovsky A Shipov A , et al. Spirocerca lupi induced oesophageal neoplasia: predictors of surgical outcome. Vet Parasitol. 2018;250:71–77. doi:10.1016/j.vetpar.2017.11.013. 29329628

[112] Pecceu E Serra Varela JC Handel I , et al. Ultrasound is a poor predictor of early or overt liver or spleen metastasis in dogs with high-risk mast cell tumours. Vet Comp Oncol. 2020;18:389–401. doi:10.1111/vco.12563. 31863546

[113] Phillips BS Kass PH Naydan DK , et al. Apoptotic and proliferation indexes in canine lymphoma. J Vet Diagn Invest. 2000;12:111–117. doi:10.1177/104063870001200202. 10730938

[114] Porcellato I Brachelente C Cappelli K , et al. FoxP3, CTLA-4, and IDO in canine melanocytic tumors. Vet Pathol. 2021;58:42–52. doi:10.1177/0300985820960131. 33021155

[115] Pradel J Berlato D Dobromylskyj M , et al. Prognostic significance of histopathology in canine anal sac gland adenocarcinomas: preliminary results in a retrospective study of 39 cases. Vet Comp Oncol. 2018;16:518–528. doi:10.1111/vco.12410. 29961964

[116] Preziosi R Sarli G Paltrinieri M. Prognostic value of intratumoral vessel density in cutaneous mast cell tumors of the dog. J Comp Pathol. 2004;130:143–151. doi:10.1016/j.jcpa.2003.10.003. 15003472

[117] Prouteau A Chocteau F de Brito C , et al. Prognostic value of somatic focal amplifications on chromosome 30 in canine oral melanoma. Vet Comp Oncol. 2020;18:214–223. doi:10.1111/vco.12536. 31461207

[118] Ramos-Vara JA Beissenherz ME Miller MA , et al. Retrospective study of 338 canine oral melanomas with clinical, histologic, and immunohistochemical review of 129 cases. Vet Pathol. 2000;37:597–608. doi:10.1354/vp.37-6-597. 11105949

[119] Ressel L Puleio R Loria GR , et al. HER-2 expression in canine morphologically normal, hyperplastic and neoplastic mammary tissues and its correlation with the clinical outcome. Res Vet Sci. 2013;94:299–305. doi:10.1016/j.rvsc.2012.09.016. 23141215

[120] Rigas K Biasoli D Polton G , et al. Mast cell tumours in dogs less than 12 months of age: a multi-institutional retrospective study. J Small Anim Pract. 2020;61:449–457. doi:10.1111/jsap.13181. 32715502

[121] Robinson WP Elliott J Baines SJ , et al. Intramuscular mast cell tumors in 7 dogs. Can Vet J. 2017;58:931–935. 28878416 PMC5556469

[122] Romansik EM Reilly CM Kass PH , et al. Mitotic index is predictive for survival for canine cutaneous mast cell tumors. Vet Pathol. 2007;44:335–341. doi:10.1354/vp.44-3-335. 17491075

[123] Saam DE Liptak JM Stalker MJ , et al. Predictors of outcome in dogs treated with adjuvant carboplatin for appendicular osteosarcoma: 65 cases (1996-2006). J Am Vet Med Assoc. 2011;238:195–206. doi:10.2460/javma.238.2.195. 21235373

[124] Sabattini S Rigillo A Foiani G , et al. Clinicopathologic features and biologic behavior of canine splenic nodules with stromal, histiocytic and lymphoid components. Front Vet Sci. 2022;9:962685. doi:10.3389/fvets.2022.962685. 36032303 PMC9411940

[125] Sánchez J Ramirez GA Buendia AJ , et al. Immunohistochemical characterization and evaluation of prognostic factors in canine oral melanomas with osteocartilaginous differentiation. Vet Pathol. 2007;44:676–682. doi:10.1354/vp.44-5-676. 17846240

[126] Santos AA Lopes CC Ribeiro JR , et al. Identification of prognostic factors in canine mammary malignant tumours: a multivariable survival study. BMC Vet Res. 2013;9:1. doi:10.1186/1746-6148-9-1. 23289974 PMC3542312

[127] Santos M Correia-Gomes C Marcos R , et al. Value of the Nottingham histological grading parameters and Nottingham prognostic index in canine mammary carcinoma. Anticancer Res. 2015;35:4219–4227. 26124382

[128] Sarli G Preziosi R Benazzi C , et al. Prognostic value of histologic stage and proliferative activity in canine malignant mammary tumors. J Vet Diagn Invest. 2002;14:25–34. doi:10.1177/104063870201400106. 12680640

[129] Schlag AN Johnson T Vinayak A , et al. Comparison of methods to determine primary tumour size in canine apocrine gland anal sac adenocarcinoma. J Small Anim Pract. 2020;61:185–189. doi:10.1111/jsap.13104. 31960434

[130] Schott CR Tatiersky LJ Foster RA , et al. Histologic grade does not predict outcome in dogs with appendicular osteosarcoma receiving the standard of care. Vet Pathol. 2018;55:202–211. doi:10.1177/0300985817747329. 29284372

[131] Schultheiss PC. A retrospective study of visceral and nonvisceral hemangiosarcoma and hemangiomas in domestic animals. J Vet Diagn Invest. 2004;16:522–526. doi:10.1177/104063870401600606. 15586567

[132] Schultheiss PC. Histologic features and clinical outcomes of melanomas of lip, haired skin, and nail bed locations of dogs. J Vet Diagn Invest. 2006;18:422–425. doi:10.1177/104063870601800422. 16921890

[133] Schwab TM Popovitch C DeBiasio J , et al. Clinical outcome for MCTs of canine pinnae treated with surgical excision (2004-2008). J Am Anim Hosp Assoc. 2014;50:187–191. doi:10.5326/JAAHA-MS-6039. 24659731

[134] Selting KA Powers BE Thompson LJ , et al. Outcome of dogs with high-grade soft tissue sarcomas treated with and without adjuvant doxorubicin chemotherapy: 39 cases (1996-2004). J Am Vet Med Assoc. 2005;227:1442–1448. doi:10.2460/javma.2005.227.1442. 16279389

[135] Shiu KB Flory AB Anderson CL , et al. Predictors of outcome in dogs with subcutaneous or intramuscular hemangiosarcoma. J Am Vet Med Assoc. 2011;238:472–479. doi:10.2460/javma.238.4.472. 21320017

[136] Sierra Matiz OR Santilli J Anai LA , et al. Prognostic significance of Ki67 and its correlation with mitotic index in dogs with diffuse large B-cell lymphoma treated with 19-week CHOP-based protocol. J Vet Diagn Invest. 2018;30:263–267. doi:10.1177/1040638717743280. 29192554 PMC6505881

[137] Silvestri S Porcellato I Mechelli L , et al. Tumor thickness and modified Clark level in canine cutaneous melanocytic tumors. Vet Pathol. 2019;56:180–188. doi:10.1177/0300985818798094. 30244658

[138] Simoes JP Schoning P Butine M. Prognosis of canine mast cell tumors: a comparison of three methods. Vet Pathol. 1994;31:637–647. doi:10.1177/030098589403100602. 7863578

[139] Simon D Ruslander DM Rassnick KM , et al. Orthovoltage radiation and weekly low dose of doxorubicin for the treatment of incompletely excised soft-tissue sarcomas in 39 dogs. Vet Rec. 2007;160:321–326. doi:10.1136/vr.160.10.321. 17351172

[140] Skor O Fuchs-Baumgartinger A Tichy A , et al. Pretreatment leukocyte ratios and concentrations as predictors of outcome in dogs with cutaneous mast cell tumours. Vet Comp Oncol. 2017;15:1333–1345. doi:10.1111/vco.12274. 27723224

[141] Skorupski KA Alarcón CN de Lorimier LP , et al. Outcome and clinical, pathological, and immunohistochemical factors associated with prognosis for dogs with early-stage anal sac adenocarcinoma treated with surgery alone: 34 cases (2002-2013). J Am Vet Med Assoc. 2018;253:84–91. doi:10.2460/javma.253.1.84. 29911942

[142] Sledge DG Webster J Kiupel M. Canine cutaneous mast cell tumors: a combined clinical and pathologic approach to diagnosis, prognosis, and treatment selection. Vet J. 2016;215:43–54. doi:10.1016/j.tvjl.2016.06.003. 27372911

[143] Spangler WL Culbertson MR Kass PH. Primary mesenchymal (nonangiomatous/nonlymphomatous) neoplasms occurring in the canine spleen: anatomic classification, immunohistochemistry, and mitotic activity correlated with patient survival. Vet Pathol. 1994;31:37–47. doi:10.1177/030098589403100105. 8140724

[144] Spangler WL Kass PH. Pathologic and prognostic characteristics of splenomegaly in dogs due to fibrohistiocytic nodules: 98 cases. Vet Pathol. 1998;35:488–498. doi:10.1177/030098589803500603. 9823590

[145] Spangler WL Kass PH. The histologic and epidemiologic bases for prognostic considerations in canine melanocytic neoplasia. Vet Pathol. 2006;43:136–149. doi:10.1354/vp.43-2-136. 16537931

[146] Stefanello D Avallone G Ferrari R , et al. Canine cutaneous perivascular wall tumors at first presentation: clinical behavior and prognostic factors in 55 cases. J Vet Intern Med. 2011;25:1398–1405. doi:10.1111/j.1939-1676.2011.00822.x. 22092634

[147] Tanis JB Simlett-Moss AB Ossowksa M , et al. Canine anal sac gland carcinoma with regional lymph node metastases treated with sacculectomy and lymphadenectomy: outcome and possible prognostic factors. Vet Comp Oncol. 2022;20:276–292. doi:10.1111/vco.12774. 34590408

[148] Thamm DH Weishaar KM Charles JB , et al. Phosphorylated KIT as a predictor of outcome in canine mast cell tumours treated with toceranib phosphate or vinblastine. Vet Comp Oncol. 2020;18:169–175. doi:10.1111/vco.12525. 31365175

[149] Thompson JJ Morrison JA Pearl DL , et al. Receptor tyrosine kinase expression profiles in canine cutaneous and subcutaneous mast cell tumors. Vet Pathol. 2016;53:545–558. doi:10.1177/0300985815610388. 26459517

[150] Thompson JJ Pearl DL Yager JA , et al. Canine subcutaneous mast cell tumor: characterization and prognostic indices. Vet Pathol. 2011;48:156–168. doi:10.1177/0300985810387446. 21078881

[151] Thompson JJ Yager JA Best SJ , et al. Canine subcutaneous mast cell tumors: cellular proliferation and KIT expression as prognostic indices. Vet Pathol. 2011;48:169–181. doi:10.1177/0300985810390716. 21160022

[152] Treggiari E Valenti P Porcellato I , et al. Retrospective analysis of outcome and prognostic factors of subcutaneous mast cell tumours in dogs undergoing surgery with or without adjuvant treatment. Vet Comp Oncol. 2023;21:748. doi:10.1111/vco.12902. 37121954

[153] Vail DM Powers BE Getzy DM , et al. Evaluation of prognostic factors for dogs with synovial sarcoma: 36 cases (1986-1991). J Am Vet Med Assoc. 1994;205:1300–1307. 7698942

[154] Valli VE Kass PH San Myint M , et al. Canine lymphomas: association of classification type, disease stage, tumor subtype, mitotic rate, and treatment with survival. Vet Pathol. 2013;50:738–748. doi:10.1177/0300985813478210. 23444036

[155] van Lelyveld S Warland J Miller R , et al. Comparison between Ki-67 index and mitotic index for predicting outcome in canine mast cell tumours. J Small Anim Pract. 2015;56:312–319. doi:10.1111/jsap.12320. 25728289

[156] Vargas THM LH Pulz Ferro DG , et al. Galectin-3 expression correlates with post-surgical survival in canine oral melanomas. J Comp Pathol. 2019;173:49–57. doi:10.1016/j.jcpa.2019.10.003. 31812173

[157] Vascellari M Giantin M Capello K , et al. Expression of Ki67, BCL-2, and COX-2 in canine cutaneous mast cell tumors: association with grading and prognosis. Vet Pathol. 2013;50:110–121. doi:10.1177/0300985812447829. 22673539

[158] Webster JD Dennis MM Dervisis N , et al. Recommended guidelines for the conduct and evaluation of prognostic studies in veterinary oncology. Vet Pathol. 2011;48:7–18. doi:10.1177/0300985810377187. 20664014

[159] Wei BR Halsey CH Hoover SB , et al. Agreement in histological assessment of mitotic activity between microscopy and digital whole slide images informs conversion for clinical diagnosis. Acad Pathol. 2019;6:2374289519859841. doi:10.1177/2374289519859841. PMC6628521 31321298

[160] Wilcock BP Peiffer RL Jr. Morphology and behavior of primary ocular melanomas in 91 dogs. Vet Pathol. 1986;23:418–424. doi:10.1177/030098588602300411. 3750735

[161] Willcox JL Marks SL Ueda Y , et al. Clinical features and outcome of dermal squamous cell carcinoma in 193 dogs (1987-2017). Vet Comp Oncol. 2019;17:130–138. doi:10.1111/vco.12461. 30684311

[162] Wittenberns BM Thamm DH Palmer EP , et al. Canine non-angiogenic, non-myogenic splenic stromal sarcoma: a retrospective clinicopathological analysis and investigation of podoplanin as a marker of tumour histogenesis. J Comp Pathol. 2021;188:1–12. doi:10.1016/j.jcpa.2021.07.006. 34686271 PMC8542103

[163] Yale AD Priestnall SL Pittaway R , et al. Thymic epithelial tumours in 51 dogs: histopathologic and clinicopathologic findings. Vet Comp Oncol. 2022;20:50–58. doi:10.1111/vco.12705. 34036722

[164] Yamamoto S Fukushima R Hirakawa A , et al. Histopathological and immunohistochemical evaluation of malignant potential in canine aortic body tumours. J Comp Pathol. 2013;149:182–191. doi:10.1016/j.jcpa.2012.12.007. 23465289

[165] Yamazaki H Sasai H Tanaka M , et al. Assessment of biomarkers influencing treatment success on small intestinal lymphoma in dogs. Vet Comp Oncol. 2021;19:123–131. doi:10.1111/vco.12653. 32920923

[166] Zini E Nolli S Ferri F , et al. Pheochromocytoma in dogs undergoing adrenalectomy. Vet Pathol. 2019;56:358–368. doi:10.1177/0300985818819174. 30595108

