# Supplementary material for: Mitotic activity: A systematic literature review of the assessment methodology and prognostic value in canine tumors
Source: Vet Pathol. 2024 Mar 27;61(5):752–64. doi: 10.1177/03009858241239565 (PMC11370189; doi:10.1177/03009858241239565)
Supplement: sj-pdf-1-vet-10.1177_03009858241239565 – Supplemental material for Mitotic activity: A systematic literature review of the assessment methodology and prognostic value in canine tumors [file sj-pdf-1-vet-10.1177_03009858241239565.pdf]

Supplemental Materials

Mitotic activity: a systematic literature review of the assessment methodology and prognostic value in canine tumors

Christof A. Bertram, Taryn A. Donovan, Alexander Bartel

Supplemental Table S1. Criteria applied for the risk of bias (RoB) evaluation according to a previous system established for a similar systematic review on feline tumors.[1]

Quality criteria	RoB evaluation			
	⊕ Low	◯ Moderate	⊖ High	⊖ unknown
**Study population** (domain 1):	Cumulative evaluation of each criterion within domain 1			
Sample size (smallest event / total)	At least 15 cases per outcome event	Between 7 – 14 cases per outcome event	Less than 7 cases per outcome event	Number of cases per outcome event not provided
Inclusion and exclusion criteria (case definition)	Criteria clearly stated and low bias suspected (representative study population)	Criteria clearly stated and moderate bias suspected	Criteria clearly stated and high bias suspected	Criteria not stated
Patient characteristics and tumor (sub)types	Clearly described	Partially described	–	No information
**Outcome assessment** (domain 2):	Cumulative evaluation of each criterion within domain 2			
Outcome metrics for prognosis	Appropriate outcome metrics and definitions	Appropriate outcome metrics, insufficient definitions	Inappropriate outcome metric (only tumor recurrence)	–
Follow-up method (survival, disease progression)	Regular clinical follow-up until death or end of follow-up<br><br>Confirmation of cause of death by postmortem examination	Outcome extracted from medical records<br><br>Questionnaires to the submitting vet / owner	–	Method not stated
Confirmation of recurrence and metastasis	Confirmation by cytology or histology	Suspected metastasis based on diagnostic imaging<br><br>Suspected recurrence based on recurring mass in surgical location	Based on survey with unknown method of confirmation	Method not reported
Follow-up period and proportion lost to follow-up	Appropriate period for the tumor type and few cases lost to follow-up	Short period or many cases lost to follow-up	Period considered too short for the tumor type and many cases lost to follow-up<br><br>No follow-up (e.g. presence of metastasis at time of tumor removal)	Follow-up period and proportion lost to follow-up not stated
Therapeutic treatment regimes	Only one treatment regimen for all patients; only surgical treatment is preferred	Different treatments, but similar between outcome groups	Different treatments between groups	Treatment not specified
**Mitotic activity method** (domain 3):	Cumulative evaluation of each criterion within domain 3			
Description of **mitotic count** methods (not applicable for studies on the mitotic index)	Information on area size (mm²) as well as area location, (and spatial arrangement) provided	Information of area size (in mm²) provided<br>Or<br>Information of area location and spatial arrangement	Only the number of HPF specified	No information provided

Description of **mitotic index** methods (not applicable for studies on the mitotic count)	Area selection (location, spatial arrangement) clear and number of cells or area size enumerated provided	Information on number of cells or area size enumerated provided	–	–
Consistency of mitotic count / mitotic index methods	New measurements determined for the study using predefined methods<br><br>All cases assessed by the same pathologist(s)	–	Mitotic counts taken from histopathology reports (with likely variable methods between pathologists)<br><br>Cases assessed by different pathologists	–
**Data analysis** (domain 4):	Cumulative evaluation of each criterion within domain 4			
Description of statistical methods	Clear description	Lack of some information	Lack of relevant information	No description
Outcome metrics tested	Results of statistical tests for prognostic value of MC / MI reported for all outcome metrics available in the study	Results reported for most outcome metrics available in the study	Results only reported for one of many outcome metrics available in the study (suspicious for selective reporting)	–
Relevant statistical tests (discriminant ability) performed, and results reported?	ROC curve and area under the curve, event rate, sensitivity and specificity per prognostic category, Kaplan-Meier curve and log rank test, hazard ratio (with 95% CI), median survival time	Kaplan-Meier curve and log rank test, hazard ratio (with 95% CI), median survival time	Only p-values reported<br><br>Results of statistical test stated as “significant” or “not significant” (without providing p-values) is considered as a particularly high risk of bias	No statistical test conducted (individual patient data provided)
Stratification / cut-off determination	Cut-off determined from ROC curve or scatterplots or taken from previous study	Cut-off based on mean / median / tertile	–	Cut-off determination not stated
Individual patient data provided?	Yes, outcome and mitotic count provided for each patient	Number of cases per outcome and prognostic cut-off provided (true negatives, true positives, false negative, false positive available)	–	No
**Overall risk of bias**	**Cumulative evaluation of all domains**			

ROC, receiver operating characteristic; CI, confidence interval;

This risk of bias (RoB) evaluation criteria have been developed for a previous systematic review on the mitotic activity of feline tumors[1] and was applied for canine tumors in this systematic review. The decision criteria were modified from previous recommendations [2-7] with the intention to develop a transparent decision system that is applicable for this specific topic of the systematic review. However, we acknowledge that the RoB may be underestimated regarding some quality criteria as these criteria were adjusted to the common practice of previous publications in order to enable rating of low and moderate RoB for some studies. Modifications of our decision criteria may be appropriate when new studies have adhered to recommendations for conducting and reporting prognostic studies allowing a stricter judgement of the true RoB.

References

1. Bertram CA, Donovan TA, Bartel A. Mitotic activity: a systematic review of the assessment methodology and prognostic value in feline tumors. *Vet Pathol*. 2023**:** under revision.
2. Boracchi P, Roccabianca P, Avallone G, Marano G. Kaplan-Meier Curves, Cox Model, and P-Values Are Not Enough for the Prognostic Evaluation of Tumor Markers: Statistical Suggestions for a More Comprehensive Approach. *Vet Pathol*. 2021;58**:** 795-808. 10.1177/03009858211014174
3. Donovan TA, Moore FM, Bertram CA, et al. Mitotic Figures-Normal, Atypical, and Imposters: A Guide to Identification. *Vet Pathol*. 2021;58**:** 243-257. 10.1177/0300985820980049
4. Krauth D, Woodruff TJ, Bero L. Instruments for assessing risk of bias and other methodological criteria of published animal studies: a systematic review. *Environ Health Perspect*. 2013;121**:** 985-992. 10.1289/ehp.1206389
5. Meuten DJ, Moore FM, Donovan TA, et al. International Guidelines for Veterinary Tumor Pathology: A Call to Action. *Vet Pathol*. 2021;58**:** 766-794. 10.1177/03009858211013712
6. Sauerbrei W, Taube SE, McShane LM, Cavenagh MM, Altman DG. Reporting Recommendations for Tumor Marker Prognostic Studies (REMARK): An Abridged Explanation and Elaboration. *J Natl Cancer Inst*. 2018;110**:** 803-811. 10.1093/jnci/djy088
7. Webster JD, Dennis MM, Dervisis N, et al. Recommended guidelines for the conduct and evaluation of prognostic studies in veterinary oncology. *Vet Pathol*. 2011;48**:** 7-18. 10.1177/0300985810377187

Supplemental material

Supplemental Table S2. Risk of bias of the studies on the mitotic count (MC) in canine tumors based on four domains (D1-4: study population, outcome assessment, MC methods, and data analysis).

⊕, Low risk of bias; ○, moderate risk of bias; ⊖, high risk of bias

Tumor type/group	(Sub)type / location	Article	Level of quality				
			D1: Study population	D2: Outcome assessment	D3: MC method	D4: Data analysis	Overall (D1-4)
Apocrine gland anal sac adenocarcinoma	N/A	Morello et al. [79]	○	○	⊕	○	○
	N/A	Pradel et al. [88]	○	○	⊕	○	○
	N/A	Schlag et al. [100]	⊕	○	⊖	○	○
	Early stage	Skorupski et al. [111]	○	○	⊖	⊖	⊖
	With lymph node metastasis	Tanis et al. [116]	○	⊕	⊖	○	○
Glial tumors	N/A	Merickel et al. [72]	○	⊕	○	○	○
Hemangiosarcoma	Any other than skin	Ogilvie et al. [84]	○	⊕	⊖	⊖	⊖
	Nonvisceral: skin, eye, bone, muscle	Schultheiss [103]	⊖	○	⊖	⊖	⊖
	Cutaneous	Nobrega et al. [82]	⊕	⊖	⊖	⊖	⊖
	Splenic	Kim et al. [52]	⊖	○	⊖	⊖	⊖

Tumor type/group	(Sub)type / location	Article	Level of quality				
			D1: Study population	D2: Outcome assessment	D3: MC method	D4: Data analysis	Overall (D1-4)
	Splenic	Moore et al. [78]	○	○	○	⊖	⊖
	Subcutaneous & intramuscular	Shiu et al. [106]	⊖	○	⊖	⊖	⊖
	Falciform fat	Mendez et al. [71]	⊖	○	⊖	⊖	⊖
Insulinoma	N/A	Cleland et al. [23]	○	○	○	⊖	⊖
	N/A	Dunn et al. [32]	⊖	⊖	⊖	⊖	⊖
Lymphoma	Burkitt-like	Aresu et al. [3]	⊖	⊕	○	⊖	⊖
	Multicentric	Dobson et al. [28]	⊖	○	⊖	○	⊖
	Multicentric	Kiupel et al. [54]	○	○	⊖	⊖	⊖
	Indolent	Flood-Knapik et al. [38]	○	○	⊖	○	○
	Diffuse small B-cell, nodal	Hughes et al. [49]	○	○	○	⊖	⊖
	Diffuse large B-cell, nodal	Sierra Matiz et al. [107]	○	○	⊖	○	○
	Any	Valli et al. [122]	○	○	⊖	○	○
	Small intestinal	Yamazaki et al. [131]	○	○	⊖	⊖	⊖
Mammary tumors	Malignant	Canadas et al. [16]	⊕	○	⊕	⊖	⊖
	Malignant	Carvalho et al. [17] (2016)	○	○	⊖	○	○
	Malignant	Carvalho et al. [18] (2019)	○	⊕	⊖	⊖	⊖

Tumor type/group	(Sub)type / location	Article	Level of quality				
			D1: Study population	D2: Outcome assessment	D3: MC method	D4: Data analysis	Overall (D1-4)
	Malignant	Chen et al. [20]	⊕	⊕	⊖	○	○
	Malignant	Dutra et al. [33]	⊖	○	⊕	⊖	⊖
	Malignant	Liu et al. [62]	○	○	⊖	⊖	⊖
	Malignant	Ressel et al. [92]	○	○	⊖	○	○
	Malignant	Santos et al. [98] (2013)	⊕	⊕	⊖	○	○
	Carcinoma	Santos et al. [99] (2015)	○	⊕	○	⊖	⊖
	Any	Dolka et al. [29] (2018)	⊕	○	○	○	○
	Any	Dolka et al. [30] (2016)	○	○	○	⊖	○
	Any	Mainenti et al. [65]	⊕	○	⊖	○	○
	Any	Misdorp and Hart [74]	⊕	⊖	⊖	⊖	⊖
	Neuroendocrine Carcinoma	Nakagaki et al. [80]	⊖	⊖	⊖	⊖	⊖
Mast cell tumor	Cutaneous	Berlato et al. [9] (2013)	⊕	○	⊖	⊕	⊕
	Cutaneous, Patnaik grade II	Berlato et al. [8] (2018)	⊕	○	⊕	⊕	⊕
	Cutaneous	Elston et al. [36]	○	⊖	⊖	○	○
	Cutaneous	Gregorio et al. [43]	⊕	○	⊖	○	○
	Cutaneous, Patnaik grade II	Krick et al. [56]	○	⊖	⊖	○	⊖

Tumor type/group	(Sub)type / location	Article	Level of quality				
			D1: Study population	D2: Outcome assessment	D3: MC method	D4: Data analysis	Overall (D1-4)
	Cutaneous	Preziosi et al. [89]	○	○	⊖	⊖	⊖
	Cutaneous	Romansik et al. [95]	⊕	○	⊕	○	○
	Cutaneous	Skor et al. [110]	○	○	⊖	⊖	⊖
	Cutaneous	Thamm et al. [117]	⊖	⊖	⊖	○	⊖
	Cutaneous	Vascellari et al. [125]	○	○	⊖	○	○
	Cutaneous, multiple (≥3)	O'Connell et al. [83]	○	○	⊖	○	○
	Cutaneous, Kiupel high grade, stage I	Moore et al. [77]	○	○	○	○	○
	Cutaneous and subcutaneous	Horta et al. [48]	⊕	⊕	⊕	○	○
	Cutaneous and subcutaneous	Knight et al. [55]	⊕	○	⊕	○	○
	Cutaneous and subcutaneous	Thompson et al. [118] (2016)	○	○	⊖	○	○
	Cutaneous and subcutaneous, in Pugs	McNiel et al. [68]	⊖	○	⊖	⊖	⊖
	Subcutaneous	Bostock et al. [12]	⊕	○	⊖	○	○
	Subcutaneous	Gill et al. [39]	⊖	○	⊖	⊖	⊖
	Subcutaneous	Newman et al. [81]	⊖	○	⊖	⊖	⊖
	Subcutaneous	Thompson et al. [119]	⊕	○	⊖	○	○

Tumor type/group	(Sub)type / location	Article	Level of quality				
			D1: Study population	D2: Outcome assessment	D3: MC method	D4: Data analysis	Overall (D1-4)
	Subcutaneous	Thompson et al. [120]	○	○	⊕	○	○
	Skin	Donnelly et al. [31]	○	⊖	○	⊖	⊖
	Pinna	Schwab et al. [104]	○	○	⊖	⊖	⊖
	Cutaneous, subcutaneous, mucocutaneous, <12 months old	Rigas et al. [93]	⊖	⊖	⊖	⊖	⊖
	High-risk	Pecceu et al. [86]	○	○	⊖	○	⊖
	Patnaik grade 3; cutaneous, subcutaneous & mucocutaneous	Hume et al. [50]	⊖	○	⊖	○	○
	Intramuscular	Robinson et al. [94]	⊖	⊖	⊖	⊖	⊖
	Oral mucosa	Elliott et al. [35]	○	○	⊖	○	⊖
	Not reported	Van Lelyveld et al. [123]	○	○	⊖	⊕	○
Melanocytic tumors / melanoma	Cutaneous	Laprie et al. [58]	○	⊖	⊖	○	○
	Cutaneous (exclusive paw pad)	Laver et al. [60]	○	○	⊖	○	○
	Cutaneous	Silvestri et al. [108]	○	○	⊕	⊖	⊖
	Cutaneous and oral	Martinez et al. [66]	○	○	⊖	⊖	⊖
	Cutaneous and oral	Millanta et al. [73]	○	⊖	⊖	⊖	⊖
	Cutaneous and oral	Porcellato et al. [87]	⊕	○	⊕	⊕	⊕

Tumor type/group	(Sub)type / location	Article	Level of quality				
			D1: Study population	D2: Outcome assessment	D3: MC method	D4: Data analysis	Overall (D1-4)
	Skin, lip, oral	Bostock [11]	⊕	⊕	⊖	○	○
	Skin, lip, nail bed	Schultheiss [102]	○	○	⊖	⊖	⊖
	Intraocular	Giuliano et al. [41]	○	○	⊖	⊖	⊖
	Ocular (intraocular and scleral)	Wilcock and Peiffer [127]	⊖	○	⊖	⊖	⊖
	Oral	Bergin et al. [7]	⊕	○	⊖	⊕	○
	Oral	Baja et al. [5]	○	○	⊖	○	○
	Oral	Camerino et al. [15]	⊕	○	⊖	⊖	⊖
	Oral	Hahn et al. [45]	⊖	⊕	⊕	⊖	⊖
	Oral	Prouteau et al. [90]	○	○	⊕	○	○
	Oral	Ramos-Vara et al. [91]	○	⊖	⊖	⊖	⊖
	Oral	Vargas et al. [124]	⊖	○	○	⊖	⊖
	Oral	Wei et al. [126]	⊖	⊖	○	○	○
	Oral, osteo-cartilaginous differentiation	Sanchez et al. [97]	⊖	⊖	⊖	⊖	⊖
	Non-ocular	Spangler and Kass [113]	⊕	○	⊕	○	○
Esophageal sarcoma	Spirocera-induced	Pazzi et al. [85]	⊖	⊖	⊖	⊖	⊖
Osteosarcoma	Appendicular, small-breed dogs	Amsellem et al. [2]	⊖	○	⊖	⊖	⊖

Tumor type/group	(Sub)type / location	Article	Level of quality				
			D1: Study population	D2: Outcome assessment	D3: MC method	D4: Data analysis	Overall (D1-4)
	Appendicular	Guim et al. [44]	○	○	⊖	⊖	⊖
	Appendicular	Moore et al. [75]	⊕	⊕	⊖	⊖	⊖
	Appendicular	Saam et al. [96]	○	○	⊖	○	○
	Appendicular	Schott et al. [101]	⊕	○	⊕	○	○
	Mandibular	Coyle et al. [25]	○	⊕	⊖	○	○
	Surface	Cook et al. [24]	⊖	○	⊖	○	⊖
	Any	Kirpensteijn et al. [53]	○	⊕	⊖	⊖	⊖
	Any	Loukopoulos et al. [63]	⊖	○	⊖	⊖	⊖
Pheochromocytoma	N/A	Zini et al. [132]	○	⊖	○	⊖	⊖
Pulmonary neoplasia	Carcinoma	McNiel et al. [67]	○	○	⊖	○	○
	Any primary	Able et al. [1]	○	⊖	⊖	⊖	⊖
	Any primary	McPhetridge [69]	⊕	○	⊖	○	○
Renal cell carcinoma	N/A	Carvalho et al. [19]	○	○	⊕	○	○
	N/A	Edmondson et al. [34]	⊕	○	○	○	○
Salivary gland neoplasia	N/A	Hammer et al. [46]	⊖	⊖	⊖	⊖	⊖
Sarcoma, non-osteogenic	Skin	Bray et al. [14]	⊕	○	⊖	○	○

Tumor type/group	(Sub)type / location	Article	Level of quality				
			D1: Study population	D2: Outcome assessment	D3: MC method	D4: Data analysis	Overall (D1-4)
	Skin	Ettinger et al. [37]	○	○	⊖	⊖	⊖
	Skin	Heller et al. [47]	⊕	○	⊖	○	○
	Skin	Kuntz et al. [57]	○	○	⊖	○	○
	Skin (extraoral)	Simon et al. [109]	⊖	○	⊖	○	○
	(Sub)cutaneous, with recurrence	Chiti et al. [22] (2021)	⊖	⊕	○	⊖	⊖
	Subcutaneous	McSporran [70]	⊖	⊖	○	○	⊖
	Skin	Bostock and Dye [13]	○	○	⊖	○	○
	High grade (MC ≥9)	Crownshaw et al. [26]	○	○	⊖	○	○
	High grade, any location	Selting et al. [105]	○	○	⊖	○	○
	Abdominal visceral	Linden et al. [61]	○	○	⊖	○	○
	Gastrointestinal sarcomas	Del Alcazar [27]	○	○	○	○	○
	Gastrointestinal stromal tumors	Berger et al. [6]	⊖	○	⊖	⊖	⊖
	Gastrointestinal stromal tumors	Gillespie et al. [40]	⊖	○	⊖	⊖	⊖
	Hemangiopericytoma, skin	Graves et al. [42]	○	○	⊖	⊖	⊖
	Myxosarcoma, cutaneous	Iwaki et al. [51]	⊖	○	⊖	⊖	⊖
	Peripheral nerve sheath tumor, skin	Boos et al. [10]	○	⊖	⊖	⊖	⊖

Tumor type/group	(Sub)type / location	Article	Level of quality				
			D1: Study population	D2: Outcome assessment	D3: MC method	D4: Data analysis	Overall (D1-4)
	Perivascular wall tumor, cutaneous	Chiti et al. [21]	○	⊕	○	○	○
	Perivascular wall tumor, cutaneous	Stefanello et al. [115]	○	⊕	⊖	⊖	⊖
	Smooth muscle tumors, any location	Avallone et al. [4]	⊖	○	○	⊖	⊖
	Intestinal sarcomas	Maas et al. [64]	○	○	⊖	⊖	⊖
Splenic tumors (exclusive hemangiosarcoma)	Stromal sarcoma	Moore et al. [76]	⊖	○	⊖	⊖	⊖
	"Fibrohistiocytic nodules"	Spangler and Kass [114]	○	○	⊕	⊖	⊖
	Mesenchymal (non-angiomatous, non-lymphomatous)	Spangler et al. [112]	○	○	○	○	○
	Undifferentiated stromal sarcoma (non-angiomatous, non-myogenic)	Wittenberns et al. [129]	○	○	⊕	○	○
Squamous cell carcinoma	Nasal planum	Lascelles et al. [59]	⊖	⊖	⊖	⊖	⊖
	Dermal	Willcox et al. [128]	⊖	⊖	⊖	⊖	⊖
Synovial sarcoma	N/A	Vail et al. [121]	○	○	⊖	○	○
Thymic tumors	Epithelial	Yale et al. [130]	⊖	○	○	⊖	⊖

N/A, not applicable

References:

1. Able H, Wolf-Ringwall A, Rendahl A, et al. Computed tomography radiomic features hold prognostic utility for canine lung tumors: An analytical study. *PloS one*. 2021;16**:** e0256139. doi: 10.1371/journal.pone.0256139
2. Amsellem PM, Selmic LE, Wypij JM, et al. Appendicular osteosarcoma in small-breed dogs: 51 cases (1986-2011). *J Am Vet Med Assoc*. 2014;245**:** 203-210. doi: 10.2460/javma.245.2.203
3. Aresu L, Agnoli C, Nicoletti A, et al. Phenotypical Characterization and Clinical Outcome of Canine Burkitt-Like Lymphoma. *Front Vet Sci*. 2021;8**:** 647009. doi: 10.3389/fvets.2021.647009
4. Avallone G, Pellegrino V, Muscatello LV, et al. Canine smooth muscle tumors: A clinicopathological study. *Vet Pathol*. 2022;59**:** 244-255. doi: 10.1177/03009858211066862
5. Baja AJ, Kelsey KL, Rusl, er DM, Gieger TL, Nolan MW. A retrospective study of 101 dogs with oral melanoma treated with a weekly or biweekly 6 Gy × 6 radiotherapy protocol. *Vet Comp Oncol*. 2022. doi: 10.1111/vco.12815
6. Berger EP, Johannes CM, Jergens AE, et al. Retrospective evaluation of toceranib phosphate (Palladia®) use in the treatment of gastrointestinal stromal tumors of dogs. *J Vet Intern Med*. 2018;32**:** 2045-2053. doi: 10.1111/jvim.15335
7. Bergin IL, Smedley RC, Esplin DG, Spangler WL, Kiupel M. Prognostic evaluation of Ki67 threshold value in canine oral melanoma. *Vet Pathol*. 2011;48**:** 41-53. doi: 10.1177/0300985810388947
8. Berlato D, Murphy S, Laberke S, Rasotto R. Comparison of minichromosome maintenance protein 7, Ki67 and mitotic index in the prognosis of intermediate Patnaik grade cutaneous mast cell tumours in dogs. *Vet Comp Oncol*. 2018;16**:** 535-543. doi: 10.1111/vco.12412
9. Berlato D, Murphy S, Monti P, et al. Comparison of mitotic index and Ki67 index in the prognostication of canine cutaneous mast cell tumours. *Vet Comp Oncol*. 2015;13**:** 143-150. doi: 10.1111/vco.12029
10. Boos GS, Bassuino DM, Wurster F, et al. Retrospective canine skin peripheral nerve sheath tumors data with emphasis on histologic, immunohistochemical and prognostic factors. *Pesquisa Veterinaria Brasileira*. 2015;35**:** 965-974. doi: 10.1590/S0100-736X2015001200005
11. Bostock DE. Prognosis after surgical excision of canine melanomas. *Vet Pathol*. 1979;16**:** 32-40.
12. Bostock DE, Crocker J, Harris K, Smith P. Nucleolar organiser regions as indicators of post-surgical prognosis in canine spontaneous mast cell tumours. *Br J Cancer*. 1989;59**:** 915-918. doi: 10.1038/bjc.1989.193
13. Bostock DE, Dye MT. Prognosis after surgical excision of canine fibrous connective tissue sarcomas. *Vet Pathol*. 1980;17**:** 581-588. doi: 10.1177/030098588001700507
14. Bray JP, Polton GA, McSporran KD, Bridges J, Whitbread TM. Canine soft tissue sarcoma managed in first opinion practice: outcome in 350 cases. *Vet Surg*. 2014;43**:** 774-782. doi: 10.1111/j.1532-950X.2014.12185.x

15. Camerino M, Giacobino D, Manassero L, et al. Prognostic impact of bone invasion in canine oral malignant melanoma treated by surgery and anti-CSPG4 vaccination: A retrospective study on 68 cases (2010-2020). *Vet Comp Oncol*. 2022;20**:** 189-197. doi: 10.1111/vco.12761
16. Canadas A, França M, Pereira C, et al. Canine Mammary Tumors: Comparison of Classification and Grading Methods in a Survival Study. *Vet Pathol*. 2019;56**:** 208-219. doi: 10.1177/0300985818806968
17. Carvalho MI, Pires I, Prada J, Lobo L, Queiroga FL. Ki-67 and PCNA Expression in Canine Mammary Tumors and Adjacent Nonneoplastic Mammary Glands: Prognostic Impact by a Multivariate Survival Analysis. *Vet Pathol*. 2016;53**:** 1138-1146. doi: 10.1177/0300985816646429
18. Carvalho MI, Pires I, Prada J, et al. Assessing the interleukin 35 immunoexpression in malignant canine mammary tumors: Association with clinicopathological parameters and prognosis. *Anticancer Research*. 2019;39**:** 2077-2083. doi: 10.21873/anticanres.13319
19. Carvalho S, Stoll AL, Priestnall SL, et al. Retrospective evaluation of COX-2 expression, histological and clinical factors as prognostic indicators in dogs with renal cell carcinomas undergoing nephrectomy. *Vet Comp Oncol*. 2017;15**:** 1280-1294. doi: 10.1111/vco.12264
20. Chen YC, Chen YY, Liao JW, Chang SC. Expression and prognostic value of c-met in canine mammary tumours. *Vet Comp Oncol*. 2018;16**:** 670-676. doi: 10.1111/vco.12439
21. Chiti LE, Ferrari R, Boracchi P, et al. Prognostic impact of clinical, haematological, and histopathological variables in 102 canine cutaneous perivascular wall tumours. *Vet Comp Oncol*. 2021;19**:** 275-283. doi: 10.1111/vco.12673
22. Chiti LE, Ferrari R, Roccabianca P, et al. Surgical Margins in Canine Cutaneous Soft-Tissue Sarcomas: A Dichotomous Classification System Does Not Accurately Predict the Risk of Local Recurrence. *Animals (Basel)*. 2021;11. doi: 10.3390/ani11082367
23. Cleland NT, Morton J, Delisser PJ. Outcome after surgical management of canine insulinoma in 49 cases. *Vet Comp Oncol*. 2021;19**:** 428-441. doi: 10.1111/vco.12628
24. Cook MR, Lorbach J, Husb, et al. A retrospective analysis of 11 dogs with surface osteosarcoma. *Vet Comp Oncol*. 2022;20**:** 82-90. doi: 10.1111/vco.12741
25. Coyle VJ, Rassnick KM, Borst LB, et al. Biological behaviour of canine mandibular osteosarcoma. A retrospective study of 50 cases (1999-2007). *Vet Comp Oncol*. 2015;13**:** 89-97. doi: doi: 10.1111/vco.12020
26. Crownshaw AH, McEntee MC, Nolan MW, Gieger TL. Evaluation of variables associated with outcomes in 41 dogs with incompletely excised high-grade soft tissue sarcomas treated with definitive-intent radiation therapy with or without chemotherapy. *J Am Vet Med Assoc*. 2020;256**:** 783-791. doi: 10.2460/javma.256.7.783
27. Del Alcazar CM, Mahoney JA, Dittrich K, Stefanovski D, Church ME. Outcome, prognostic factors and histological characterization of canine gastrointestinal sarcomas. *Vet Comp Oncol*. 2021;19**:** 578-586. doi: 10.1111/vco.12696

28. Dobson JM, Blackwood LB, McInnes EF, et al. Prognostic variables in canine multicentric lymphosarcoma. *J Small Anim Pract*. 2001;42**:** 377-384. doi: doi: 10.1111/j.1748-5827.2001.tb02485.x
29. Dolka I, Czopowicz M, Gruk-Jurka A, Wojtkowska A, Sapierzyński R, Jurka P. Diagnostic efficacy of smear cytology and Robinson's cytological grading of canine mammary tumors with respect to histopathology, cytomorphometry, metastases and overall survival. *PloS one*. 2018;13**:** e0191595. doi: 10.1371/journal.pone.0191595
30. Dolka I, Król M, Sapierzyński R. Evaluation of apoptosis-associated protein (Bcl-2, Bax, cleaved caspase-3 and p53) expression in canine mammary tumors: An immunohistochemical and prognostic study. *Res Vet Sci*. 2016;105**:** 124-133. doi: 10.1016/j.rvsc.2016.02.004
31. Donnelly L, Mullin C, Balko J, et al. Evaluation of histological grade and histologically tumour-free margins as predictors of local recurrence in completely excised canine mast cell tumours. *Vet Comp Oncol*. 2015;13**:** 70-76. doi: 10.1111/vco.12021
32. Dunn JK, Bostock DE, Herrtage ME, Jackson KF, Walker MJ. Insulin-secreting tumours of the canine pancreas: Clinical and pathological features of 11 cases. *Journal of Small Animal Practice*. 1993;34**:** 325-331. doi: 10.1111/j.1748-5827.1993.tb02704.x
33. Dutra AP, Azevedo Júnior GM, Schmitt FC, Cassali GD. Assessment of cell proliferation and prognostic factors in canine mammary gland tumors. *Arquivo Brasileiro de Medicina Veterinária e Zootecnia*. 2008;60**:** 1403-1412.
34. Edmondson EF, Hess AM, Powers BE. Prognostic significance of histologic features in canine renal cell carcinomas: 70 nephrectomies. *Vet Pathol*. 2015;52**:** 260-268. doi: 10.1177/0300985814533803
35. Elliott JW, Cripps P, Blackwood L, Berlato D, Murphy S, Grant IA. Canine oral mucosal mast cell tumours. *Vet Comp Oncol*. 2016;14**:** 101-111. doi: 10.1111/vco.12071
36. Elston LB, Sueiro FA, Cavalcanti JN, Metze K. The importance of the mitotic index as a prognostic factor for survival of canine cutaneous mast cell tumors: a validation study. *Vet Pathol*. 2009;46**:** 362-364, author reply 364-365. doi: 10.1354/vp.46-2-362
37. Ettinger SN, Scase TJ, Oberthaler KT, et al. Association of argyrophilic nucleolar organizing regions, Ki-67, and proliferating cell nuclear antigen scores with histologic grade and survival in dogs with soft tissue sarcomas: 60 Cases (1996-2002). *Journal of the American Veterinary Medical Association*. 2006;228**:** 1053-1062. doi: 10.2460/javma.228.7.1053
38. Flood-Knapik KE, Durham AC, Gregor TP, Sánchez MD, Durney ME, Sorenmo KU. Clinical, histopathological and immunohistochemical characterization of canine indolent lymphoma. *Vet Comp Oncol*. 2013;11**:** 272-286. doi: 10.1111/j.1476-5829.2011.00317.x
39. Gill V, Leibman N, Monette S, Craft DM, Bergman PJ. Prognostic Indicators and Clinical Outcome in Dogs with Subcutaneous Mast Cell Tumors Treated with Surgery Alone: 43 Cases. *J Am Anim Hosp Assoc*. 2020;56**:** 215-225. doi: 10.5326/JAAHA-MS-6960
40. Gillespie V, Baer K, Farrelly J, Craft D, Luong R. Canine gastrointestinal stromal tumors: immunohistochemical expression of CD34 and examination of prognostic indicators including proliferation markers Ki67 and AgNOR. *Vet Pathol*. 2011;48**:** 283-291. doi: 10.1177/0300985810380397

41. Giuliano EA, Chappell R, Fischer B, Dubielzig RR. A matched observational study of canine survival with primary intraocular melanocytic neoplasia. *Veterinary Ophthalmology*. 1999;2**:** 185-190. doi: 10.1046/j.1463-5224.1999.00080.x
42. Graves GM, Bjorling DE, Mahaffey E. Canine hemangiopericytoma: 23 cases (1967-1984). *J Am Vet Med Assoc*. 1988;192**:** 99-102.
43. Gregório H, Raposo T, Queiroga FL, Pires I, Pena L, Prada J. High COX-2 expression in canine mast cell tumours is associated with proliferation, angiogenesis and decreased overall survival. *Veterinary and comparative oncology*. 2017;15**:** 1382-1392. doi: 10.1111/vco.12280
44. Guim TN, Bianchi MV, De Lorenzo C, et al. Relationship Between Clinicopathological Features and Prognosis in Appendicular Osteosarcoma in Dogs. *Journal of Comparative Pathology*. 2020;180**:** 91-99. doi: 10.1016/j.jcpa.2020.09.003
45. Hahn KA, DeNicola DB, Richardson RC, Hahn EA. Canine oral malignant melanoma: Prognostic utility of an alternative staging system. *Journal of Small Animal Practice*. 1994;35**:** 251-256.
46. Hammer A, Getzy D, Ogilvie G, Upton M, Klausner J, Kisseberth WC. Salivary gland neoplasia in the dog and cat: survival times and prognostic factors. *J Am Anim Hosp Assoc*. 2001;37**:** 478-482. doi: 10.5326/15473317-37-5-478
47. Heller DA, Stebbins ME, Reynolds TL, Hauck ML. A retrospective study of 87 cases of canine soft tissue sarcomas. *Int J Appl Res Vet Med*. 2005;3**:** 81-87.
48. Horta RS, Lavalle GE, Monteiro LN, Souza MCC, Cassali GD, Araújo RB. Assessment of Canine Mast Cell Tumor Mortality Risk Based on Clinical, Histologic, Immunohistochemical, and Molecular Features. *Vet Pathol*. 2018;55**:** 212-223. doi: 10.1177/0300985817747325
49. Hughes KL, Ehrhart EJ, Rout ED, et al. Diffuse Small B-Cell Lymphoma: A High-Grade Malignancy. *Vet Pathol*. 2021;58**:** 912-922. doi: 10.1177/0300985820985221
50. Hume CT, Kiupel M, Rigatti L, Shofer FS, Skorupski KA, Sorenmo KU. Outcomes of dogs with grade 3 mast cell tumors: 43 cases (1997-2007). *Journal of the American Animal Hospital Association*. 2011;47**:** 37-44. doi: 10.5326/JAAHA-MS-5557
51. Iwaki Y, Lindley S, Smith A, Curran KM, Looper J. Canine myxosarcomas, a retrospective analysis of 32 dogs (2003-2018). *BMC Veterinary Research*. 2019;15. doi: 10.1186/s12917-019-1956-z
52. Kim SE, Liptak JM, Gall TT, Monteith GJ, Woods JP. Epirubicin in the adjuvant treatment of splenic hemangiosarcoma in dogs: 59 cases (1997-2004). *J Am Vet Med Assoc*. 2007;231**:** 1550-1557. doi: 10.2460/javma.231.10.1550
53. Kirpensteijn J, Kik M, Rutteman GR, Teske E. Prognostic significance of a new histologic grading system for canine osteosarcoma. *Vet Pathol*. 2002;39**:** 240-246. doi: 10.1354/vp.39-2-240
54. Kiupel M, Teske E, Bostock D. Prognostic factors for treated canine malignant lymphoma. *Vet Pathol*. 1999;36**:** 292-300.
55. Knight BJ, Wood GA, Foster RA, Coomber BL. Beclin-1 is a novel predictive biomarker for canine cutaneous and subcutaneous mast cell tumors. *Vet Pathol*. 2022;59**:** 46-56. doi: 10.1177/03009858211042578

56. Krick EL, Kiupel M, Durham AC, Thaiwong T, Brown DC, Sorenmo KU. Investigating associations between proliferation indices, c-kit, and lymph node stage in canine mast cell tumors. *Journal of the American Animal Hospital Association*. 2017;53**:** 258-264. doi: 10.5326/JAAHA-MS-6265
57. Kuntz CA, Dernell WS, Powers BE, Devitt C, Straw RC, Withrow SJ. Prognostic factors for surgical treatment of soft-tissue sarcomas in dogs: 75 cases (1986-1996). *J Am Vet Med Assoc*. 1997;211**:** 1147-1151.
58. Laprie C, Abadie J, Amardeilh MF, Net JL, Lagadic M, Delverdier M. MIB-1 immunoreactivity correlates with biologic behaviour in canine cutaneous melanoma. *Vet Dermatol*. 2001;12**:** 139-147. doi: 10.1046/j.1365-3164.2001.00236.x
59. Lascelles BD, Parry AT, Stidworthy MF, Dobson JM, White RA. Squamous cell carcinoma of the nasal planum in 17 dogs. *Vet Rec*. 2000;147**:** 473-476. doi: 10.1136/vr.147.17.473
60. Laver T, Feldhaeusser BR, Robat CS, et al. Post-surgical outcome and prognostic factors in canine malignant melanomas of the haired skin: 87 cases (2003-2015). *Canadian Veterinary Journal*. 2018;59**:** 981-987.
61. Linden D, Liptak JM, Vinayak A, et al. Outcomes and prognostic variables associated with primary abdominal visceral soft tissue sarcomas in dogs: A Veterinary Society of Surgical Oncology retrospective study. *Veterinary and comparative oncology*. 2019;17**:** 265-270. doi: 10.1111/vco.12456
62. Liu JL, Chang KC, Lo CC, Chu PY, Liu CH. Expression of autophagy-related protein Beclin-1 in malignant canine mammary tumors. *BMC Vet Res*. 2013;9**:** 75. doi: 10.1186/1746-6148-9-75
63. Loukopoulos P, Robinson WF. Clinicopathological relevance of tumour grading in canine osteosarcoma. *J Comp Pathol*. 2007;136**:** 65-73. doi: 10.1016/j.jcpa.2006.11.005
64. Maas CP, ter Haar G, van der Gaag I, Kirpensteijn J. Reclassification of small intestinal and cecal smooth muscle tumors in 72 dogs: clinical, histologic, and immunohistochemical evaluation. *Vet Surg*. 2007;36**:** 302-313. doi: 10.1111/j.1532-950X.2007.00271.x
65. Mainenti M, Rasotto R, Carnier P, Zappulli V. Oestrogen and progesterone receptor expression in subtypes of canine mammary tumours in intact and ovariectomised dogs. *Vet J*. 2014;202**:** 62-68. doi: 10.1016/j.tvjl.2014.06.003
66. Martínez CM, Peñafiel-Verdú C, Vilafranca M, et al. Cyclooxygenase-2 expression is related with localization, proliferation, and overall survival in canine melanocytic neoplasms. *Vet Pathol*. 2011;48**:** 1204-1211. doi: 10.1177/0300985810396517
67. McNiel EA, Ogilvie GK, Powers BE, Hutchison JM, Salman MD, Withrow SJ. Evaluation of prognostic factors for dogs with primary lung tumors: 67 cases (1985-1992). *J Am Vet Med Assoc*. 1997;211**:** 1422-1427.
68. McNiel EA, Prink AL, O'Brien TD. Evaluation of risk and clinical outcome of mast cell tumours in pug dogs. *Vet Comp Oncol*. 2006;4**:** 2-8. doi: 10.1111/j.1476-5810.2006.00085.x
69. McPhetridge JB, Scharf VF, Regier PJ, et al. Distribution of histopathologic types of primary pulmonary neoplasia in dogs and outcome of affected dogs: 340 cases (2010-2019). *J Am Vet Med Assoc*. 2021;260**:** 234-243. doi: 10.2460/javma.20.12.0698
70. McSporran KD. Histologic grade predicts recurrence for marginally excised canine subcutaneous soft tissue sarcomas. *Vet Pathol*. 2009;46**:** 928-933. doi: 10.1354/vp.08-VP-0277-M-FL

71. Mendez SE, Sykes Crumplar SE, Durham AC. Primary Hemangiosarcoma of the Falciform Fat in Seven Dogs (2007-2015). *J Am Anim Hosp Assoc*. 2020;56**:** 120-126. doi: 10.5326/jaaha-ms-6967
72. Merickel JL, Pluhar GE, Rendahl A, O'Sullivan MG. Prognostic histopathologic features of canine glial tumors. *Vet Pathol*. 2021;58**:** 945-951. doi: 10.1177/03009858211025795
73. Millanta F, Fratini F, Corazza M, Castagnaro M, Zappulli V, Poli A. Proliferation activity in oral and cutaneous canine melanocytic tumours: correlation with histological parameters, location, and clinical behaviour. *Res Vet Sci*. 2002;73**:** 45-51. doi: 10.1016/s0034-5288(02)00041-3
74. Misdorp W, Hart AA. Prognostic factors in canine mammary cancer. *J Natl Cancer Inst*. 1976;56**:** 779-786. doi: 10.1093/jnci/56.4.779
75. Moore AS, Dernell WS, Ogilvie GK, et al. Doxorubicin and BAY 12-9566 for the treatment of osteosarcoma in dogs: a randomized, double-blind, placebo-controlled study. *J Vet Intern Med*. 2007;21**:** 783-790. doi: 10.1892/0891-6640(2007)21[783:dabftt]2.0.co;2
76. Moore AS, Frimberger AE, Sullivan N, Moore PF. Histologic and immunohistochemical review of splenic fibrohistiocytic nodules in dogs. *J Vet Intern Med*. 2012;26**:** 1164-1168. doi: 10.1111/j.1939-1676.2012.00986.x
77. Moore AS, Frimberger AE, Taylor D, Sullivan N. Retrospective outcome evaluation for dogs with surgically excised, solitary Kiupel high-grade, cutaneous mast cell tumours. *Vet Comp Oncol*. 2020;18**:** 402-408. doi: 10.1111/vco.12565
78. Moore AS, Rassnick KM, Frimberger AE. Evaluation of clinical and histologic factors associated with survival time in dogs with stage II splenic hemangiosarcoma treated by splenectomy and adjuvant chemotherapy: 30 cases (2011-2014). *J Am Vet Med Assoc*. 2017;251**:** 559-565. doi: 10.2460/javma.251.5.559
79. Morello EM, Cino M, Giacobino D, et al. Prognostic value of ki67 and other clinical and histopathological factors in canine apocrine gland anal sac adenocarcinoma. *Animals*. 2021;11. doi: 10.3390/ani11061649
80. Nakagaki KYR, Nunes MM, Garcia APV, De Brot M, Cassali GD. Neuroendocrine Carcinomas of the Canine Mammary Gland: Histopathological and Immunohistochemical Characteristics. *Front Vet Sci*. 2020;7**:** 621714. doi: 10.3389/fvets.2020.621714
81. Newman SJ, Mrkonjich L, Walker KK, Rohrbach BW. Canine subcutaneous mast cell tumour: diagnosis and prognosis. *J Comp Pathol*. 2007;136**:** 231-239. doi: 10.1016/j.jcpa.2007.02.003
82. Nóbrega DF, Sehaber VF, Madureira R, Bracarense APFRL. Canine Cutaneous Haemangiosarcoma: Biomarkers and Survival. *Journal of Comparative Pathology*. 2019;166**:** 87-96. doi: 10.1016/j.jcpa.2018.10.181
83. O'Connell K, Thomson M. Evaluation of prognostic indicators in dogs with multiple, simultaneously occurring cutaneous mast cell tumours: 63 cases. *Vet Comp Oncol*. 2013;11**:** 51-62. doi: 10.1111/j.1476-5829.2011.00301.x
84. Ogilvie GK, Powers BE, Mallinckrodt CH, Withrow SJ. Surgery and doxorubicin in dogs with hemangiosarcoma. *J Vet Intern Med*. 1996;10**:** 379-384. doi: 10.1111/j.1939-1676.1996.tb02085.x
85. Pazzi P, Kavkovsky A, Shipov A, Segev G, Dvir E. Spirocerca lupi induced oesophageal neoplasia: Predictors of surgical outcome. *Vet Parasitol*. 2018;250**:** 71-77. doi: 10.1016/j.vetpar.2017.11.013

86. Pecceu E, Serra Varela JC, Handel I, Piccinelli C, Milne E, Lawrence J. Ultrasound is a poor predictor of early or overt liver or spleen metastasis in dogs with high-risk mast cell tumours. *Vet Comp Oncol*. 2020;18**:** 389-401. doi: 10.1111/vco.12563
87. Porcellato I, Brachelente C, Cappelli K, et al. FoxP3, CTLA-4, and IDO in Canine Melanocytic Tumors. *Vet Pathol*. 2021;58**:** 42-52. doi: 10.1177/0300985820960131
88. Pradel J, Berlato D, Dobromylskyj M, Rasotto R. Prognostic significance of histopathology in canine anal sac gland adenocarcinomas: Preliminary results in a retrospective study of 39 cases. *Vet Comp Oncol*. 2018;16**:** 518-528. doi: 10.1111/vco.12410
89. Preziosi R, Sarli G, Paltrinieri M. Prognostic value of intratumoral vessel density in cutaneous mast cell tumors of the dog. *J Comp Pathol*. 2004;130**:** 143-151. doi: 10.1016/j.jcpa.2003.10.003
90. Prouteau A, Chocteau F, de Brito C, et al. Prognostic value of somatic focal amplifications on chromosome 30 in canine oral melanoma. *Vet Comp Oncol*. 2020;18**:** 214-223. doi: 10.1111/vco.12536
91. Ramos-Vara JA, Beissenherz ME, Miller MA, et al. Retrospective study of 338 canine oral melanomas with clinical, histologic, and immunohistochemical review of 129 cases. *Vet Pathol*. 2000;37**:** 597-608. doi: 10.1354/vp.37-6-597
92. Ressel L, Puleio R, Loria GR, et al. HER-2 expression in canine morphologically normal, hyperplastic and neoplastic mammary tissues and its correlation with the clinical outcome. *Res Vet Sci*. 2013;94**:** 299-305. doi: 10.1016/j.rvsc.2012.09.016
93. Rigas K, Biasoli D, Polton G, et al. Mast cell tumours in dogs less than 12 months of age: a multi-institutional retrospective study. *J Small Anim Pract*. 2020;61**:** 449-457. doi: 10.1111/jsap.13181
94. Robinson WP, Elliott J, Baines SJ, Owen L, Shales CJ. Intramuscular mast cell tumors in 7 dogs. *Can Vet J*. 2017;58**:** 931-935.
95. Romansik EM, Reilly CM, Kass PH, Moore PF, London CA. Mitotic index is predictive for survival for canine cutaneous mast cell tumors. *Vet Pathol*. 2007;44**:** 335-341. doi: 10.1354/vp.44-3-335
96. Saam DE, Liptak JM, Stalker MJ, Chun R. Predictors of outcome in dogs treated with adjuvant carboplatin for appendicular osteosarcoma: 65 cases (1996-2006). *J Am Vet Med Assoc*. 2011;238**:** 195-206. doi: 10.2460/javma.238.2.195
97. Sánchez J, Ramirez GA, Buendia AJ, et al. Immunohistochemical characterization and evaluation of prognostic factors in canine oral melanomas with osteocartilaginous differentiation. *Vet Pathol*. 2007;44**:** 676-682. doi: 10.1354/vp.44-5-676
98. Santos AA, Lopes CC, Ribeiro JR, et al. Identification of prognostic factors in canine mammary malignant tumours: a multivariable survival study. *BMC Vet Res*. 2013;9**:** 1. doi: 10.1186/1746-6148-9-1
99. Santos M, Correia-Gomes C, Marcos R, et al. Value of the Nottingham Histological Grading Parameters and Nottingham Prognostic Index in Canine Mammary Carcinoma. *Anticancer Res*. 2015;35**:** 4219-4227.
100. Schlag AN, Johnson T, Vinayak A, Kuvaldina A, Skinner OT, Wustefeld-Janssens BG. Comparison of methods to determine primary tumour size in canine apocrine gland anal sac adenocarcinoma. *J Small Anim Pract*. 2020;61**:** 185-189. doi: 10.1111/jsap.13104

101. Schott CR, Tatiersky LJ, Foster RA, Wood GA. Histologic Grade Does Not Predict Outcome in Dogs with Appendicular Osteosarcoma Receiving the Standard of Care. *Vet Pathol*. 2018;55**:** 202-211. doi: 10.1177/0300985817747329
102. Schultheiss PC. Histologic features and clinical outcomes of melanomas of lip, haired skin, and nail bed locations of dogs. *J Vet Diagn Invest*. 2006;18**:** 422-425. doi: 10.1177/104063870601800422
103. Schultheiss PC. A retrospective study of visceral and nonvisceral hemangiosarcoma and hemangiomas in domestic animals. *J Vet Diagn Invest*. 2004;16**:** 522-526. doi: 10.1177/104063870401600606
104. Schwab TM, Popovitch C, DeBiasio J, Goldschmidt M. Clinical outcome for MCTs of canine pinnae treated with surgical excision (2004-2008). *J Am Anim Hosp Assoc*. 2014;50**:** 187-191. doi: 10.5326/JAAHA-MS-6039
105. Selting KA, Powers BE, Thompson LJ, et al. Outcome of dogs with high-grade soft tissue sarcomas treated with and without adjuvant doxorubicin chemotherapy: 39 cases (1996-2004). *J Am Vet Med Assoc*. 2005;227**:** 1442-1448. doi: 10.2460/javma.2005.227.1442
106. Shiu KB, Flory AB, Anderson CL, et al. Predictors of outcome in dogs with subcutaneous or intramuscular hemangiosarcoma. *J Am Vet Med Assoc*. 2011;238**:** 472-479. doi: 10.2460/javma.238.4.472
107. Sierra Matiz OR, Santilli J, Anai LA, et al. Prognostic significance of Ki67 and its correlation with mitotic index in dogs with diffuse large B-cell lymphoma treated with 19-week CHOP-based protocol. *J Vet Diagn Invest*. 2018;30**:** 263-267. doi: 10.1177/1040638717743280
108. Silvestri S, Porcellato I, Mechelli L, Menchetti L, Rapastella S, Brachelente C. Tumor Thickness and Modified Clark Level in Canine Cutaneous Melanocytic Tumors. *Vet Pathol*. 2019;56**:** 180-188. doi: 10.1177/0300985818798094
109. Simoes JP, Schoning P, Butine M. Prognosis of canine mast cell tumors: a comparison of three methods. *Vet Pathol*. 1994;31**:** 637-647. doi: 10.1177/030098589403100602
110. Skor O, Fuchs-Baumgartinger A, Tichy A, Kleiter M, Schwendenwein I. Pretreatment leukocyte ratios and concentrations as predictors of outcome in dogs with cutaneous mast cell tumours. *Vet Comp Oncol*. 2017;15**:** 1333-1345. doi: 10.1111/vco.12274
111. Skorupski KA, Alarcón CN, de Lorimier LP, LaDouceur EEB, Rodriguez CO, Rebhun RB. Outcome and clinical, pathological, and immunohistochemical factors associated with prognosis for dogs with early-stage anal sac adenocarcinoma treated with surgery alone: 34 cases (2002-2013). *J Am Vet Med Assoc*. 2018;253**:** 84-91. doi: 10.2460/javma.253.1.84
112. Spangler WL, Culbertson MR, Kass PH. Primary mesenchymal (nonangiomatous/nonlymphomatous) neoplasms occurring in the canine spleen: anatomic classification, immunohistochemistry, and mitotic activity correlated with patient survival. *Vet Pathol*. 1994;31**:** 37-47. doi: 10.1177/030098589403100105
113. Spangler WL, Kass PH. The histologic and epidemiologic bases for prognostic considerations in canine melanocytic neoplasia. *Vet Pathol*. 2006;43**:** 136-149. doi: 10.1354/vp.43-2-136
114. Spangler WL, Kass PH. Pathologic and prognostic characteristics of splenomegaly in dogs due to fibrohistiocytic nodules: 98 cases. *Vet Pathol*. 1998;35**:** 488-498. doi: 10.1177/030098589803500603

115. Stefanello D, Avallone G, Ferrari R, Roccabianca P, Boracchi P. Canine cutaneous perivascular wall tumors at first presentation: clinical behavior and prognostic factors in 55 cases. *J Vet Intern Med*. 2011;25**:** 1398-1405. doi: 10.1111/j.1939-1676.2011.00822.x
116. Tanis JB, Simlett-Moss AB, Ossowksa M, et al. Canine anal sac gland carcinoma with regional lymph node metastases treated with sacculectomy and lymphadenectomy: Outcome and possible prognostic factors. *Vet Comp Oncol*. 2022;20**:** 276-292. doi: 10.1111/vco.12774
117. Thamm DH, Weishaar KM, Charles JB, Ehrhart EJ, 3rd. Phosphorylated KIT as a predictor of outcome in canine mast cell tumours treated with toceranib phosphate or vinblastine. *Vet Comp Oncol*. 2020;18**:** 169-175. doi: 10.1111/vco.12525
118. Thompson JJ, Morrison JA, Pearl DL, et al. Receptor Tyrosine Kinase Expression Profiles in Canine Cutaneous and Subcutaneous Mast Cell Tumors. *Vet Pathol*. 2016;53**:** 545-558. doi: 10.1177/0300985815610388
119. Thompson JJ, Pearl DL, Yager JA, Best SJ, Coomber BL, Foster RA. Canine subcutaneous mast cell tumor: characterization and prognostic indices. *Vet Pathol*. 2011;48**:** 156-168. doi: 10.1177/0300985810387446
120. Thompson JJ, Yager JA, Best SJ, et al. Canine subcutaneous mast cell tumors: cellular proliferation and KIT expression as prognostic indices. *Vet Pathol*. 2011;48**:** 169-181. doi: 10.1177/0300985810390716
121. Vail DM, Powers BE, Getzy DM, et al. Evaluation of prognostic factors for dogs with synovial sarcoma: 36 cases (1986-1991). *J Am Vet Med Assoc*. 1994;205**:** 1300-1307.
122. Valli VE, Kass PH, San Myint M, Scott F. Canine lymphomas: association of classification type, disease stage, tumor subtype, mitotic rate, and treatment with survival. *Vet Pathol*. 2013;50**:** 738-748. doi: 10.1177/0300985813478210
123. van Lelyveld S, Warland J, Miller R, et al. Comparison between Ki-67 index and mitotic index for predicting outcome in canine mast cell tumours. *J Small Anim Pract*. 2015;56**:** 312-319. doi: 10.1111/jsap.12320
124. Vargas THM, Pulz LH, Ferro DG, et al. Galectin-3 Expression Correlates with Post-surgical Survival in Canine Oral Melanomas. *J Comp Pathol*. 2019;173**:** 49-57. doi: 10.1016/j.jcpa.2019.10.003
125. Vascellari M, Giantin M, Capello K, et al. Expression of Ki67, BCL-2, and COX-2 in canine cutaneous mast cell tumors: association with grading and prognosis. *Vet Pathol*. 2013;50**:** 110-121. doi: 10.1177/0300985812447829
126. Wei BR, Halsey CH, Hoover SB, et al. Agreement in Histological Assessment of Mitotic Activity Between Microscopy and Digital Whole Slide Images Informs Conversion for Clinical Diagnosis. *Acad Pathol*. 2019;6**:** 2374289519859841. doi: 10.1177/2374289519859841
127. Wilcock BP, Peiffer RL, Jr. Morphology and behavior of primary ocular melanomas in 91 dogs. *Vet Pathol*. 1986;23**:** 418-424. doi: 10.1177/030098588602300411
128. Willcox JL, Marks SL, Ueda Y, Skorupski KA. Clinical features and outcome of dermal squamous cell carcinoma in 193 dogs (1987-2017). *Vet Comp Oncol*. 2019;17**:** 130-138. doi: 10.1111/vco.12461

129. Wittenberns BM, Thamm DH, Palmer EP, Regan DP. Canine Non-Angiogenic, Non-Myogenic Splenic Stromal Sarcoma: a Retrospective Clinicopathological Analysis and Investigation of Podoplanin as a Marker of Tumour Histogenesis. *J Comp Pathol*. 2021;188**:** 1-12. doi: 10.1016/j.jcpa.2021.07.006
130. Yale AD, Priestnall SL, Pittaway R, Taylor AJ. Thymic epithelial tumours in 51 dogs: Histopathologic and clinicopathologic findings. *Vet Comp Oncol*. 2022;20**:** 50-58. doi: 10.1111/vco.12705
131. Yamazaki H, Sasai H, Tanaka M, et al. Assessment of biomarkers influencing treatment success on small intestinal lymphoma in dogs. *Vet Comp Oncol*. 2021;19**:** 123-131. doi: 10.1111/vco.12653
132. Zini E, Nolli S, Ferri F, et al. Pheochromocytoma in Dogs Undergoing Adrenalectomy. *Vet Pathol*. 2019;56**:** 358-368. doi: 10.1177/0300985818819174

Supplemental material

Supplemental Table S3. Summary of the mitotic count (MC) methods applied in the evaluated articles on canine tumors.

Tumor type/group	(Sub)type / location	Article	ROI location	Area exclusion	Enumerated HPF (mag.)	FN	FOV diameter	Area size	Spatial arrangement of HPF	Other methods	MC value used
Apocrine gland anal sac adenocarcinoma	N/A	Morello et al. [85]	Hotspot	Areas with extensive necrosis and apoptotic or pyknotic nuclei	10 (400x)	–	–	2.37 mm²	Consecutive, non-overlapping	–	As counted
	N/A	Pradel et al. [95]	Hotspot	Exclude areas of necrosis and apoptotic or pyknotic nuclei	10 (40x)	–	0.55 mm	–	consecutive	–	As counted
	N/A	Schlag et al. [107]	–	–	10 (40x)	–	–	–	–	–	As recorded in histopathology report
	Early stage	Skorupski et al. [118]	–	–	10 (40x)	–	–	–	–	Consensus between 2 pathologists if MC differed by >5	Mean of 2 pathologists
	With lymph node metastasis	Tanis et al. [123]	–	–	10 (x400)	–	–	–	–	–	As recorded in histopathology report
Glial tumors	N/A	Merickel et al. [76]	–	–	10 or >10 (up to 30 or more) (40x)	–	0.625 mm	–	Non-consecutive	The number of mitotic figures, was subjectively and semiquantitatively assessed and scored on a 0 to 4 scale for the whole section	0 = none, 1 = rare, 2 = low numbers, 3 = moderate numbers, and 4 = high numbers
Hemangiosarcoma	Any other than skin	Ogilvie et al. [90]	–	–	10 (40x)	–	–	–	–	–	As counted
	Non-visceral: skin, eye, bone, muscle	Schultheiss [110]	–	–	1	–	–	–	–	–	As counted
	Cutaneous	Nobrega et al. [88]	–	–	10 (x400)	–	–	–	–	–	As counted
	Splenic	Kim et al. [55]	–	–	1 (400x)	–	–	–	–	–	As recorded in histopathology report
	Splenic	Moore et al. [84]	Hotspot	Omitting fields of necrosis & hemorrhage	10 (400x)	–	–	–	Adjacent	–	As counted
	Subcutaneous & intramuscular	Shiu et al. [113]	–	–	1	–	–	–	–	–	As recorded in histopathology report
	Falciform fat	Mendez et al. [75]	–	–	10	–	–	–	–	–	Mitotic score: 0 = MC of 5–10, 1 = MC of 11–20, 2 = MC of 21–30, and 3 = MC > 30

Tumor type/group	(Sub)type / location	Article	ROI location	Area exclusion	Enumerated HPF (mag.)	FN	FOV diameter	Area size	Spatial arrangement of HPF	Other methods	MC value used
Insulinoma	N/A	Cleland et al. [23]	–	–	10	–	–	–	–	–	As recorded in histopathology report
	N/A	Dunn et al. [33]	Random	Areas of necrosis	10 (400x)	–	–	–	–	–	As counted
Lymphoma	Burkitt-like	Aresu et al. [3]	–	–	–	–	–	2.37 mm²	–	–	As counted
	Multicentric	Dobson et al. [29]	–	–	10 (x500)	–	–	–	–	Only unequivocal mitotic figures	Mean number per 1 HPF
	Multicentric	Kiupel et al. [57]	–	–	–	–	–	–	–	–	As counted
	Indolent	Flood-Knapik et al. [40]	Representat ive	Artefacts	1 (40x)	–	–	–	–	–	As counted
	Diffuse small B-cell, nodal	Hughes et al. [52]	–	–	12 (400x)	20	–	≈2.37 mm²	Consecutive	–	Per 2.4 mm²
	Diffuse large B-cell, nodal	Sierra Matiz et al. [114]	Hotspot	–	10 (400x)	–	–	–	–	–	As counted
	Any	Valli et al. [129]	Hotspot	–	≥3 (400x)	–	–	–	–	Counts were continued until the same mitotic rate was found in at least 3 areas<br><br>In some lymphomas, presence of tangible body macrophages indicated areas of high mitotic rate	Per 1 HPF
	Small intestinal	Yamazaki et al. [138]	–	–	10	–	–	–	–	–	As recorded in histopathology report
Mammary tumors	Malignant	Canadas et al. [16]	Hotspot	Areas with necrosis and large amounts of stroma	10	–	0.55 mm	2.37 mm²	–	–	As counted (joint review by 3 pathologists)
	Malignant	Carvalho et al. [17] (2016) *	Peña et al.: Hotspot, periphery	–	Peña et al.: 10	–	Peña et al.: 0.55 mm	–	–	According to Peña et al. [93]	As counted
	Malignant	Carvalho et al. [18] (2019) *	–	–	Goldschmid t et al.: 1 or 10?	–	–	–	–	According to Goldschmidt et al. [44]	As counted
	Malignant	Chen et al. [20]	–	–	10	–	–	–	–	Graded according to Goldschmidt et al. [44]	As counted
	Malignant	Dutra et al. [34]	Periphery	–	10 (x40)	–	0.55 mm	2.39 mm²	–	–	As counted
	Malignant	Liu et al. [66]	Elston and Ellis: Periphery	–	10	–	–	–	–	According to Elston and Ellis [37]	As counted
	Malignant	Ressel et al. [99]	Elston and Ellis: Periphery	–	10	–	–	–	–	According to Elston and Ellis [37]	As counted
	Malignant	Santos et al. [105] (2013)	–	–	10	–	–	–	–	–	As counted
	Carcinoma	Santos et al. [106] (2015)	–	–	10	–	0.55 mm	–	–	–	As counted
	Any	Dolka et al. [30] (2018)	Peña et al.: Hotspot, periphery	–	10 (400x)	22	–	2.39 mm²	–	According to Peña et al. [93]	Per 1 HPF
	Any	Dolka et al. [31] (2016)	–	–	10 (40x)	–	–	2.39 mm²	–	–	Mean number (per HPF)

Tumor type/group	(Sub)type / location	Article	ROI location	Area exclusion	Enumerated HPF (mag.)	FN	FOV diameter	Area size	Spatial arrangement of HPF	Other methods	MC value used
	Any	Mainenti et al. [69]	Hotspot	–	10	–	–	–	–	–	Per 1 HPF? (cut-off values more consistent with 10 HPF)
	Any	Misdorp and Hart [79] *	–	–	–	–	–	–	–	According to Scarff RW, Torloni H. Histological grading. In Histological Typing of Breast Tumors. Geneva, World Health Organization, 1968, p 18 (not accessible by us → MC method unknown)	As counted
	Neuroendocrine Carcinoma	Nakagaki et al. [86]	–	–	10 (40x)	–	–	–	–	–	As counted
Mast cell tumor	Cutaneous	Berlato et al. [9] (2013) *	Romansik et al.: Hotspot	–	10 (x400)	–	–	–	–	According to Romansik et al. [102]	As counted
	Cutaneous	Berlato et al. [8] (2018)	Hotspot	–	10 (x400)	–	0.55 mm	–	–	–	As counted
	Cutaneous	Elston et al. [38] *	Romansik et al.: Hotspot	–	10 Romansik et al.: (400x)	–	–	–	–	According to Romansik et al. [102]	As counted
	Cutaneous	Gregorio et al. [46]	Romansik et al.: Hotspot	–	10 (x400)	–	–	–	–	According to Romansik et al. [102]	As counted
	Cutaneous	Krick et al. [60]	Hotspot	–	10 (400x)	–	–	–	–	–	As counted
	Cutaneous	Preziosi et al. [96]	–	–	5 (x40)	–	–	–	–	Toluidine blue stain	Mean number per 1 HPF
	Cutaneous	Romansik et al. [102]	Hotspot	–	10 (400x)	–	–	2.7 mm²	–	–	As counted
	Cutaneous	Skor et al. [117]	–	–	10	–	–	–	–	–	As counted
	Cutaneous	Thamm et al. [124]	–	–	10	–	–	–	–	–	As counted
	Cutaneous	Vascellari et al. [132]	–	–	10	–	–	–	–	–	As counted
	Cutaneous, multiple	O'Connell et al. [89]	–	–	10	–	–	–	–	–	As recorded in histopathology report
	Cutaneous, Kiupel high grade, stage I	Moore et al. [83]	Hotspot	–	10 (x400)	–	–	–	Adjacent	–	As counted
	Cutaneous and subcutaneous	Horta et al. [51]	Hotspot	–	10	22 mm	–	–	–	–	As counted
	Cutaneous and subcutaneous	Knight et al. [59]	Hotspot	–	10	22 mm	–	–	2.37 mm²	–	Consensus by two pathologists
	Cutaneous and subcutaneous	Thompson et al. [125] (2016) *	Kiupel et al.: Hotspot	–	10	–	–	–	–	According to Kiupel [58]	As counted
	Cutaneous and subcutaneous, in Pugs	McNiel et al. [72]	–	–	10 (x400)	–	–	–	–	–	As counted
	Subcutaneous	Bostock et al. [12]	Random	–	10 (x400)	–	–	–	–	–	Per 1 HPF
	Subcutaneous	Gill et al. [41]	Hotspot	–	10 (x400)	–	–	–	–	–	As counted
	Subcutaneous	Newman et al. [87]	–	–	1 (x40)	–	–	–	–	–	As counted

Tumor type/group	(Sub)type / location	Article	ROI location	Area exclusion	Enumerated HPF (mag.)	FN	FOV diameter	Area size	Spatial arrangement of HPF	Other methods	MC value used
	Subcutaneous	Thompson et al. [126] *	Hotspot	–	10 Romansik et al.: (400x)	–	–	–	–	According to Romansik et al. [102]	As counted
	Subcutaneous	Thompson et al. [127]	Hotspot	–	10 (40x)	–	550 µm	–	–	–	As counted
	skin	Donnelly et al. [32]	Hotspot, most cellular	–	10 (400x)	–	–	–	Consecutive	–	As counted
	Pinna	Schwab et al. [111]	–	–	–	–	–	–	–	–	As counted
	Cutaneous, subcutaneous, mucocutaneous, <12 months old	Rigas et al. [100]	Tumor-representati ve	–	10 (x400)	–	–	–	–	–	As recorded in histopathology report
	High-risk	Pecceu et al. [92]	–	–	–	–	–	–	–	–	As recorded in histopathology report
	Patnaik grade 3; cutaneous, subcutaneous & mucocutaneous	Hume et al. [53]	Hotspot	–	10	–	–	–	–	–	As counted
	intramuscular	Robinson et al. [101]	–	–	10	–	–	–	–	–	As counted
	Oral mucosa	Elliott et al. [36]	–	–	10	–	–	–	–	–	As recorded in histopathology report
	Not reported	Van Lelyveld et al. [130]	–	–	10	–	–	–	–	–	As recorded in histopathology report
Melanocytic tumors / melanoma	Cutaneous	Laprie et al. [62]	Random	Ulceration	10 (x40)	–	–	–	–	–	As counted
	Cutaneous (exclusive paw pad)	Laver et al. [64]	–	–	1 or 10	–	–	–	–	If the MC was reported per 1 HPF, it was multiplied by 10	Per 10 HPF
	Cutaneous	Silvestri et al. [115]	Hotspot	Areas of necrosis and severe inflammation	10 (x40)	22 mm	–	–	Contiguous	–	–
	Cutaneous and oral	Martinez et al. [70] *	Spangler: Hotspot	–	10 (40x)	–	–	–	Spangler: Consecutive, nonoverlapping	According to Spangler and Kass [120] Bleached HE-stained sections	As counted
	Cutaneous and oral	Millanta et al. [78]	–	–	3 (x40)	–	–	–	–	–	Average per 1 HPF
	Cutaneous and oral	Porcellato et al. [94] *	Meuten: Hotspot	–	–	–	–	–	Meuten: 2.37 mm²	According to Meuten et al. [77]	As counted
	Skin, lip, oral	Bostock [11]	Random	–	10	–	–	–	–	All tumors with pigment that obscured the nuclei were given a MC = 0	As counted
	Skin, lip, nail bed	Schultheiss [109]	–	–	1	–	–	–	–	–	As counted
	Intraocular	Giuliano et al. [43]	–	–	10	–	–	–	–	Bleached slides when needed	As counted
	Ocular (intraocular and scleral)	Wilcock and Peiffer [134]	–	–	10 (approx.. 430x)	–	–	–	–	At least one section from pigmented specimens was bleached	As counted
	Oral	Bergin et al. [7]	–	–	10 (400x)	–	–	–	–	–	As counted

Tumor type/group	(Sub)type / location	Article	ROI location	Area exclusion	Enumerated HPF (mag.)	FN	FOV diameter	Area size	Spatial arrangement of HPF	Other methods	MC value used
	Oral	Baja et al. [5]	–	–	10	–	–	–	–	–	As recorded in histopathology report
	Oral	Camerino et al. [15]	–	–	10	–	–	–	–	–	As counted
	Oral	Hahn et al. [48]	Random	–	20	–	450 µm	–	–	All HPF with pigment that obscured the nuclei were given a MC = 0	Per 1 HPF
	Oral	Prouteau et al. [97]	Hotspots	–	10 (x400)	–	0.55 mm	–	–	–	As counted
	Oral	Ramos-Vara et al. [98]	–	–	10	–	–	–	–	–	As counted
	Oral	Vargas et al. [131]	Hotspot	–	10	–	–	–	Consecutive, non-overlapping	Bleached slides	Average per 1 HPF
	Oral	Wei et al. [133]	Hotspot	–	10 (glass slides: 40x; WSI: adjusted for size of light microscope)	–	–	2.37 mm² (N = 9), 1.96 mm² (N = 1)	Consecutive, non-overlapping	Using glass slides or WSI (20x or 40x scans); bleached slides	As counted
	Oral, osteo-cartilaginous differentiation	Sanchez et al. [104]	–	–	10 (400x)	–	–	–	–	–	Average per 1 HPF
	Non-ocular (cutaneous and oral)	Spangler and Kass [120]	Hotspot	–	10	–	500 µm	–	Consecutive, non-overlapping	–	As counted
Esophageal sarcoma	Spirocera-induced	Pazzi et al. [91]	–	–	–	–	–	–	–	–	Low (1-4), moderate (5-10), high (>10)
Osteo-sarcoma	Appendicular, small-breed dogs	Amsellem et al. [2]	–	–	10	–	–	–	–	–	As counted
	Appendicular	Guim et al. [47]	Random	–	10 (x400)	–	–	–	–	–	As counted
	Appendicular	Moore et al. [81]	Random	Areas of necrosis and abundant matrix	10	–	–	–	–	–	As counted
	Appendicular	Saam et al. [103] *	–	–	3 (400x)	–	–	–	–	–	As counted
	Appendicular	Schott et al. [108]	Random	–	3	–	–	1 HPF = 0.237 mm²	–	–	As counted
			–	–	10	–	–		Continuous	–	As counted
	Mandibular	Coyle et al. [25]	–	–	10	–	–	–	–	–	As counted
	Surface	Cook et al. [24]	–	–	10 (400x)	–	–	–	Non-contiguous	–	As counted
	Any	Kirpensteijn et al. [56]	Random	–	3 (400x)	–	–	–	–	–	As counted
	Any	Loukopoulos et al. [67]	Random, central and peripheral areas	–	10 (x400)	–	–	–	–	–	As counted
Pheochromocytoma	N/A	Zini et al. [139]	–	–	10	–	–	2.37 mm²	–	Disagreement solved by consensus	As counted
Pulmonary neoplasia	Carcinoma	McNiel et al. [71]	–	–	10 (400x)	–	–	–	–	–	As counted
	Any primary	Able et al. [1]	–	–	–	–	–	–	–	–	–
	Any primary	McPhetridge [73]	–	–	10	–	–	–	–	–	As recorded in histopathology report
Renal cell carcinoma	N/A	Carvalho et al. [19]	Hotspot	Hemorrhage, necrosis	10 (x400)	–	–	2.37 mm²	–	Difference (between 3 pathologists) were	As counted

Tumor type/group	(Sub)type / location	Article	ROI location	Area exclusion	Enumerated HPF (mag.)	FN	FOV diameter	Area size	Spatial arrangement of HPF	Other methods	MC value used
										discussed to achieve consensus	
	N/A	Edmondson et al. [35]	Hotspot	–	10 (400x)	–	–	–	consecutive	Differences (between 2 pathologists) were discussed jointly	As counted
Salivary gland neoplasia	N/A	Hammer et al. [49]	–	–	1 (40x)	–	–	–	–	–	As counted
Sarcomas, non-osteogenic	Skin	Bray et al. [14] *	Dennis et al: Hotspot, high cellularity	Dennis et al: Foci of necrosis, hypocellular areas, and zones of ulceration should	10 (400x)	–	–	–	Dennis et al: contiguous	According to Dennis et al. [28]	As counted
	Skin	Ettinger et al. [39]	–	–	10	–	–	–	–	–	As counted
	Skin	Heller et al. [50]	–	–	10 (400x)	–	–	–	–	–	As recorded in histopathology report or counted if MC not available in pathology report
	Skin	Kuntz et al. [61]	–	–	10 (400x)	–	–	–	–	–	As counted
	Skin (extraoral)	Simon et al. [116]	–	–	10	–	–	–	–	–	As counted
	(Sub)cutaneous, with recurrence	Chiti et al. [22] (2021)	–	–	10	–	–	2.37 mm²	–	–	As counted
	Subcutaneous	McSporran [74]	Most cellular	–	10 (400x)	–	–	–	Contiguous	–	As counted
	Skin	Bostock and Dye [13]	Random	Necrotic area	10 (400x)	–	–	–	–	–	As counted
	High grade (MC ≥9)	Crownshaw et al. [26]	–	–	10	–	–	–	–	–	As recorded in histopathology report
	High grade, any location	Selting et al. [112]	–	–	10 (400x)	–	–	–	–	–	As counted
	Abdominal visceral	Linden et al. [65]	–	–	10	–	–	–	–	–	As recorded in histopathology report
	Gastrointestinal sarcomas	Del Alcazar [27]	–	–	–	–	–	2.37 mm²	–	–	–
	Gastrointestinal stromal tumors	Berger et al. [6]	–	–	10	–	–	–	–	–	As counted or recorded in histopathology report?
	Gastrointestinal stromal tumors	Gillespie et al. [42]	–	–	50 (400x)	–	–	–	–	–	As counted
	Hemangiopericytoma, skin	Graves et al. [45]	Random	Necrotic area	10 (400x)	–	–	–	–	–	As counted
	Myxosarcoma, cutaneous	Iwaki et al. [54]	–	–	10	–	–	–	–	–	As recorded in histopathology report
	Peripheral nerve sheath tumor, skin	Boos et al. [10]	–	–	1? (40x)	–	–	–	–	–	Per 1 HPF

Tumor type/group	(Sub)type / location	Article	ROI location	Area exclusion	Enumerated HPF (mag.)	FN	FOV diameter	Area size	Spatial arrangement of HPF	Other methods	MC value used
	Perivascular wall tumor, cutaneous	Chiti et al. [21]	–	–	10	–	–	2.37 mm²	–	–	As recorded in histopathology report
	Perivascular wall tumor, cutaneous	Stefanello et al. [122]	–	–	1?	–	–	–	–	–	Per 1 HPF
	Smooth muscle tumors, any location	Avallone et al. [4]	–	–	10	–	–	2.37 mm²	–	–	As counted
	Intestinal sarcomas	Maas et al. [68]	–	–	10 (x40)	–	–	–	–	–	As counted
Splenic tumors (exclusive hemangiosarcoma)	Stromal sarcoma	Moore et al. [82]	–	–	10 (400x)	–	–	–	–	–	As counted
	“Fibrohistiocytic nodules”	Spangler and Kass [121]	Hotspot	–	10 (40x)	–	500 µm	–	Non-overlapping	–	As counted
	Mesenchymal (non-angiomatous, non-lymphomatous)	Spangler et al. [119]	Random	–	10	NA	500 µm	–	–	–	As counted
	Stromal sarcoma (non-angiomatous, non-myogenic)	Wittenberns et al. [136]	Hotspot	–	10 (x400)	–	–	2.59 mm²	–	–	Standardized to 2.37 mm²
Squamous cell carcinoma	Nasal planum	Lascelles et al. [63]	Random	Not viable area	10 (400x)	–	–	–	–	–	Mean per 1 HPF
	Dermal	Willcox et al. [135]	–	–	1-10 (400x)	–	–	–	–	If the MC was reported per 1 HPF, calculation was based on highest estimate provided	Per 10 HPF (reported or calculated from histopathology report)
Synovial sarcoma	N/A	Vail et al. [128]	–	–	10 (400x)	–	–	–	–	–	As counted
Thymic tumors	Epithelial	Yale et al. [137]	–	–	10	–	–	2.37 mm³	–	–	Values averaged from two observers

ROI, region of interest; HPF, high-power fields; mag., magnification; FN, field number of the microscope; FOV, field of view; N/A, not applicable; –, not available; hotspot, tumor location with high mitotic density/activity, WSI, whole slide image

* Eleven studies reported that their MC method was done according to a previous article.[9,14,17,18,38,70,80,94,103,125,126] Of these, seven did report some information (mostly the number of HPFs enumerated) and four did not report any information on the MC method in their own manuscript (besides the citation). For this table and data analysis, we used the information provided in the cited articles. In two studies the MC methods were not clear, as one cited article included two MC methods and the other cited article was not accessible;[18,79] thus the information was not available for our analysis.

Summary of the table

The **selected region of interest (ROI)** within the tumor section was specified in 54/132 articles (41%) as following: hotspot (region with highest mitotic density; N = 31/54, 57%), hotspot and highly cellular (N = 2/54, 4%), hotspot and tumor periphery (N = 1/54, 2%), random (N = 14/54, 26%), periphery (N = 3/54, 6%), highly cellular (N = 1/54, 2%), and tumor representative (N = 2/54, 4%). The ROI selection method was specified in 35/77 articles (45%) published before the recommendation by Meuten et al.[77] and 19/55 articles (35%) published after that recommendation. If the journal of the publication had a pathology focus, a higher number of articles described the ROI selected (68% vs. 29%). Thirteen studies (10%) described specific areas that were excluded from the ROI including abundant necrosis (N = 12), ulceration (N = 2), hemorrhage (N = 2), matrix (N = 2), inflammation (N = 1), and low cellularity (N = 1).

The **number of HPF enumerated** was provided in 122 articles (92%) with 10 HPF being most common (N = 101/122, 83%) followed by 1 HPF (N = 9/122, 7%); 3 HPFs (N = 3/122, 2%); 1 or 10 HPF (N = 2/122, 2%); and one study each with 5 HPFs, 12 HPFs (equivalent to 2.37 mm²), 20 HPFs, 50 HPFs, ≥3 HPFs, 10 or >10 HPFs, and comparing 3 and 10. The magnification of the HPFs was indicated as 400x in 68/122 studies (56%) and as 430x and 500x in one study each (not provided in 52/122 studies; 43%). The two studies with 1 or 10 HPFs, took the MC values from pathology reports and multiplied the MCs per 1 HPF by 10.[64,135] Besides these two studies, ten additional studies used a different number of HPF for the MC than were enumerated; all of which reduced the area to 1 HPF. The 10 studies that did not provide the number of HPFs enumerated included the 6 studies without any described methods, one

study that cited a paper which described two methods (1 and 10 HPFs),[18] and three studies that reported the area size in mm² (see below).

The **actual tumor area (in mm²)** was stated (N = 19) or could be calculated [77] from the field number (N = 2) or field of view diameter (N = 9) of the light microscope in 30/132 studies (23%). The following tumor area sizes were used: 2.37 mm² (N = 20/30, 67%); 2.39 mm² (N = 3/30, 10%); 1.96 mm² (N = 3/30, 10%); and one study each with 1.59 mm², 2.59 mm² (MC was standardized to 2.37mm² by multiplication), 2.7 mm², and 3.07 mm². For one study with digital whole slide images, the area enumerated was adjusted to the area of the individual pathologists' light microscope (counts were compared between light and digital microscopy).[133] While only 10/77 (13%) of the articles published before 2017 provided the area size in mm², 20/55 (36%) studies published after 2016 (any journal) and 10/14 (71%) studies published after 2016 in journals with focus on pathology provided this information.

Spatial arrangement of the individual HPFs were specified in 16/123 articles (13%) that enumerated more than 1 HPF as following: consecutive / contiguous / adjacent (N = 8/16, 50%), consecutive and non-overlapping (N = 4/16, 25%), non-overlapping (N = 2/16, 12.5%), and non-consecutive / -contiguous (N = 2/16, 12.5%). Articles published before 2017 only stated the spatial arrangement in 6/68 instances (9%), articles published after 2016 in 10/55 instances (18%), and articles published after 2016 in journals with a pathology focus in 5/14 instances (35%).

References

1. Able H, Wolf-Ringwall A, Rendahl A, et al. Computed tomography radiomic features hold prognostic utility for canine lung tumors: An analytical study. *PloS one*. 2021;16**:** e0256139. doi: 10.1371/journal.pone.0256139
2. Amsellem PM, Selmic LE, Wypij JM, et al. Appendicular osteosarcoma in small-breed dogs: 51 cases (1986-2011). *J Am Vet Med Assoc*. 2014;245**:** 203-210. doi: 10.2460/javma.245.2.203
3. Aresu L, Agnoli C, Nicoletti A, et al. Phenotypical Characterization and Clinical Outcome of Canine Burkitt-Like Lymphoma. *Front Vet Sci*. 2021;8**:** 647009. doi: 10.3389/fvets.2021.647009
4. Avallone G, Pellegrino V, Muscatello LV, et al. Canine smooth muscle tumors: A clinicopathological study. *Vet Pathol*. 2022;59**:** 244-255. doi: 10.1177/03009858211066862
5. Baja AJ, Kelsey KL, Rusl, er DM, Gieger TL, Nolan MW. A retrospective study of 101 dogs with oral melanoma treated with a weekly or biweekly 6 Gy × 6 radiotherapy protocol. *Vet Comp Oncol*. 2022. doi: 10.1111/vco.12815
6. Berger EP, Johannes CM, Jergens AE, et al. Retrospective evaluation of toceranib phosphate (Palladia®) use in the treatment of gastrointestinal stromal tumors of dogs. *J Vet Intern Med*. 2018;32**:** 2045-2053. doi: 10.1111/jvim.15335
7. Bergin IL, Smedley RC, Esplin DG, Spangler WL, Kiupel M. Prognostic evaluation of Ki67 threshold value in canine oral melanoma. *Vet Pathol*. 2011;48**:** 41-53. doi: 10.1177/0300985810388947
8. Berlato D, Murphy S, Laberke S, Rasotto R. Comparison of minichromosome maintenance protein 7, Ki67 and mitotic index in the prognosis of intermediate Patnaik grade cutaneous mast cell tumours in dogs. *Vet Comp Oncol*. 2018;16**:** 535-543. doi: 10.1111/vco.12412
9. Berlato D, Murphy S, Monti P, et al. Comparison of mitotic index and Ki67 index in the prognostication of canine cutaneous mast cell tumours. *Vet Comp Oncol*. 2015;13**:** 143-150. doi: 10.1111/vco.12029
10. Boos GS, Bassuino DM, Wurster F, et al. Retrospective canine skin peripheral nerve sheath tumors data with emphasis on histologic, immunohistochemical and prognostic factors. *Pesquisa Veterinaria Brasileira*. 2015;35**:** 965-974. doi: 10.1590/S0100-736X2015001200005
11. Bostock DE. Prognosis after surgical excision of canine melanomas. *Vet Pathol*. 1979;16**:** 32-40.
12. Bostock DE, Crocker J, Harris K, Smith P. Nucleolar organiser regions as indicators of post-surgical prognosis in canine spontaneous mast cell tumours. *Br J Cancer*. 1989;59**:** 915-918. doi: 10.1038/bjc.1989.193
13. Bostock DE, Dye MT. Prognosis after surgical excision of canine fibrous connective tissue sarcomas. *Vet Pathol*. 1980;17**:** 581-588. doi: 10.1177/030098588001700507
14. Bray JP, Polton GA, McSporran KD, Bridges J, Whitbread TM. Canine soft tissue sarcoma managed in first opinion practice: outcome in 350 cases. *Vet Surg*. 2014;43**:** 774-782. doi: 10.1111/j.1532-950X.2014.12185.x
15. Camerino M, Giacobino D, Manassero L, et al. Prognostic impact of bone invasion in canine oral malignant melanoma treated by surgery and anti-CSPG4 vaccination: A retrospective study on 68 cases (2010-2020). *Vet Comp Oncol*. 2022;20**:** 189-197. doi: 10.1111/vco.12761
16. Canadas A, França M, Pereira C, et al. Canine Mammary Tumors: Comparison of Classification and Grading Methods in a Survival Study. *Vet Pathol*. 2019;56**:** 208-219. doi: 10.1177/0300985818806968

17. Carvalho MI, Pires I, Prada J, Lobo L, Queiroga FL. Ki-67 and PCNA Expression in Canine Mammary Tumors and Adjacent Nonneoplastic Mammary Glands: Prognostic Impact by a Multivariate Survival Analysis. *Vet Pathol*. 2016;53**:** 1138-1146. doi: 10.1177/0300985816646429
18. Carvalho MI, Pires I, Prada J, et al. Assessing the interleukin 35 immunoexpression in malignant canine mammary tumors: Association with clinicopathological parameters and prognosis. *Anticancer Research*. 2019;39**:** 2077-2083. doi: 10.21873/anticanres.13319
19. Carvalho S, Stoll AL, Priestnall SL, et al. Retrospective evaluation of COX-2 expression, histological and clinical factors as prognostic indicators in dogs with renal cell carcinomas undergoing nephrectomy. *Vet Comp Oncol*. 2017;15**:** 1280-1294. doi: 10.1111/vco.12264
20. Chen YC, Chen YY, Liao JW, Chang SC. Expression and prognostic value of c-met in canine mammary tumours. *Vet Comp Oncol*. 2018;16**:** 670-676. doi: 10.1111/vco.12439
21. Chiti LE, Ferrari R, Boracchi P, et al. Prognostic impact of clinical, haematological, and histopathological variables in 102 canine cutaneous perivascular wall tumours. *Vet Comp Oncol*. 2021;19**:** 275-283. doi: 10.1111/vco.12673
22. Chiti LE, Ferrari R, Roccabianca P, et al. Surgical Margins in Canine Cutaneous Soft-Tissue Sarcomas: A Dichotomous Classification System Does Not Accurately Predict the Risk of Local Recurrence. *Animals (Basel)*. 2021;11. doi: 10.3390/ani11082367
23. Cleland NT, Morton J, Delisser PJ. Outcome after surgical management of canine insulinoma in 49 cases. *Vet Comp Oncol*. 2021;19**:** 428-441. doi: 10.1111/vco.12628
24. Cook MR, Lorbach J, Husb, et al. A retrospective analysis of 11 dogs with surface osteosarcoma. *Vet Comp Oncol*. 2022;20**:** 82-90. doi: 10.1111/vco.12741
25. Coyle VJ, Rassnick KM, Borst LB, et al. Biological behaviour of canine mandibular osteosarcoma. A retrospective study of 50 cases (1999-2007). *Vet Comp Oncol*. 2015;13**:** 89-97. doi: doi: 10.1111/vco.12020
26. Crownshaw AH, McEntee MC, Nolan MW, Gieger TL. Evaluation of variables associated with outcomes in 41 dogs with incompletely excised high-grade soft tissue sarcomas treated with definitive-intent radiation therapy with or without chemotherapy. *J Am Vet Med Assoc*. 2020;256**:** 783-791. doi: 10.2460/javma.256.7.783
27. Del Alcazar CM, Mahoney JA, Dittrich K, Stefanovski D, Church ME. Outcome, prognostic factors and histological characterization of canine gastrointestinal sarcomas. *Vet Comp Oncol*. 2021;19**:** 578-586. doi: 10.1111/vco.12696
28. Dennis MM, McSporran KD, Bacon NJ, Schulman FY, Foster RA, Powers BE. Prognostic factors for cutaneous and subcutaneous soft tissue sarcomas in dogs. *Vet Pathol*. 2011;48**:** 73-84. 10.1177/0300985810388820
29. Dobson JM, Blackwood LB, McInnes EF, et al. Prognostic variables in canine multicentric lymphosarcoma. *J Small Anim Pract*. 2001;42**:** 377-384. doi: doi: 10.1111/j.1748-5827.2001.tb02485.x
30. Dolka I, Czopowicz M, Gruk-Jurka A, Wojtkowska A, Sapierzyński R, Jurka P. Diagnostic efficacy of smear cytology and Robinson's cytological grading of canine mammary tumors with respect to histopathology, cytomorphometry, metastases and overall survival. *PloS one*. 2018;13**:** e0191595. doi: 10.1371/journal.pone.0191595
31. Dolka I, Król M, Sapierzyński R. Evaluation of apoptosis-associated protein (Bcl-2, Bax, cleaved caspase-3 and p53) expression in canine mammary tumors: An immunohistochemical and prognostic study. *Res Vet Sci*. 2016;105**:** 124-133. doi: 10.1016/j.rvsc.2016.02.004
32. Donnelly L, Mullin C, Balko J, et al. Evaluation of histological grade and histologically tumour-free margins as predictors of local recurrence in completely excised canine mast cell tumours. *Vet Comp Oncol*. 2015;13**:** 70-76. doi: 10.1111/vco.12021

33. Dunn JK, Bostock DE, Herrtage ME, Jackson KF, Walker MJ. Insulin-secreting tumours of the canine pancreas: Clinical and pathological features of 11 cases. *Journal of Small Animal Practice*. 1993;34**:** 325-331. doi: 10.1111/j.1748-5827.1993.tb02704.x
34. Dutra AP, Azevedo Júnior GM, Schmitt FC, Cassali GD. Assessment of cell proliferation and prognostic factors in canine mammary gland tumors. *Arquivo Brasileiro de Medicina Veterinária e Zootecnia*. 2008;60**:** 1403-1412.
35. Edmondson EF, Hess AM, Powers BE. Prognostic significance of histologic features in canine renal cell carcinomas: 70 nephrectomies. *Vet Pathol*. 2015;52**:** 260-268. doi: 10.1177/0300985814533803
36. Elliott JW, Cripps P, Blackwood L, Berlato D, Murphy S, Grant IA. Canine oral mucosal mast cell tumours. *Vet Comp Oncol*. 2016;14**:** 101-111. doi: 10.1111/vco.12071
37. Elston CW, Ellis IO. Pathological prognostic factors in breast cancer. I. The value of histological grade in breast cancer: experience from a large study with long-term follow-up. *Histopathol*. 1991;19**:** 403-410. 10.1111/j.1365-2559.1991.tb00229.x
38. Elston LB, Sueiro FA, Cavalcanti JN, Metze K. The importance of the mitotic index as a prognostic factor for survival of canine cutaneous mast cell tumors: a validation study. *Vet Pathol*. 2009;46**:** 362-364, author reply 364-365. doi: 10.1354/vp.46-2-362
39. Ettinger SN, Scase TJ, Oberthaler KT, et al. Association of argyrophilic nucleolar organizing regions, Ki-67, and proliferating cell nuclear antigen scores with histologic grade and survival in dogs with soft tissue sarcomas: 60 Cases (1996-2002). *Journal of the American Veterinary Medical Association*. 2006;228**:** 1053-1062. doi: 10.2460/javma.228.7.1053
40. Flood-Knapik KE, Durham AC, Gregor TP, Sánchez MD, Durney ME, Sorenmo KU. Clinical, histopathological and immunohistochemical characterization of canine indolent lymphoma. *Vet Comp Oncol*. 2013;11**:** 272-286. doi: 10.1111/j.1476-5829.2011.00317.x
41. Gill V, Leibman N, Monette S, Craft DM, Bergman PJ. Prognostic Indicators and Clinical Outcome in Dogs with Subcutaneous Mast Cell Tumors Treated with Surgery Alone: 43 Cases. *J Am Anim Hosp Assoc*. 2020;56**:** 215-225. doi: 10.5326/JAAHA-MS-6960
42. Gillespie V, Baer K, Farrelly J, Craft D, Luong R. Canine gastrointestinal stromal tumors: immunohistochemical expression of CD34 and examination of prognostic indicators including proliferation markers Ki67 and AgNOR. *Vet Pathol*. 2011;48**:** 283-291. doi: 10.1177/0300985810380397
43. Giuliano EA, Chappell R, Fischer B, Dubielzig RR. A matched observational study of canine survival with primary intraocular melanocytic neoplasia. *Veterinary Ophthalmology*. 1999;2**:** 185-190. doi: 10.1046/j.1463-5224.1999.00080.x
44. Goldschmidt M, Peña L, Rasotto R, Zappulli V. Classification and grading of canine mammary tumors. *Vet Pathol*. 2011;48**:** 117-131. 10.1177/0300985810393258
45. Graves GM, Bjorling DE, Mahaffey E. Canine hemangiopericytoma: 23 cases (1967-1984). *J Am Vet Med Assoc*. 1988;192**:** 99-102.
46. Gregório H, Raposo T, Queiroga FL, Pires I, Pena L, Prada J. High COX-2 expression in canine mast cell tumours is associated with proliferation, angiogenesis and decreased overall survival. *Veterinary and comparative oncology*. 2017;15**:** 1382-1392. doi: 10.1111/vco.12280
47. Guim TN, Bianchi MV, De Lorenzo C, et al. Relationship Between Clinicopathological Features and Prognosis in Appendicular Osteosarcoma in Dogs. *Journal of Comparative Pathology*. 2020;180**:** 91-99. doi: 10.1016/j.jcpa.2020.09.003
48. Hahn KA, DeNicola DB, Richardson RC, Hahn EA. Canine oral malignant melanoma: Prognostic utility of an alternative staging system. *Journal of Small Animal Practice*. 1994;35**:** 251-256.

49. Hammer A, Getzy D, Ogilvie G, Upton M, Klausner J, Kisseberth WC. Salivary gland neoplasia in the dog and cat: survival times and prognostic factors. *J Am Anim Hosp Assoc*. 2001;37**:** 478-482. doi: 10.5326/15473317-37-5-478
50. Heller DA, Stebbins ME, Reynolds TL, Hauck ML. A retrospective study of 87 cases of canine soft tissue sarcomas. *Int J Appl Res Vet Med*. 2005;3**:** 81-87.
51. Horta RS, Lavalle GE, Monteiro LN, Souza MCC, Cassali GD, Araújo RB. Assessment of Canine Mast Cell Tumor Mortality Risk Based on Clinical, Histologic, Immunohistochemical, and Molecular Features. *Vet Pathol*. 2018;55**:** 212-223. doi: 10.1177/0300985817747325
52. Hughes KL, Ehrhart EJ, Rout ED, et al. Diffuse Small B-Cell Lymphoma: A High-Grade Malignancy. *Vet Pathol*. 2021;58**:** 912-922. doi: 10.1177/0300985820985221
53. Hume CT, Kiupel M, Rigatti L, Shofer FS, Skorupski KA, Sorenmo KU. Outcomes of dogs with grade 3 mast cell tumors: 43 cases (1997-2007). *Journal of the American Animal Hospital Association*. 2011;47**:** 37-44. doi: 10.5326/JAAHA-MS-5557
54. Iwaki Y, Lindley S, Smith A, Curran KM, Looper J. Canine myxosarcomas, a retrospective analysis of 32 dogs (2003-2018). *BMC Veterinary Research*. 2019;15. doi: 10.1186/s12917-019-1956-z
55. Kim SE, Liptak JM, Gall TT, Monteith GJ, Woods JP. Epirubicin in the adjuvant treatment of splenic hemangiosarcoma in dogs: 59 cases (1997-2004). *J Am Vet Med Assoc*. 2007;231**:** 1550-1557. doi: 10.2460/javma.231.10.1550
56. Kirpensteijn J, Kik M, Rutteman GR, Teske E. Prognostic significance of a new histologic grading system for canine osteosarcoma. *Vet Pathol*. 2002;39**:** 240-246. doi: 10.1354/vp.39-2-240
57. Kiupel M, Teske E, Bostock D. Prognostic factors for treated canine malignant lymphoma. *Vet Pathol*. 1999;36**:** 292-300.
58. Kiupel M, Webster JD, Bailey KL, et al. Proposal of a 2-tier histologic grading system for canine cutaneous mast cell tumors to more accurately predict biological behavior. *Vet Pathol*. 2011;48**:** 147-155. 10.1177/0300985810386469
59. Knight BJ, Wood GA, Foster RA, Coomber BL. Beclin-1 is a novel predictive biomarker for canine cutaneous and subcutaneous mast cell tumors. *Vet Pathol*. 2022;59**:** 46-56. doi: 10.1177/03009858211042578
60. Krick EL, Kiupel M, Durham AC, Thaiwong T, Brown DC, Sorenmo KU. Investigating associations between proliferation indices, c-kit, and lymph node stage in canine mast cell tumors. *Journal of the American Animal Hospital Association*. 2017;53**:** 258-264. doi: 10.5326/JAAHA-MS-6265
61. Kuntz CA, Dernell WS, Powers BE, Devitt C, Straw RC, Withrow SJ. Prognostic factors for surgical treatment of soft-tissue sarcomas in dogs: 75 cases (1986-1996). *J Am Vet Med Assoc*. 1997;211**:** 1147-1151.
62. Laprie C, Abadie J, Amardeilh MF, Net JL, Lagadic M, Delverdier M. MIB-1 immunoreactivity correlates with biologic behaviour in canine cutaneous melanoma. *Vet Dermatol*. 2001;12**:** 139-147. doi: 10.1046/j.1365-3164.2001.00236.x
63. Lascelles BD, Parry AT, Stidworthy MF, Dobson JM, White RA. Squamous cell carcinoma of the nasal planum in 17 dogs. *Vet Rec*. 2000;147**:** 473-476. doi: 10.1136/vr.147.17.473
64. Laver T, Feldhaeusser BR, Robat CS, et al. Post-surgical outcome and prognostic factors in canine malignant melanomas of the haired skin: 87 cases (2003-2015). *Canadian Veterinary Journal*. 2018;59**:** 981-987.
65. Linden D, Liptak JM, Vinayak A, et al. Outcomes and prognostic variables associated with primary abdominal visceral soft tissue sarcomas in dogs: A Veterinary Society of Surgical Oncology retrospective study. *Veterinary and comparative oncology*. 2019;17**:** 265-270. doi: 10.1111/vco.12456

66. Liu JL, Chang KC, Lo CC, Chu PY, Liu CH. Expression of autophagy-related protein Beclin-1 in malignant canine mammary tumors. *BMC Vet Res*. 2013;9**:** 75. doi: 10.1186/1746-6148-9-75
67. Loukopoulos P, Robinson WF. Clinicopathological relevance of tumour grading in canine osteosarcoma. *J Comp Pathol*. 2007;136**:** 65-73. doi: 10.1016/j.jcpa.2006.11.005
68. Maas CP, ter Haar G, van der Gaag I, Kirpensteijn J. Reclassification of small intestinal and cecal smooth muscle tumors in 72 dogs: clinical, histologic, and immunohistochemical evaluation. *Vet Surg*. 2007;36**:** 302-313. doi: 10.1111/j.1532-950X.2007.00271.x
69. Mainenti M, Rasotto R, Carnier P, Zappulli V. Oestrogen and progesterone receptor expression in subtypes of canine mammary tumours in intact and ovariectomised dogs. *Vet J*. 2014;202**:** 62-68. doi: 10.1016/j.tvjl.2014.06.003
70. Martínez CM, Peñafiel-Verdú C, Vilafranca M, et al. Cyclooxygenase-2 expression is related with localization, proliferation, and overall survival in canine melanocytic neoplasms. *Vet Pathol*. 2011;48**:** 1204-1211. doi: 10.1177/0300985810396517
71. McNiel EA, Ogilvie GK, Powers BE, Hutchison JM, Salman MD, Withrow SJ. Evaluation of prognostic factors for dogs with primary lung tumors: 67 cases (1985-1992). *J Am Vet Med Assoc*. 1997;211**:** 1422-1427.
72. McNiel EA, Prink AL, O'Brien TD. Evaluation of risk and clinical outcome of mast cell tumours in pug dogs. *Vet Comp Oncol*. 2006;4**:** 2-8. doi: 10.1111/j.1476-5810.2006.00085.x
73. McPhetridge JB, Scharf VF, Regier PJ, et al. Distribution of histopathologic types of primary pulmonary neoplasia in dogs and outcome of affected dogs: 340 cases (2010-2019). *J Am Vet Med Assoc*. 2021;260**:** 234-243. doi: 10.2460/javma.20.12.0698
74. McSporran KD. Histologic grade predicts recurrence for marginally excised canine subcutaneous soft tissue sarcomas. *Vet Pathol*. 2009;46**:** 928-933. doi: 10.1354/vp.08-VP-0277-M-FL
75. Mendez SE, Sykes Crumplar SE, Durham AC. Primary Hemangiosarcoma of the Falciform Fat in Seven Dogs (2007-2015). *J Am Anim Hosp Assoc*. 2020;56**:** 120-126. doi: 10.5326/jaaha-ms-6967
76. Merickel JL, Pluhar GE, Rendahl A, O'Sullivan MG. Prognostic histopathologic features of canine glial tumors. *Vet Pathol*. 2021;58**:** 945-951. doi: 10.1177/03009858211025795
77. Meuten DJ, Moore FM, George JW. Mitotic Count and the Field of View Area: Time to Standardize. *Vet Pathol*. 2016;53**:** 7-9. 10.1177/0300985815593349
78. Millanta F, Fratini F, Corazza M, Castagnaro M, Zappulli V, Poli A. Proliferation activity in oral and cutaneous canine melanocytic tumours: correlation with histological parameters, location, and clinical behaviour. *Res Vet Sci*. 2002;73**:** 45-51. doi: 10.1016/s0034-5288(02)00041-3
79. Misdorp W, Hart AA. Prognostic factors in canine mammary cancer. *J Natl Cancer Inst*. 1976;56**:** 779-786. doi: 10.1093/jnci/56.4.779
80. Misdorp W, Hart AA. Some prognostic and epidemiologic factors in canine osteosarcoma. *J Natl Cancer Inst*. 1979;62**:** 537-545. doi: 10.1093/jnci/62.3.537
81. Moore AS, Dernell WS, Ogilvie GK, et al. Doxorubicin and BAY 12-9566 for the treatment of osteosarcoma in dogs: a randomized, double-blind, placebo-controlled study. *J Vet Intern Med*. 2007;21**:** 783-790. doi: 10.1892/0891-6640(2007)21[783:dabftt]2.0.co;2
82. Moore AS, Frimberger AE, Sullivan N, Moore PF. Histologic and immunohistochemical review of splenic fibrohistiocytic nodules in dogs. *J Vet Intern Med*. 2012;26**:** 1164-1168. doi: 10.1111/j.1939-1676.2012.00986.x
83. Moore AS, Frimberger AE, Taylor D, Sullivan N. Retrospective outcome evaluation for dogs with surgically excised, solitary Kiupel high-grade, cutaneous mast cell tumours. *Vet Comp Oncol*. 2020;18**:** 402-408. doi: 10.1111/vco.12565

84. Moore AS, Rassnick KM, Frimberger AE. Evaluation of clinical and histologic factors associated with survival time in dogs with stage II splenic hemangiosarcoma treated by splenectomy and adjuvant chemotherapy: 30 cases (2011-2014). *J Am Vet Med Assoc*. 2017;251**:** 559-565. doi: 10.2460/javma.251.5.559
85. Morello EM, Cino M, Giacobino D, et al. Prognostic value of ki67 and other clinical and histopathological factors in canine apocrine gland anal sac adenocarcinoma. *Animals*. 2021;11. doi: 10.3390/ani11061649
86. Nakagaki KYR, Nunes MM, Garcia APV, De Brot M, Cassali GD. Neuroendocrine Carcinomas of the Canine Mammary Gland: Histopathological and Immunohistochemical Characteristics. *Front Vet Sci*. 2020;7**:** 621714. doi: 10.3389/fvets.2020.621714
87. Newman SJ, Mrkonjich L, Walker KK, Rohrbach BW. Canine subcutaneous mast cell tumour: diagnosis and prognosis. *J Comp Pathol*. 2007;136**:** 231-239. doi: 10.1016/j.jcpa.2007.02.003
88. Nóbrega DF, Sehaber VF, Madureira R, Bracarense APFRL. Canine Cutaneous Haemangiosarcoma: Biomarkers and Survival. *Journal of Comparative Pathology*. 2019;166**:** 87-96. doi: 10.1016/j.jcpa.2018.10.181
89. O'Connell K, Thomson M. Evaluation of prognostic indicators in dogs with multiple, simultaneously occurring cutaneous mast cell tumours: 63 cases. *Vet Comp Oncol*. 2013;11**:** 51-62. doi: 10.1111/j.1476-5829.2011.00301.x
90. Ogilvie GK, Powers BE, Mallinckrodt CH, Withrow SJ. Surgery and doxorubicin in dogs with hemangiosarcoma. *J Vet Intern Med*. 1996;10**:** 379-384. doi: 10.1111/j.1939-1676.1996.tb02085.x
91. Pazzi P, Kavkovsky A, Shipov A, Segev G, Dvir E. Spirocerca lupi induced oesophageal neoplasia: Predictors of surgical outcome. *Vet Parasitol*. 2018;250**:** 71-77. doi: 10.1016/j.vetpar.2017.11.013
92. Pecceu E, Serra Varela JC, Handel I, Piccinelli C, Milne E, Lawrence J. Ultrasound is a poor predictor of early or overt liver or spleen metastasis in dogs with high-risk mast cell tumours. *Vet Comp Oncol*. 2020;18**:** 389-401. doi: 10.1111/vco.12563
93. Peña L, De Andrés PJ, Clemente M, Cuesta P, Pérez-Alenza MD. Prognostic value of histological grading in noninflammatory canine mammary carcinomas in a prospective study with two-year follow-up: relationship with clinical and histological characteristics. *Vet Pathol*. 2013;50**:** 94-105. 10.1177/0300985812447830
94. Porcellato I, Brachelente C, Cappelli K, et al. FoxP3, CTLA-4, and IDO in Canine Melanocytic Tumors. *Vet Pathol*. 2021;58**:** 42-52. doi: 10.1177/0300985820960131
95. Pradel J, Berlato D, Dobromylskyj M, Rasotto R. Prognostic significance of histopathology in canine anal sac gland adenocarcinomas: Preliminary results in a retrospective study of 39 cases. *Vet Comp Oncol*. 2018;16**:** 518-528. doi: 10.1111/vco.12410
96. Preziosi R, Sarli G, Paltrinieri M. Prognostic value of intratumoral vessel density in cutaneous mast cell tumors of the dog. *J Comp Pathol*. 2004;130**:** 143-151. doi: 10.1016/j.jcpa.2003.10.003
97. Prouteau A, Chocteau F, de Brito C, et al. Prognostic value of somatic focal amplifications on chromosome 30 in canine oral melanoma. *Vet Comp Oncol*. 2020;18**:** 214-223. doi: 10.1111/vco.12536
98. Ramos-Vara JA, Beissenherz ME, Miller MA, et al. Retrospective study of 338 canine oral melanomas with clinical, histologic, and immunohistochemical review of 129 cases. *Vet Pathol*. 2000;37**:** 597-608. doi: 10.1354/vp.37-6-597

99. Ressel L, Puleio R, Loria GR, et al. HER-2 expression in canine morphologically normal, hyperplastic and neoplastic mammary tissues and its correlation with the clinical outcome. *Res Vet Sci*. 2013;94**:** 299-305. doi: 10.1016/j.rvsc.2012.09.016
100. Rigas K, Biasoli D, Polton G, et al. Mast cell tumours in dogs less than 12 months of age: a multi-institutional retrospective study. *J Small Anim Pract*. 2020;61**:** 449-457. doi: 10.1111/jsap.13181
101. Robinson WP, Elliott J, Baines SJ, Owen L, Shales CJ. Intramuscular mast cell tumors in 7 dogs. *Can Vet J*. 2017;58**:** 931-935.
102. Romansik EM, Reilly CM, Kass PH, Moore PF, London CA. Mitotic index is predictive for survival for canine cutaneous mast cell tumors. *Vet Pathol*. 2007;44**:** 335-341. doi: 10.1354/vp.44-3-335
103. Saam DE, Liptak JM, Stalker MJ, Chun R. Predictors of outcome in dogs treated with adjuvant carboplatin for appendicular osteosarcoma: 65 cases (1996-2006). *J Am Vet Med Assoc*. 2011;238**:** 195-206. doi: 10.2460/javma.238.2.195
104. Sánchez J, Ramirez GA, Buendia AJ, et al. Immunohistochemical characterization and evaluation of prognostic factors in canine oral melanomas with osteocartilaginous differentiation. *Vet Pathol*. 2007;44**:** 676-682. doi: 10.1354/vp.44-5-676
105. Santos AA, Lopes CC, Ribeiro JR, et al. Identification of prognostic factors in canine mammary malignant tumours: a multivariable survival study. *BMC Vet Res*. 2013;9**:** 1. doi: 10.1186/1746-6148-9-1
106. Santos M, Correia-Gomes C, Marcos R, et al. Value of the Nottingham Histological Grading Parameters and Nottingham Prognostic Index in Canine Mammary Carcinoma. *Anticancer Res*. 2015;35**:** 4219-4227.
107. Schlag AN, Johnson T, Vinayak A, Kuvaldina A, Skinner OT, Wustefeld-Janssens BG. Comparison of methods to determine primary tumour size in canine apocrine gland anal sac adenocarcinoma. *J Small Anim Pract*. 2020;61**:** 185-189. doi: 10.1111/jsap.13104
108. Schott CR, Tatiersky LJ, Foster RA, Wood GA. Histologic Grade Does Not Predict Outcome in Dogs with Appendicular Osteosarcoma Receiving the Standard of Care. *Vet Pathol*. 2018;55**:** 202-211. doi: 10.1177/0300985817747329
109. Schultheiss PC. Histologic features and clinical outcomes of melanomas of lip, haired skin, and nail bed locations of dogs. *J Vet Diagn Invest*. 2006;18**:** 422-425. doi: 10.1177/104063870601800422
110. Schultheiss PC. A retrospective study of visceral and nonvisceral hemangiosarcoma and hemangiomas in domestic animals. *J Vet Diagn Invest*. 2004;16**:** 522-526. doi: 10.1177/104063870401600606
111. Schwab TM, Popovitch C, DeBiasio J, Goldschmidt M. Clinical outcome for MCTs of canine pinnae treated with surgical excision (2004-2008). *J Am Anim Hosp Assoc*. 2014;50**:** 187-191. doi: 10.5326/JAAHA-MS-6039
112. Selting KA, Powers BE, Thompson LJ, et al. Outcome of dogs with high-grade soft tissue sarcomas treated with and without adjuvant doxorubicin chemotherapy: 39 cases (1996-2004). *J Am Vet Med Assoc*. 2005;227**:** 1442-1448. doi: 10.2460/javma.2005.227.1442
113. Shiu KB, Flory AB, Anderson CL, et al. Predictors of outcome in dogs with subcutaneous or intramuscular hemangiosarcoma. *J Am Vet Med Assoc*. 2011;238**:** 472-479. doi: 10.2460/javma.238.4.472
114. Sierra Matiz OR, Santilli J, Anai LA, et al. Prognostic significance of Ki67 and its correlation with mitotic index in dogs with diffuse large B-cell lymphoma treated with 19-week CHOP-based protocol. *J Vet Diagn Invest*. 2018;30**:** 263-267. doi: 10.1177/1040638717743280
115. Silvestri S, Porcellato I, Mechelli L, Menchetti L, Rapastella S, Brachelente C. Tumor Thickness and Modified Clark Level in Canine Cutaneous Melanocytic Tumors. *Vet Pathol*. 2019;56**:** 180-188. doi: 10.1177/0300985818798094

116. Simoes JP, Schoning P, Butine M. Prognosis of canine mast cell tumors: a comparison of three methods. *Vet Pathol*. 1994;31**:** 637-647. doi: 10.1177/030098589403100602
117. Skor O, Fuchs-Baumgartinger A, Tichy A, Kleiter M, Schwendenwein I. Pretreatment leukocyte ratios and concentrations as predictors of outcome in dogs with cutaneous mast cell tumours. *Vet Comp Oncol*. 2017;15**:** 1333-1345. doi: 10.1111/vco.12274
118. Skorupski KA, Alarcón CN, de Lorimier LP, LaDouceur EEB, Rodriguez CO, Rebhun RB. Outcome and clinical, pathological, and immunohistochemical factors associated with prognosis for dogs with early-stage anal sac adenocarcinoma treated with surgery alone: 34 cases (2002-2013). *J Am Vet Med Assoc*. 2018;253**:** 84-91. doi: 10.2460/javma.253.1.84
119. Spangler WL, Culbertson MR, Kass PH. Primary mesenchymal (nonangiomatous/nonlymphomatous) neoplasms occurring in the canine spleen: anatomic classification, immunohistochemistry, and mitotic activity correlated with patient survival. *Vet Pathol*. 1994;31**:** 37-47. doi: 10.1177/030098589403100105
120. Spangler WL, Kass PH. The histologic and epidemiologic bases for prognostic considerations in canine melanocytic neoplasia. *Vet Pathol*. 2006;43**:** 136-149. doi: 10.1354/vp.43-2-136
121. Spangler WL, Kass PH. Pathologic and prognostic characteristics of splenomegaly in dogs due to fibrohistiocytic nodules: 98 cases. *Vet Pathol*. 1998;35**:** 488-498. doi: 10.1177/030098589803500603
122. Stefanello D, Avallone G, Ferrari R, Roccabianca P, Boracchi P. Canine cutaneous perivascular wall tumors at first presentation: clinical behavior and prognostic factors in 55 cases. *J Vet Intern Med*. 2011;25**:** 1398-1405. doi: 10.1111/j.1939-1676.2011.00822.x
123. Tanis JB, Simlett-Moss AB, Ossowksa M, et al. Canine anal sac gland carcinoma with regional lymph node metastases treated with sacculectomy and lymphadenectomy: Outcome and possible prognostic factors. *Vet Comp Oncol*. 2022;20**:** 276-292. doi: 10.1111/vco.12774
124. Thamm DH, Weishaar KM, Charles JB, Ehrhart EJ, 3rd. Phosphorylated KIT as a predictor of outcome in canine mast cell tumours treated with toceranib phosphate or vinblastine. *Vet Comp Oncol*. 2020;18**:** 169-175. doi: 10.1111/vco.12525
125. Thompson JJ, Morrison JA, Pearl DL, et al. Receptor Tyrosine Kinase Expression Profiles in Canine Cutaneous and Subcutaneous Mast Cell Tumors. *Vet Pathol*. 2016;53**:** 545-558. doi: 10.1177/0300985815610388
126. Thompson JJ, Pearl DL, Yager JA, Best SJ, Coomber BL, Foster RA. Canine subcutaneous mast cell tumor: characterization and prognostic indices. *Vet Pathol*. 2011;48**:** 156-168. doi: 10.1177/0300985810387446
127. Thompson JJ, Yager JA, Best SJ, et al. Canine subcutaneous mast cell tumors: cellular proliferation and KIT expression as prognostic indices. *Vet Pathol*. 2011;48**:** 169-181. doi: 10.1177/0300985810390716
128. Vail DM, Powers BE, Getzy DM, et al. Evaluation of prognostic factors for dogs with synovial sarcoma: 36 cases (1986-1991). *J Am Vet Med Assoc*. 1994;205**:** 1300-1307.
129. Valli VE, Kass PH, San Myint M, Scott F. Canine lymphomas: association of classification type, disease stage, tumor subtype, mitotic rate, and treatment with survival. *Vet Pathol*. 2013;50**:** 738-748. doi: 10.1177/0300985813478210
130. van Lelyveld S, Warland J, Miller R, et al. Comparison between Ki-67 index and mitotic index for predicting outcome in canine mast cell tumours. *J Small Anim Pract*. 2015;56**:** 312-319. doi: 10.1111/jsap.12320
131. Vargas THM, Pulz LH, Ferro DG, et al. Galectin-3 Expression Correlates with Post-surgical Survival in Canine Oral Melanomas. *J Comp Pathol*. 2019;173**:** 49-57. doi: 10.1016/j.jcpa.2019.10.003

132. Vascellari M, Giantin M, Capello K, et al. Expression of Ki67, BCL-2, and COX-2 in canine cutaneous mast cell tumors: association with grading and prognosis. *Vet Pathol*. 2013;50**:** 110-121. doi: 10.1177/0300985812447829
133. Wei BR, Halsey CH, Hoover SB, et al. Agreement in Histological Assessment of Mitotic Activity Between Microscopy and Digital Whole Slide Images Informs Conversion for Clinical Diagnosis. *Acad Pathol*. 2019;6**:** 2374289519859841. doi: 10.1177/2374289519859841
134. Wilcock BP, Peiffer RL, Jr. Morphology and behavior of primary ocular melanomas in 91 dogs. *Vet Pathol*. 1986;23**:** 418-424. doi: 10.1177/030098588602300411
135. Willcox JL, Marks SL, Ueda Y, Skorupski KA. Clinical features and outcome of dermal squamous cell carcinoma in 193 dogs (1987-2017). *Vet Comp Oncol*. 2019;17**:** 130-138. doi: 10.1111/vco.12461
136. Wittenberns BM, Thamm DH, Palmer EP, Regan DP. Canine Non-Angiogenic, Non-Myogenic Splenic Stromal Sarcoma: a Retrospective Clinicopathological Analysis and Investigation of Podoplanin as a Marker of Tumour Histogenesis. *J Comp Pathol*. 2021;188**:** 1-12. doi: 10.1016/j.jcpa.2021.07.006
137. Yale AD, Priestnall SL, Pittaway R, Taylor AJ. Thymic epithelial tumours in 51 dogs: Histopathologic and clinicopathologic findings. *Vet Comp Oncol*. 2022;20**:** 50-58. doi: 10.1111/vco.12705
138. Yamazaki H, Sasai H, Tanaka M, et al. Assessment of biomarkers influencing treatment success on small intestinal lymphoma in dogs. *Vet Comp Oncol*. 2021;19**:** 123-131. doi: 10.1111/vco.12653
139. Zini E, Nolli S, Ferri F, et al. Pheochromocytoma in Dogs Undergoing Adrenalectomy. *Vet Pathol*. 2019;56**:** 358-368. doi: 10.1177/0300985818819174

Supplemental material

Outcome Summary

Supplemental Table S4. Summary of the suggested association of the mitotic count (MC) with outcome (overall or tumor-specific survival, disease progression, metastasis, and recurrence) in the evaluated studies on canine tumors.

Tumor type/group	(Sub)type / location	Article	No. cases with outcome and MC	Suggested association with…				
				Survival	Disease progression	Metastasis	Tumor recurrence	Recurrence of clinical signs
Apocrine gland anal sac adenocarcinoma	N/A	Morello et al. [81]	24	No	No	–	–	–
	N/A	Pradel et al. [91]	29 (survival), 24 (progression)	No	Yes	No	–	–
	N/A	Schlag et al. [103]	107	–	–	No	–	–
	Early stage	Skorupski et al. [114]	20	No	–	No	No	–
	With lymph node metastasis	Tanis et al. [119]	48	No	No	–	–	–
Glial tumors	N/A	Merickel et al. [74]	33	Yes	–	–	–	–
Hemangiosarcoma	Any other than skin	Ogilvie et al. [86]	46	Yes	Yes	–	–	–
	Non-visceral: skin, eye, bone, muscle	Schultheiss [106]	66 (+10 feline cases)	No	–	–	–	–
	Cutaneous	Nobrega et al. [84]	60	No	–	–	–	–
	Splenic	Kim et al. [54]	40	Yes	–	–	–	–
	Splenic, stage II	Moore et al. [80]	30	Yes	–	–	–	–
	Subcutaneous & intramuscular	Shiu et al. [109]	43	No	No	–	–	–
	Falciform fat	Mendez et al. [73]	6	No	–	–	–	–
Insulinoma	N/A	Cleland et al. [23]	25	–	–	–	–	Yes
	N/A	Dunn et al. [32]	8 (survival) or 7 (recurrence)	Yes	–	–	–	ND
Lymphoma	Burkitt-like	Aresu et al. [3]	13	No	No	–	–	–

Tumor type/group	(Sub)type / location	Article	No. cases with outcome and MC	Suggested association with…				
				Survival	Disease progression	Metastasis	Tumor recurrence	Recurrence of clinical signs
	Multicentric	Dobson et al. [28]	43	–	–	–	No	–
	Multicentric	Kiupel et al. [56]	74 (survival), 48 (progression)	Yes	No	–	–	–
	Indolent	Flood-Knapik et al. [39]	73	No	–	–	–	–
	Diffuse small B-cell, nodal	Hughes et al. [51]	22 (overall survival) or 11 (tumor-specific survival)	No	–	–	–	–
	Diffuse large B-cell, nodal	Sierra Matiz et al. [110]	29	No	–	–	–	–
	Any	Valli et al. [125]	183	Yes	–	–	–	–
	Small intestinal	Yamazaki et al. [134]	36 (surgery) and 48 (chemotherapy)	No	–	–	–	–
Mammary tumors	Malignant	Canadas et al. [16]	85	No (all cases), Yes (simple, solid and intraductal carcinoma)	–	–	–	–
	Malignant	Carvalho et al. [17] (2016)	43	Yes	–	–	–	–
	Malignant	Carvalho et al. [18] (2019)	72	Yes	–	–	–	–
	Malignant	Chen et al. [20]	72	No	–	–	–	–
	Malignant	Dutra et al. [33]	17	Yes	–	–	–	–
	Malignant	Liu et al. [64]	70	Yes	–	–	–	–
	Malignant	Ressel et al. [95]	35	Yes	–	–	–	–

Tumor type/group	(Sub)type / location	Article	No. cases with outcome and MC	Suggested association with…				
				Survival	Disease progression	Metastasis	Tumor recurrence	Recurrence of clinical signs
	Malignant	Santos et al. [101] (2013)	85	No	No	–	–	–
	Carcinoma	Santos et al. [102] (2015)	59	No	No	–	–	–
	Any	Dolka et al. [29] (2018)	59 (survival), 57 (metastasis)	Yes	–	Yes	–	–
	Any	Dolka et al. [30] (2016)	40	Yes	–	–	–	–
	Any	Mainenti et al. [67]	113	Yes	–	–	–	–
	Any	Misdorp and Hart [76]	168	No	–	–	–	–
	Neuroendocrine Carcinoma	Nakagaki et al. [82]	8 (survival), 4 (lymph node metastasis)	ND	–	ND	–	–
Mast cell tumor	Cutaneous	Berlato et al. [9] (2013)	95	Yes	–	–	–	–
	Cutaneous	Berlato et al. [8] (2018)	90	Yes	–	–	–	–
	Cutaneous	Elston et al. [36]	57	Yes	–	–	–	–
	Cutaneous	Gregorio et al. [45]	43	Yes	–	–	–	–
	Cutaneous	Krick et al. [58]	64	–	–	No	–	–
	Cutaneous	Preziosi et al. [92]	32	Yes	No	–	–	–
	Cutaneous	Romansik et al. [98]	99 (survival), 148 (metastasis and recurrence)	Yes	–	Yes	No	–
	Cutaneous	Skor et al. [113]	52	Yes	Yes	–	–	–
	Cutaneous	Thamm et al. [120]	74?	Yes	Yes	–	–	–
	Cutaneous	Vascellari et al. [128]	53	Yes	–	–	–	–
	Cutaneous, multiple (≥3)	O'Connell et al. [85]	57	Yes	No	–	–	–
	Cutaneous, Kiupel high grade, stage I	Moore et al. [79]	49	Yes	–	–	–	–
	Cutaneous and subcutaneous	Horta et al. [50]	149	Yes	–	–	–	–

Tumor type/group	(Sub)type / location	Article	No. cases with outcome and MC	Suggested association with…				
				Survival	Disease progression	Metastasis	Tumor recurrence	Recurrence of clinical signs
	Cutaneous and subcutaneous	Knight et al. [57]	139	Yes	Yes	–	–	–
	Cutaneous and subcutaneous	Thompson et al. [121] (2016)	22	Yes	Yes	Yes	No	–
	Cutaneous and subcutaneous, in Pugs	McNiel et al. [70]	25	Yes	–	–	–	–
	Subcutaneous	Bostock et al. [12]	50	Yes	–	–	–	–
	Subcutaneous	Gill et al. [40]	43	No	Yes	–	–	–
	Subcutaneous	Newman et al. [83]	53	No	–	–	–	–
	Subcutaneous	Thompson et al. [122]	306	Yes	Yes	Yes	Yes	–
	Subcutaneous	Thompson et al. [123]	48 (recurrence), 24 (metastasis); cases are subset from [122]	–	–	Yes	Yes	–
	Skin	Donnelly et al. [31]	90	–	–	–	Yes	–
	Pinna	Schwab et al. [107]	28	Yes	–	–	Yes	–
	Cutaneous, subcutaneous, mucocutaneous, <12 months old	Rigas et al. [96]	16	–	ND	ND	–	–
	High-risk	Pecceu et al. [88]	69	Yes	Yes	No	–	–
	Patnaik grade 3; cutaneous, subcutaneous & mucocutaneous	Hume et al. [52]	20	Yes	Yes	–	–	–
	Intramuscular	Robinson et al. [97]	6	ND	ND	–	–	–
	Oral mucosa	Elliott et al. [35]	21	Yes	Yes	–	–	–
	Not reported	Van Lelyveld et al. [126]	162	Yes	–	–	–	–
	Cutaneous	Laprie et al. [60]	68	Yes	Yes	–	–	–

Tumor type/group	(Sub)type / location	Article	No. cases with outcome and MC	Suggested association with…				
				Survival	Disease progression	Metastasis	Tumor recurrence	Recurrence of clinical signs
Melanocytic tumors / melanoma	Cutaneous (exclusive paw pad)	Laver et al. [62]	87	Yes	Yes	–	–	–
	Cutaneous	Silvestri et al. [111]	77	Yes	No	–	–	–
	Cutaneous and oral	Martinez et al. [68]	41	Yes	–	–	–	–
	Cutaneous and oral	Millanta et al. [75]	38	Yes	–	–	–	–
	Cutaneous and oral	Porcellato et al. [90]	75	Yes	–	–	–	–
	Skin, lip, oral	Bostock [11]	85 (skin), 31 (oral)	Yes (skin), No (oral)	–	–	–	–
	Skin, lip, nail bed	Schultheiss [105]	32	No	–	–	–	–
	Intraocular	Giuliano et al. [42]	36	No	–	–	–	–
	Ocular (intraocular and scleral)	Wilcock and Peiffer [130]	61	–	–	Yes	–	–
	Oral	Bergin et al. [7]	79 (partially from [37,116])	Yes	–	–	–	–
	Oral	Baja et al. [5]	64	No	No	–	–	–
	Oral	Camerino et al. [15]	68	No	–	–	–	–
	Oral	Hahn et al. [47]	32	Yes	Yes	–	–	–
	Oral	Prouteau et al. [93]	73	Yes	–	–	–	–
	Oral	Ramos-Vara et al. [94]	40	No	–	–	–	–
	Oral	Vargas et al. [127]	27	No	–	–	–	–
	Oral	Wei et al. [129]	66	Yes	–	–	–	–
	Oral, osteo-cartilaginous differentiation	Sanchez et al. [100]	10	–	–	–	ND	–
	Non-ocular (cutaneous and oral)	Spangler and Kass [116]	384	Yes	–	–	–	–
Esophageal sarcoma	Spirocera-induced	Pazzi et al. [87]	17	No	–	–	–	–
Osteosarcoma	Appendicular, small-breed dogs	Amsellem et al. [2]	28 (analyzed in three separate groups of	No	No	–	–	–

Tumor type/group	(Sub)type / location	Article	No. cases with outcome and MC	Suggested association with…				
				Survival	Disease progression	Metastasis	Tumor recurrence	Recurrence of clinical signs
			unknown sample size)					
	Appendicular	Guim et al. [46]	22	No	–	–	–	–
	Appendicular	Moore et al. [77]	155	Yes	–	–	–	–
	Appendicular	Saam et al. [99]	35	Yes	–	–	–	–
	Appendicular	Schott et al. [104]	85	Yes (evaluator B, per 3 HPF) No (evaluator A and C, per 3 HPF; all evaluator s, per 10 HPF)	–	Yes (evaluator B, per 3 HPF) No (evaluator A and C, per 3 HPF; all evaluators, per 10 HPF)	–	–
	Mandibular	Coyle et al. [25]	43	Yes	–	Yes	–	–
	Surface	Cook et al. [24]	11	No	No	–	–	–
	Any	Kirpensteijn et al. [55]	99	–	Yes	–	–	–
	Any	Loukopoulos et al. [65]	Unknown	–	–	Yes	–	–
Pheochromocytoma	N/A	Zini et al. [135]	24	No	–	–	–	–
Pulmonary neoplasia	Carcinoma	McNiel et al. [69]	67	Yes	Yes	–	–	–
	Any primary	Able et al. [1]	65	–	Yes	–	–	–
	Any primary	McPhetridge [71]	284	Yes	–	–	–	–
Renal cell carcinoma	N/A	Carvalho et al. [19]	51	Yes	–	–	–	–
	N/A	Edmondson et al. [34]	70	Yes	–	–	–	–
Salivary gland neoplasia	N/A	Hammer et al. [48]	24	No	–	–	–	–
Sarcomas, non-osteogenic	Skin	Bray et al. [14]	350	Yes	–	–	Yes	–
	Skin	Ettinger et al. [38]	54	Yes	–	Yes	–	–
	Skin	Heller et al. [49]	87	–	–	No	No	–
	Skin	Kuntz et al. [59]	75	Yes	–	Yes	–	–
	Skin (extraoral)	Simon et al. [112]	27	Yes	–	No	Yes	–

Tumor type/group	(Sub)type / location	Article	No. cases with outcome and MC	Suggested association with…				
				Survival	Disease progression	Metastasis	Tumor recurrence	Recurrence of clinical signs
	(Sub)cutaneous, with recurrence	Chiti et al. [22] (2021)	23	ND	–	–	ND	–
	Subcutaneous	McSporran [72]	112	–	–	–	Yes	–
	Skin	Bostock and Dye [13]	160	Yes	–	Yes	Yes	–
	High grade (MC ≥9)	Crownshaw et al. [26]	41	No	Yes	–	–	–
	High grade, any location	Selting et al. [108]	39	No	No	No	No	–
	Abdominal visceral	Linden et al. [63]	31	Yes	Yes	–	–	–
	Gastrointestinal sarcomas	Del Alcazar [27]	42	Yes	–	Yes	–	–
	Gastrointestinal stromal tumors	Berger et al. [6]	22	–	Yes	–	–	–
	Gastrointestinal stromal tumors	Gillespie et al. [41]	16	ND	–	–	–	–
	Hemangiopericytoma, skin	Graves et al. [44]	16	–	–	–	No	–
	Myxosarcoma, cutaneous	Iwaki et al. [53]	29	No	–	Yes	Yes	–
	Peripheral nerve sheath tumor, skin	Boos et al. [10]	54	–	–	–	Yes	–
	Perivascular wall tumor, cutaneous	Chiti et al. [21]	102	Yes	–	–	Yes	–
	Perivascular wall tumor, cutaneous	Stefanello et al. [118]	53	–	No	–	–	–
	Smooth muscle tumors, any location	Avallone et al. [4]	25	Yes	–	Yes	–	–
	Intestinal sarcomas	Maas et al. [66]	61	No	No	–	–	–
Splenic tumors (exclusive hemangiosarcoma)	Stromal sarcoma	Moore et al. [78]	8	No	–	–	–	–
	"Fibrohisticytic nodules"	Spangler and Kass [117]	93	Yes	–	–	–	–
	Mesenchymal (non-angiomatous, non-lymphomatous)	Spangler et al. [115]	68	Yes	–	–	–	–

Tumor type/group	(Sub)type / location	Article	No. cases with outcome and MC	Suggested association with…				
				Survival	Disease progression	Metastasis	Tumor recurrence	Recurrence of clinical signs
	Stromal sarcoma (non-angiomatous, non-myogenic)	Wittenberns et al. [132]	24	Yes	–	–	–	–
Squamous cell carcinoma	Nasal planum	Lascelles et al. [61]	12	–	–	–	No	–
	Dermal	Willcox et al. [131]	93	Yes	–	–	–	–
Synovial sarcoma	N/A	Vail et al. [124]	34	Yes	Yes	–	–	–
Thymic tumors	Epithelial	Yale et al. [133]	30	No	No	No	No	–

N/A, not available; - , not available; ND, statistical association of the MC with outcome is not provided (individual patient data available); HPF, high-power field

Survival

Supplemental Table S5. Summary of the prognostic value of the mitotic count (MC) regarding survival including all-cause mortality (ACM) and tumor-related mortality (TRM).

Tumor type/group	(Sub)type / location	Article	Number of cases with outcome	ACM / TRM	IPD	Cut-off ranges	MST of low MC cases in days (d), weeks (w), or months (m)	MST of high MC cases in days (d) or weeks (w)	Kaplan-Meier curve	Log rank test	Cox proportional hazard regression model, univariable (95% confidence interval)	ROC and AUC	Other statistical tests
Apocrine gland anal sac adenocarcinoma	N/A	Morello et al. [81]	24	ACM	–	<16.5, ≥16.5	877 d	352 d	–	p = 0.053	–	–	–
	N/A	Pradel et al. [91]	29	ACM	–	<8, ≥8	610 d	610 d	–	p = 0.986	–	–	–
	N/A	Schlag et al. [103]	–	–	–	–	–	–	–	–	–	–	–
	Early stage	Skorupski et al. [114]	20	ACM	–	–	–	–	–	p = 0.56	–	–	–
	With lymph node metastasis	Tanis et al. [119]	48	TRD	–	<8, ≥8	340 d	378 d	–	p = 0.4	–	–	–
Glial tumors	N/A	Merickel et al. [74]	33	?	–	Grade 0, 1, 2, 3, 4	Grade 0: 363d<br>Grade 1: 205d<br>Combined: 293 d	Grade 2: 218d<br>Grade 3: 158d<br>Grade 4: 60 d<br>Combined: 190 d	Yes	p = 0.0421	–	–	–
Hemangio-sarcoma	Any other than skin	Ogilvie et al. [86]	46	?	–	Score 0: ≤10<br>Score 1: 11-20<br>Score 2: 21-20<br>Score 3: >30	–	–	–	All cases: p = 0.055<br>Cases with complete removal: p = 0.017	–	–	–
	Nonvisceral: skin, eye, bone, muscle	Schultheiss [106]	66 (+10 feline cases)	?	–	≤ 2, < 2	–	–	–	–	–	–	TN: 25<br>TP: 11<br>FN: 23<br>TP: 17<br>Sen: 32%<br>Spe: 60%<br>"did not correlate"
	Cutaneous	Nobrega et al. [84]	60	ACM	–	1-5, 6-11, 11-20, >20	–	–	–	–	–	–	Gray's test: p = 0.16, 0.42, 0.14
	Splenic	Kim et al. [54]	40	ACM	–	0: none<br>1: 1<br>2: 2-3<br>3: ≥4	–	–	–	–	–	–	p = 0.018<br><br>Multivariable HR = 1.45
	Splenic, stage II	Moore et al. [80]	30	ACM	–	Score 0: <11, Score 1: 11-20, Score 2: 21-30, Score 3: >30	Score 0: 292 d<br>Score 1: 212 d	Score 2: 118 d<br>Score 3: 116 d	Yes	p = 0.002	–	–	–

Tumor type/group	(Sub)type / location	Article	Number of cases with outcome	ACM / TRM	IPD	Cut-off ranges	MST of low MC cases in days (d), weeks (w), or months (m)	MST of high MC cases in days (d) or weeks (w)	Kaplan-Meier curve	Log rank test	Cox proportional hazard regression model, univariable (95% confidence interval)	ROC and AUC	Other statistical tests
	Subcutaneous & intramuscular	Shiu et al. [109]	43	ACM	–	–	–	–	–	–	HR = 1.116; p = 0.090	–	–
	Falciform fat	Mendez et al. [73]	6	ACM	Yes	score: 0 (MC = 5-10); score: >0 (MC >10)	339 d	296 d	–	p = 0.44	–	–	–
Insulinoma	N/A	Cleland et al. [23]	–	–	–	–	–	–	–	–	–	–	–
	N/A	Dunn et al. [32]	8	ACM	Yes	–	–	–	–	–	–	–	Association suggested by authors, but “no statistical significance owing to the small number of animals”
Lymphoma	Burkitt-like	Aresu et al. [3]	13	TRM	–	–	–	–	–	–	p > 0.05	–	–
	Multicentric	Dobson et al. [28]	–	–	–	–	–	–	–	–	–	–	–
	Multicentric	Kiupel et al. [56]	74	ACM	–	0-9<br>10-12<br>>12	–	–	–	–	All: p = 0.028<br>0-9 vs. 10-12: p = 0.287, HR = 1.670<br>0-9 vs. >12: p = 0.005, HR = 6.380	–	–
	Indolent	Flood-Knapik et al. [39]	73	TRM	–	0, 1, 2, ≥3	0: 41.2 m,<br>1: not reached	2: 21.2 m<br>≥3: not reached	–	p = 0.920	–	–	–
				ACM			0: 17.2 m<br>1: 19.3 m	2: 13.0 m<br>≥3: 33.5 m	–	p = 0.346	–	–	–
	Diffuse small B-cell	Hughes et al. [51]	22 (ACM) or 11 (TRM)	ACM and TRM	–	–	–	–	–	–	–	–	“not correlated”
	Diffuse large B-cell	Sierra Matiz et al. [110]	29	ACM	Yes	<21, ≥21	294 d	129 d	Yes	NS (p > 0.05)	–	–	–
	Any	Valli et al. [125]	183	–	–	≤20, ≥21	–	–	Yes	p = 0.031	–	–	Locally weighted scatter smoothing function with regression line indicated “little evidence” for association with survival
	Small intestinal	Yamazaki et al. [134]	Surgery: 36<br>Chemo: 48	ACM	–	–	–	–	–	–	–	–	“MI expression showed no significant difference in OS between the low (< median) and high (> median) groups (data not shown)”<br><br>Surgery: group 1 (<120d survival) vs.

Tumor type/group	(Sub)type / location	Article	Number of cases with outcome	ACM / TRM	IPD	Cut-off ranges	MST of low MC cases in days (d), weeks (w), or months (m)	MST of high MC cases in days (d) or weeks (w)	Kaplan-Meier curve	Log rank test	Cox proportional hazard regression model, univariable (95% confidence interval)	ROC and AUC	Other statistical tests
													group 2 (≥120 d survival): p = 0.048<br><br>Chemo: group 1 vs. group 2: p = 0.84
Mammary tumors	Malignant	Canadas et al. [16]	85 (all cases)	TRM	–	NHG: ≤8, 9-17, ≥18	–	–	–	NS (p > 0.05)	–	–	–
				TRM	–	Ca-NHG: 0-9, 10-19, ≥20	–	–	–	NS (p > 0.05)	–	–	–
			Unknown (simple, solid, and intraductal papillary carcinomas)	TRM	–	NHG: ≤8, ≥9	–	–	–	p ≤ 0.05	–	–	–
				TRM	–	Ca-NHG: 0-9, ≥10	–	–	–	p ≤ 0.05	–	–	–
	Malignant	Carvalho et al. [17] (2016)	43	TRM	–	I, II, III according to [89]	I: 20.65 m	II: 17.24 m<br>III: 7.88 m	–	p < 0.001	–	–	–
	Malignant	Carvalho et al. [18] (2019)	72	TRM	–	I, II, III according to [43] (Misdorp or Peña?)	–	–	–	p < 0.001	–	–	–
	Malignant	Chen et al. [20]	72	TRM	–	<20, ≥20	–	–	–	–	–	–	1-year survival<br>Low: 87.8%<br>High: 74.2%, p = 0.282<br><br>2-year survival:<br>Low: 78.0%<br>High: 64.5%, p = 0.828
	Malignant	Dutra et al. [33]	17	?	–	Score 1: 0-7<br>Score 2: 8-16<br>Score 3: >17	–	–	Yes, but corrupt	p = 0.032	–	–	Pearson's correlation between MC and survival:<br>Observer 1: r= –0.6047, p = 0.0101;<br>Observer 2:<br>r= –0.5403, p = 0.0251
	Malignant	Liu et al. [64]	70	ACM	–	≤10, >11	–	–	–	p = 0.005	–	–	Multivariate HR = 0.881, 0.166 – 4.676; p = 0.882
	Malignant (invasive carcinoma)	Ressel et al. [95]	35	ACM	–	<9, ≥9	–	–	–	p = 0.01	–	–	TP: 11<br>TN: 14<br>FP: 7<br>FN: 3<br>Sen: 79%<br>Spe: 67%

Tumor type/group	(Sub)type / location	Article	Number of cases with outcome	ACM / TRM	IPD	Cut-off ranges	MST of low MC cases in days (d), weeks (w), or months (m)	MST of high MC cases in days (d) or weeks (w)	Kaplan-Meier curve	Log rank test	Cox proportional hazard regression model, univariable (95% confidence interval)	ROC and AUC	Other statistical tests
	Malignant	Santos et al. [101] (2013)	85	TRM	–	0-9, 10-19, ≥20	0-9: 20.9 m	10-19: 21.2 m<br>≥20: 19.1 m	–	NS (p > 0.05)	–	–	Percentage death or euthanized (2 years):<br>0-9: 25% (9/36)<br>10-19: 16% (4/25)<br>≥20: 37.5% (9/24)
	Carcinoma	Santos et al. [102] (2015)	59	TRM	–	Score 1:<9, Score 2: 9-17,<br>Score 3: >17	–	–	–	Score 1 vs. 2 and 3: NS (p > 0.05)<br>Score 1 and 2 vs. 3: NS (p > 0.05	–	–	–
						Tertiles (values unknown)	–	–	–	NS (p > 0.05	–	–	–
	Any	Dolka et al. [29] (2018)	59	TRM	–	<1, ≥1	–	–	–	–	HR: 7.60 (2.09 – 27.73), p = 0.002	–	TRM-rate:<br>Low: 10.5%<br>High: 47.6%
						<2, ≥2	–	–	–	–	HR: 2.97 (0.96 – 9.19), p = 0.059	–	TRM-rate:<br>Low: 18.8%<br>High: 45.5%
	Any	Dolka et al. [30] (2016)	40	ACM	–	0.0-0.9, 1.0-1.9, ≥2	0.0-0.9: 22 m (not reached according to KM curve)	1.0-1.9: 17 m<br>≥2: 20 m (not reached according to KM curve)	Yes	p = 0.016	–	–	Short survival group (≤18 months:<br>0.0-0.9: n = 6<br>1.0-1.9: n = 6<br>≥2: n = 0<br><br>Long survival group:<br>0.0-0.9: n = 21<br>1.0-1.9: n = 1<br>≥2: n = 6<br><br>Chi-squared test: p = 0.0001
	Any	Mainenti et al. [67]	113	?	–	<10, ≥10 per 1 HPF	22.8 m	13.8 m	Yes	p < 0.05	–	–	–
	Any	Misdorp and Hart [76]	168	?	–	–	–	–	–	–	–	–	p = 0.25
	Neuroendocrine Carcinoma	Nakagaki et al. [82]	8	TRM	Yes	–	–	–	–	–	–	–	–
Mast cell tumor	Cutaneous	Berlato et al. [9] (2013)	95 (all cases	TRM	–	0-5, >5	Not reached	145 d	Yes	p < 0.001	HR = 6.0 (2.9 – 12.2), p < 0.001	–	Scatterplot, Mann-Whitney U test: p < 0.001<br><br>Sen: 55%<br>Spe: 91%<br><br>1-year survival:<br>Low MC: 83%

Tumor type/group	(Sub)type / location	Article	Number of cases with outcome	ACM / TRM	IPD	Cut-off ranges	MST of low MC cases in days (d), weeks (w), or months (m)	MST of high MC cases in days (d) or weeks (w)	Kaplan-Meier curve	Log rank test	Cox proportional hazard regression model, univariable (95% confidence interval)	ROC and AUC	Other statistical tests
													High MC: 26%<br><br>2-year survival:<br>Low MC: 82%<br>High MC: 21%<br><br>3-year survival:<br>Low MC: 82%<br>High MC: 21%
			49 (Patnaik grade II)	TRM	–	0-5, >5	Not reached	145 d	–	p < 0.001	HR = 15.4 (4.2 – 56.9), p < 0.001		Sen: 60%<br>Spe: 95%<br><br>1-year survival:<br>Low MC: 93%<br>High MC: 25%<br><br>2-year survival:<br>Low MC: 90%<br>High MC: 25%<br><br>3-year survival:<br>Low MC: 90%<br>High MC: 25%
	Cutaneous, Patnaik grade II	Berlato et al. [8] (2018)	90	TRM	–	0-5, >5	Not reached	89 d	Yes	p < 0.001	HR = 14.7 (5.5 – 38.9), p < 0.001	Yes<br><br>AUC = 0.78 (0.64-0.91)	Sen: 39%<br>Spe: 99%<br><br>1-year survival:<br>Low MC: 87%<br>High MC: 0%<br><br>2-year survival:<br>Low MC: 87%<br>High MC: 0%<br><br>3-year survival:<br>Low MC: 87%<br>High MC: 0%
	Cutaneous	Elston et al. [36]	57<br>(41 Patnaik II cases)	?	–	0-5, >5	Not reached	8 m	Yes (Patnaik II cases)	p = 0.09	–	–	–
						0, 1-7, >7	0: not reached	1-7: 18 or 15 months?<br>>7: 3 months	Yes (all cases and Patnaik II cases)	p < 0.001<br>p = 0.009	–	–	–
	Cutaneous	Gregorio et al. [45]	43	TRM	–	<5, ≥5	60.9 m (48.1 – 73.6)	7.0 m (1.76 – 12.3)	–	p < 0.001	–	–	TP: 11<br>TN: 22<br>FP: 1

Tumor type/group	(Sub)type / location	Article	Number of cases with outcome	ACM / TRM	IPD	Cut-off ranges	MST of low MC cases in days (d), weeks (w), or months (m)	MST of high MC cases in days (d) or weeks (w)	Kaplan-Meier curve	Log rank test	Cox proportional hazard regression model, univariable (95% confidence interval)	ROC and AUC	Other statistical tests
													FN: 9<br>Sen: 55%<br>Spe: 96%<br><br>1-year survival:<br><5: 71%<br>≥5: 8.3%<br><br>2-year survival:<br><5: 71%<br>≥5: 8.3%
	Cutaneous	Krick et al. [58]	–	–	–	–	–	–	–	–	–	–	–
	Cutaneous	Preziosi et al. [92]	32	TRM	–	Score 0: 0<br>Score 1: 0-2<br>Score 2: >2	–	–	–	p = 0.0057	–	–	–
	Cutaneous	Romansik et al. [98]	99	TRM	–	Group 1: ≤5<br>Group 2: 6-9<br>Group 3: 10-29<br>Group 4: ≥30	Group 1: 70 m	Group 2: 1m<br>Group 3: 3.5m<br>Group 4: <1m	–	p < 0.001<br><br>group 2 vs. 3 vs. 4: NS (p > 0.05)	–	–	Death rate compared to group 1:<br>Group 2: 10x higher<br>Group 3: 7.5x higher<br>Group 4: 14x higher<br>p < 0.001
						Group 1: ≤5<br>Group 2-4: >5	70 m	2 m	Yes	p < 0.001	–	–	–
	Cutaneous	Skor et al. [113]	52	TRM	–	<2, 2-7, >7	–	–	–	p = 0.004	–	–	Multivariable HR: p = 0.313
	Cutaneous	Thamm et al. [120]	74?	?	–	–	–	–	–	p < 0.0001	HR = 1.03 (1.013 – 1.046)	–	–
	Cutaneous	Vascellari et al. [128]	53	?	–	≤5, >5	–	–	Yes	p < 0.001	–	–	Mann-Whitney test: p = 0.001<br><br>Mortality rate:<br>≤5: 8%<br>>5: 80%, p = 0.001<br><br>Sen: 50%<br>Spe: 97.8%
	Cutaneous, multiple (≥3)	O'Connell et al. [85]	57	?	–	<5, ≥5	24.0 m	2.5 m	–	–	HR = 6.61, p = 0.001	–	–
	Cutaneous, Kiupel high grade, stage I	Moore et al. [79]	49	ACM	–	≤5, >5	–	–	–	p = 0.142	–	–	–
						0-7, >7	–	–	–	p = 0.091	–	–	–
						<12, ≥12	1645 d	826 d	–	p = 0.027	HR = 2.66 (1.12 – 6.32)	–	1-year survival<br><12: 85.8%<br>≥12: 61.5%
						<15, ≥15	1645 d	162 d	Yes	p = 0.008	HR = 3.32 (1.37 – 8.04)	–	1-year survival<br><15: 86.9%<br>≥15: 50%

Tumor type/group	(Sub)type / location	Article	Number of cases with outcome	ACM / TRM	IPD	Cut-off ranges	MST of low MC cases in days (d), weeks (w), or months (m)	MST of high MC cases in days (d) or weeks (w)	Kaplan-Meier curve	Log rank test	Cox proportional hazard regression model, univariable (95% confidence interval)	ROC and AUC	Other statistical tests
													2-year survival<br><15: 78.7%<br>≥15: 50%<br><br>3-year survival<br><15: 58.3%<br>≥15: 20%
						<20, ≥20	1645 d	66 d	–	p = 0.001	HR = 5.52 (2.00 – 15.30)	–	1-year survival<br><20: 83.7%<br>≥20: 40%
	Cutaneous and subcutaneous	Horta et al. [50]	149	TRM	–	"cut-point of 2"<br>(≤2, >2)	Not reached	260 d	–	p < 0.0001	–	No<br><br>AUC = 0.816 (0.741 – 0.891)	Sen: 84%<br>Spe: 56%<br><br>Pearson correlation of MC with "outcome" (TRM or presence of metastasis): r = -0.325, p < 0.0001
	Cutaneous and subcutaneous	Knight et al. [57]	139 (subcutaneous and dermal)	TRM	–	≤4, ≥5	–	–	Yes	p < 0.0001	–	–	–
			51 (dermal)	TRM	–	≤4, ≥5	Not reached	119 d	Yes	p < 0.0001	HR = 76.8, p = 0.0002	–	–
			88 (Subcutaneous)	TRM	–	≤4, ≥5	Not reached	205 d	Yes	p < 0.0001	HR = 8.2, p < 0.0001	–	–
	Cutaneous and subcutaneous	Thompson et al. [121]	22	Unknown	–	≤4, >4 resp. ≤7, >7					HR = 1.07 (1.02 – 1.12), p = 0.01	–	TP: 5<br>TN: 16<br>FP: 0<br>FN: 1<br>Sen: 83%<br>Spe: 100%
	Cutaneous and subcutaneous, in Pugs	McNiel et al. [70]	25	TRM	–	–	–	–	–	–	"predicitive of survival" (p < 0.05)	–	–
	Subcutaneous	Bostock et al. [12]	50	?	–	0-4, 5-10, >10	0-4: 40 w<br>5-10: 40 w	>10: 11 w	–	–	–	–	TRM rate:<br>0-4: 23% (8/35)<br>5-10: 25% (1/4)<br>>10: 72% (8/11)<br>p < 0.05<br><br>MC 0-4 vs ≥5<br>TP: 9<br>TN: 27<br>FP: 6<br>FN: 8<br>Sen: 52.9%<br>Spe: 81.8%

Tumor type/group	(Sub)type / location	Article	Number of cases with outcome	ACM / TRM	IPD	Cut-off ranges	MST of low MC cases in days (d), weeks (w), or months (m)	MST of high MC cases in days (d) or weeks (w)	Kaplan-Meier curve	Log rank test	Cox proportional hazard regression model, univariable (95% confidence interval)	ROC and AUC	Other statistical tests
													MC 0-10 vs. >10:<br>TP: 8<br>TN: 30<br>FP: 3<br>FN: 9<br>Sen: 47.1%<br>Spe: 90.1%
	subcutaneous	Gill et al. [40]	43	TRM	–	≤4, >4	–	–	–	–	HR = 3.612 (0.958 – 13.598), p = 0.0578	–	TP: 1<br>TN: 40<br>FP: 0<br>FN: 2<br>Sen: 33%<br>Spe: 100%
	subcutaneous	Newman et al. [83]	53	TRM	–	–	–	–	–	NS (p >0.05)	–	–	–
	subcutaneous	Thompson et al. [122]	306	TRM	–	0, 1-4, >4	–	–	Yes	–	HR = 3.93 (1.18 – 13.05), p = 0.03;<br>HR = 54.68 (17.86 – 167.67), p < 0.01	–	–
						≤4, >4	–	–	Yes	–	HR = 13,92 (5.76 – 33.60), p < 0.01	–	–
	subcutaneous	Thompson et al. [123]	–	–	–	–	–	–	–	–	–	–	–
	skin	Donnelly et al. [31]	–	–	–	–	–	–	–	–	–	–	–
	Pinna	Schwab et al. [107]	28	TRM	–	≤5, >5	Not reached	10	–	p < 0.001	–	–	–
	cutaneous & mucocutaneous, <12 months old	Rigas et al. [96]	–	–	–	–	–	–	–	–	–	–	–
	High-risk	Pecceu et al. [88]	69	ACM	–	≤5, >5	–	–	–	–	HR = 1.84, p < 0.001	–	–
	Grade 3; cutaneous, subcutaneous & mucocutaneous	Hume et al. [52]	20	TRM	–	≤5, >5	305 d	176 d	–	p = 0.02	–	–	–
	intramuscular	Robinson et al. [97]	6	ACM	Yes	–	–	–	–	–	–	–	–
	Oral mucosa	Elliott et al. [35]	21	TRM	–	< or > median	Not reached	150 d	–	–	–	–	Wilcoxon: p = 0.136
						<5, >5	Not reached	120 d	Yes	–	–	–	Wilcoxon: p = 0.017
						<7, >7	Not reached	120 d	–	–	–	–	Wilcoxon: p < 0.017
	Not reported	Van Lelyveld et al. [126]	162	TRM	–	–	–	–	–	–	–	Yes AUC = 0.79	–
						≤5, >5	Not reached	307	Yes	p < 0.001	–	–	1 year survival:<br>≤5: 86.5%<br>>5: 48.3%<br><br>2-year survival<br>≤5: 84.6%<br>>5: 32.2%

Tumor type/group	(Sub)type / location	Article	Number of cases with outcome	ACM / TRM	IPD	Cut-off ranges	MST of low MC cases in days (d), weeks (w), or months (m)	MST of high MC cases in days (d) or weeks (w)	Kaplan-Meier curve	Log rank test	Cox proportional hazard regression model, univariable (95% confidence interval)	ROC and AUC	Other statistical tests
													3-year survival:<br>≤5: 78.9%<br>>5: 16.1%<br><br>Sen: 32.4%<br>Spe: 96%
						<2, ≥2	–	–	Yes	–	–	–	1 year survival:<br><2: 93.4%<br>≥2: 60.5%<br><br>2-year survival<br><2: 93.4%<br>≥2: 50.3%<br><br>3-year survival:<br><2: 89.7%<br>≥2: 35.3%<br><br>Sen: 75.7%<br>Spe: 80%
						<2, 2-7, >7	–	–	Yes	–	–	–	1 year survival:<br><2: 93.3%<br>2-7: 68.5%<br>>7: 29.4%<br><br>2-year survival<br><2: 93.3%<br>2-7: 61.9%<br>>7: 14.7%<br><br>3-year survival:<br><2: 89.3%<br>2-7: 47.1%<br>>7: 0%
				ACM	–	≤5, >5	–	–	–	–	–	–	1 year survival:<br>≤5: 85.1%<br>>5: 38.8%<br><br>2-year survival<br>≤5: 78.8%<br>>5: 25.9%<br><br>3-year survival:<br>≤5: 68.7%<br>>5: 12.9%
						<2, ≥2	–	–	–	–	–	–	1 year survival:<br><2: 91.5%<br>≥2: 57.1%<br><br>2-year survival

Tumor type/group	(Sub)type / location	Article	Number of cases with outcome	ACM / TRM	IPD	Cut-off ranges	MST of low MC cases in days (d), weeks (w), or months (m)	MST of high MC cases in days (d) or weeks (w)	Kaplan-Meier curve	Log rank test	Cox proportional hazard regression model, univariable (95% confidence interval)	ROC and AUC	Other statistical tests
													<2: 86.6%<br>≥2: 45.5%<br><br>3-year survival:<br><2: 78.2%<br>≥2: 30.0%
						<2, 2-7, >7	–	–	–	–	–	–	1 year survival:<br><2: 91.3%<br>2-7: 68.5%<br>>7: 17.9%<br><br>2-year survival<br><2: 86.2%<br>2-7: 59.1%<br>>7: 9.0%<br><br>3-year survival:<br><2: 77.4%<br>2-7: 42.2%<br>>7: 0%
Melanocytic tumors / melanoma	Cutaneous	Laprie et al. [60]	68	TRM	–	<3, =3	–	–	Yes	$p < 0.0001$	–	–	Multivariate HR: $p < 0.0001$
	Cutaneous (exclusive paw pad)	Laver et al. [62]	87	TRM	–	≤20, >20	1792 d	1089 d	Yes	–	Categorial value: HR = 4.1 (1.3 – 12.9), p = 0.014<br>Continuous value: HR = 1.023 (1.006 – 1.041), p = 0.009	–	–
	Cutaneous	Silvestri et al. [111]	77	?	–	–	–	–	–	–	HR = 1.02 (1.00 – 1.03), p = 0.043	–	–
	Cutaneous and oral	Martinez et al. [68]	41	TRM	Yes	–	–	–	–	–	–	–	Odds ratio: 1.37, p = 0.004
	Cutaneous and oral	Millanta et al. [75]	38	ACM	–	–	–	–	–	–	–	–	Mean MC:<br>Alive: 2.6<br>Dead: 13.4<br>Bonferroni test: p = 0.001
	Cutaneous and oral	Porcellato et al. [90]	75	TRM	–	<20.5, >20.5	–	–	–	–	HR = 1.011 (1.003 – 1.020), p = 0.005	AUC = 0.781 (0.673 – 0.889)	Sen: 81%<br>Spe: 80%
	Skin, lip, oral	Bostock [11]	Skin: 85	TRM	–	≤2, ≥3	104 w	30 w	–	–	–	–	2-year death rate:<br>≤2: 6/59 (10%)<br>≥3: 19/26 (73%)<br><br>TP: 19<br>TN: 53<br>FP: 7<br>FN: 6

Tumor type/group	(Sub)type / location	Article	Number of cases with outcome	ACM / TRM	IPD	Cut-off ranges	MST of low MC cases in days (d), weeks (w), or months (m)	MST of high MC cases in days (d) or weeks (w)	Kaplan-Meier curve	Log rank test	Cox proportional hazard regression model, univariable (95% confidence interval)	ROC and AUC	Other statistical tests
													Sen: 76%<br>Spe: 88%
			Oral: 31	TRM	–	–	–	–	–	–	–	–	"no correlation"
	Skin, lip, nail bed	Schultheiss [105]	Lip: 32	ACM	–	≤2, >2	–	–	–	–	–	–	TP: 11<br>TN: 6<br>FP: 11<br>FN: 4<br>Sen: 73%<br>Spe: 35%<br><br>"did not correlate with clinical outcome"
			Skin: 24	ACM	–	≤2, >2	–	–	–	–	–	–	"Low and high MC group were equally split between either outcome"
			Nail bed: 14	ACM	–	≤2, >2	–	–	–	–	–	–	"did not predict survival time"
	Intraocular	Giuliano et al. [42]	36	?	–	–	–	–	–	NS	–	–	"no statistically significant difference related to survival time"
	Ocular (intraocular and scleral)	Wilcock and Peiffer [130]	–	–	–	–	–	–	–	–	–	–	–
	Oral	Bergin et al. [7]	76	TRM	–	<4, ≥4	–	–	Yes	Breslow test: p < 0.001	HR = 1.016 (1.008 – 1.025), p < 0.001	Yes, AUC = 0.859 (0.76 – 0.95), p < 0.001	Sen: 90%<br>Spe: 84%
	Oral	Baja et al. [5]	64	–	–	<4, ≥4	–	–	–	–	HR = 1.731 (0.761 – 4.648), p = 0.2251	–	–
	Oral	Camerino et al. [15]	68	–	–	–	–	–	–	–	–	–	"only correlated (r = -0.39, Spearman) with cases with bone invasion"
	Oral	Hahn et al. [47]	32	TRM	–	≤3, >3 per 1 HPF!	718/823 d	193/271 d	–	p = 0.04	–	–	–
	Oral	Prouteau et al. [93]	73	TRM	–	≤6, >6	–	–	Yes	p = 0.0011	HR = 2.49 (1.49 – 4.14)	–	Sen: 80%<br>Spe: 37%
						≤4, >4	–	–	–	–	–	–	Sen: 83%<br>Spe: 28%
	Oral	Ramos-Vara et al. [94]	40	TRM ?	–	–	–	–	–	–	–	–	Mann-Whitney rank sum test: ns (p > 0.05)
	Oral	Vargas et al. [127]	27	TRM	–	–	–	–	–	p = 0.1604	–	–	–

Tumor type/group	(Sub)type / location	Article	Number of cases with outcome	ACM / TRM	IPD	Cut-off ranges	MST of low MC cases in days (d), weeks (w), or months (m)	MST of high MC cases in days (d) or weeks (w)	Kaplan-Meier curve	Log rank test	Cox proportional hazard regression model, univariable (95% confidence interval)	ROC and AUC	Other statistical tests
	Oral	Wei et al. [129]	66	TRM	–	<10, ≥10	–	–	Yes	p = 0.0257 (glass slide), p = 0.0073 (WSI 20x)	–	–	–
	Oral, osteo-cartilaginous differentiation	Sanchez et al. [100]	–	–	–	–	–	–	–	–	–	–	–
	Non-ocular (cutaneous and oral)	Spangler and Kass [116]	Oral: 73	–	–	<14, ≥14	–	–	–	–	HR = 1.019 (1.010 – 1.028), p < 0.001	–	Sen: 77%<br>Spe: 75%
			Feet or lip: 73	–	–	<5, ≥5 or <16, ≥16	–	–	–	–	HR = 1.088 (1.038 – 1.141), p < 0.001	–	<5, ≥5:<br>Sen: 90%<br>Spe: 78%<br><br><16, ≥16:<br>Sen: 75%<br>Spe: 83%
			Cutaneous: 227	–	–	–	–	–	–	–	HR = 1.159 (1.086 – 1.37), p < 0.001	–	–
Oesophageal sarcoma	Spirocera-induced	Pazzi et al. [87]	17	?	–	Low: 1-4<br>Moderate: 5-10<br>High: >10	–	–	–	p = 0.282	–	–	–
Osteo-sarcoma	Appendicular, small-breed dogs	Amsellem et al. [2]	Unknown	–	–	–	–	–	–	–	p > 0.05	–	–
	Appendicular	Guim et al. [46]	22	–	–	–	–	–	–	p = 0.24	–	–	–
	Appendicular	Moore et al. [77]	155	?	–	5-25, 26-50, 51-75, >75	–	–	–	–	p > 0.05	–	–
	Appendicular	Saam et al. [99]	35	TRM	–<br>–	<5,<br>5-20<br>>20	415 d	193 d<br>136 d	–	p = 0.004	–	–	–
						<5, ≥5	415 d	177 d	–	p = 0.03	HR = 0.4 (0.1 – 0.9)	–	–
	Appendicular	Schott et al. [104]	85; three evaluators (A-C)	ACM, per 3 HPF	–	–	–	–	–	–	A: HR = 1.02 (0.99 – 1.05), p = 0.211	–	–
					–	–	–	–	–	–	B: HR = 1.03 (0.99 – 1.06), p = 0.135	–	–
					–	–	–	–	–	–	C: HR = 1.00 (0.96 – 1.03), p = 0.99	–	–
				ACM, per 10 HPF	–	–	–	–	–	–	A: HR = 1.00 (0.99 – 1.01), p = 0.434	–	–
					–	–	–	–	–	–	B: HR = 1.01 (0.99 – 1.02), p = 0.335	–	–
					–	–	–	–	–	–	C: HR = 0.99 (0.98 – 1.01) p = 0.893	–	–
					–	–	–	–	–	–	A: HR = 1.02 (0.99 – 1.05), p = 0.120	–	–

Tumor type/group	(Sub)type / location	Article	Number of cases with outcome	ACM / TRM	IPD	Cut-off ranges	MST of low MC cases in days (d), weeks (w), or months (m)	MST of high MC cases in days (d) or weeks (w)	Kaplan-Meier curve	Log rank test	Cox proportional hazard regression model, univariable (95% confidence interval)	ROC and AUC	Other statistical tests
				TRM, per 3 HPF	–	–	–	–	–	–	B: HR = 1.04 (1.00 – 1.08), p = 0.030	–	–
					–	–	–	–	–	–	C: HR = 1.00 (0.96 – 1.04), p = 0.903	–	–
				TRM, per 10 HPF	–	–	–	–	–	–	A: HR = 1.00 (0.99 – 1.01), p = 0.303	–	–
					–	–	–	–	–	–	B: HR = 1.01 (0.99 – 1.02), p = 0.298	–	–
					–	–	–	–	–	–	C: HR = 0.99 (0.98 – 1.01), p = 0.924	–	–
	Mandibular	Coyle et al. [25]	43	ACM	–	≤20, >20	637	267	–	p = 0.418	–	–	–
						≤40, >40	637	256	–	p = 0.057	–	–	–
	Surface	Cook et al. [24]	11	ACM	–	<4, ≥4	555	516	–	p = 0.98	–	–	–
	Any	Kirpensteijn et al. [55]	–	–	–	–	–	–	–	–	–	–	–
	Any	Loukopoulos et al. [65]	–	–	–	–	–	–	–	–	–	–	–
Pheochromocytoma	N/A	Zini et al. [135]	24	STD	–	>3	–	–	–	–	–	–	"Not significantly associated with survival to discharge from hospitalization"
Pulmonary neoplasia	Carcinoma	McNiel et al. [69]	67	ACM	–	Scores:<br>1: 1-10<br>2: 11-20<br>3: 21-30<br>4: ≥31	–	–	–	–	Score 1 vs. 2: HR = 3.45 (1.51-7.88)<br>Score 1 vs. 3: HR = 2.32 (0.82-6.57)<br>Score 1 vs. 4: HR = 4.24 (1.29-13.95)	–	"significantly associated"
	Any primary	Able et al. [1]	–	–	–	–	–	–	–	–	–	–	–
	Any primary	McPhetridge [71]	284	?	–	≤10, >10-≤20, >20-≤30, >30	477d<br>388d	244d<br>89d	–	–	p < 0.001	–	–
Renal cell carcinoma	N/A	Carvalho et al. [19]	51	ACM	–	<10, 10-30, >30	545d<br>532d	120d	Yes	p = 0.003	–	–	Survival 1 year:<br>MC <10: 50%<br>MC = 10-30: 58%<br>MC >10: 12.5%<br><br>Survival s year:<br>MC <10: 14%<br>MC = 10-30: 21%<br>MC >10: 0%
	N/A	Edmondson et al. [34]	70	ACM	–	<10, 10-30, >30	1184d<br>452d	187 d	Yes	p < 0.0001	HR = 2.65 (1.06 – 6.63), p = 0.0371<br>HR = 12.24 (5.15 – 29.1), p < 0.0001	–	–
				TRM	–						HR = 3.94 (1.42 – 10.9), p = 0.0083<br>HR = 16.46 (6.3 – 43.1), p <0.001		
Salivary gland neoplasia	N/A	Hammer et al. [48]	24	?	–	–	–	–	–	NS (P > 0.05)	–	–	–

Tumor type/group	(Sub)type / location	Article	Number of cases with outcome	ACM / TRM	IPD	Cut-off ranges	MST of low MC cases in days (d), weeks (w), or months (m)	MST of high MC cases in days (d) or weeks (w)	Kaplan-Meier curve	Log rank test	Cox proportional hazard regression model, univariable (95% confidence interval)	ROC and AUC	Other statistical tests
Sarcomas, non-osteogenic	Skin	Bray et al. [14]	350	TRM	–	0-9, 10-19, >19	–	–	Yes	–	–	–	Chi-squared test: $x^2 = 11.8$, p = 0.003<br><br>Multivariate HR = 2.0 (1.0 – 1.1), p = 0.04
	Skin	Ettinger et al. [38]	54	TRM	–	<10, ≥10	–	–	–	–	HR = 4.1 (1.32 – 12.82), p = 0.015	–	p = 0.004
	Skin	Heller et al. [49]	–	–	–	–	–	–	–	–	–	–	–
	Skin	Kuntz et al. [59]	75	TRM	–	<10, 10-19, >19 resp. <20, ≥20	<10: 1,444 d<br>10-19: 532 d	>19: 236 d	Yes	–	"significant"	–	–
	Skin (extraoral)	Simon et al. [112]	27	TRM	–	0-9, 10-19, ≥20	0-9: 1138 d	10-19: 146 and 150 d<br>≥20: 76, 193 and 250 d		p = 0.001	–	–	Multivariate HR: "significant"
						0-9, >9	1138 d	150 d	Yes	p = 0.0001	–	–	–
	(Sub)cutaneous, with recurrence	Chiti et al. [22]	23	TRM	Yes	–	–	–	–	–	–	–	–
	Subcutaneous	McSporran [72]	–	–	–	–	–	–	–	–	–	–	–
	Skin	Bostock and Dye [13]	160	?	–	0-8, ≥9	118 w	49 w	–	–	–	–	p < 0.01
	High grade (MC ≥9)	Crownshaw et al. [26]	41	?	–	–	–	–	–	–	HR = 1.042 (0.992 – 1.094), p = 0.102	–	–
	High grade, any location	Selting et al. [108]	39	TRM	–	<20, ≥20	376 d	309	–	p = 0.935	–	–	–
						<20, 20-49, ≥50	376 d	503 d<br>229 d	–	p = 0.107	–	–	–
	Abdominal visceral	Linden et al. [63]	31	TRM	–	<9, ≥9	Not reached	269 d	Yes	p = 0.037	–	–	continuous MC values: p = 0.95
	Gastrointestinal sacomas	Del Alcazar [27]	42	ACM	–	≤9, >9	1266 d	293 d	–	p = 0.05	–	–	OR: 0.19 (0.038-0.93), p = 0.04
	Gastrointestinal stromal tumors	Berger et al. [6]	–	–	–	–	–	–	–	–	–	–	–
	Gastrointestinal stromal tumors	Gillespie et al. [41]	16	ACM	Yes	–	–	–	–	–	–	–	–
	Hemangiopericytoma, skin	Graves et al. [44]	–	–	–	–	–	–	–	–	–	–	–
	Myxosarcoma, cutaneous	Iwaki et al. [53]	29	ACM	–	<10, ≥10	<10: 1393 d	>10: 433 d	Yes	p = 0.109	–	–	–
	Peripheral nerve sheath tumor, skin	Boos et al. [10]	–	–	–	–	–	–	–	–	–	–	–
	Perivascular wall tumor, cutaneous	Chiti et al. [21]	102	TRM	–	–	–	–	–	–	HR = 1.055 (1.018 – 1.093), p = 0.003	–	–
	Perivascular wall tumor, cutaneous	Stefanello et al. [118]	25	TRM	–	–	–	–	–	–	–	–	"correlated"

Tumor type/group	(Sub)type / location	Article	Number of cases with outcome	ACM / TRM	IPD	Cut-off ranges	MST of low MC cases in days (d), weeks (w), or months (m)	MST of high MC cases in days (d) or weeks (w)	Kaplan-Meier curve	Log rank test	Cox proportional hazard regression model, univariable (95% confidence interval)	ROC and AUC	Other statistical tests
	Smooth muscle tumors, any location	Avallone et al. [4]	–	–	–	–	–	–	–	–	–	–	–
	Intestinal sarcomas	Maas et al. [66]	61	ACM	–	–	–	–	–	–	–	–	“no significant importance”
Splenic tumors (exclusive hemangiosarcoma)	Stromal sarcoma	Moore et al. [78]	8	ACM	–	–	–	–	–	–	–	–	“not significant”
	“Fibrohistiocytic nodules”	Spangler and Kass [117]	93	?	–	–	–	–	–	–	–	–	p < 0.062
	Mesenchymal (non-angiomatous, non-lymphomatous)	Spangler et al. [115]	68	?	–	<9, ≥9	“longest”	1-2 m	Yes	p < 0.001	–	–	–
	Stromal sarcoma (non-angiomatous, non-myogenic)	Wittenberns et al. [132]	24	ACM	–	<9, ≥9	67 d	439 d	Yes	p = 0.01	HR = 4.12 (1.4-12.0)	–	–
Squamous cell carcinoma	Nasal planum	Lascelles et al. [61]	–	–	–	–	–	–	–	–	–	–	–
	Dermal	Willcox et al. [131]	93	ACM	–	–	–	–	–	–	HR = 0.98 (0.96 - 0.99), p = 0.024	–	–
Synovial sarcoma	N/A	Vail et al. [124]	34	TRM	–	<25, ≥25	48 (Not reached)	9 months	Yes	p = 0.003	–	–	–
Thymic tumors	Epithelial	Yale et al. [133]	30	ACM	–	–	–	–	–	–	p > 0.10 (univariate)	–	–

ACM, all-cause mortality; TRM, tumor-related mortality; IPD, individual patient data; MST, median survival time; N/A, not available; - , not available; NS, not significant (p > 0.05) if actual p-value is not provided in the manuscript; HR, hazard ratio; ROC, receiver-operating-characteristics curve; AUC, area under the ROC curve; TP, true positives (MC above cut-off and presence of outcome event); TN, true negatives (MC below cut-off and lack of outcome event); FP, false positives (MC above cut-off and lack of outcome event); FN, false negatives (MC below cut-off and presence of outcome event); Sen, sensitivity; Spe, specificity; STD, survival to discharge from hospitalization; MI, mitotic index; NHG, Nottingham Histologic Grade; Ca-NHG, canine Nottingham Histologic Grade; OR, odds ratio; OS, overall survival

Disease Progression

Supplemental Table S6. Summary of the prognostic value of the mitotic count (MC) with regard to progression/disease-free interval (PFI).

Tumor type/group	(Sub)type / location	Article	Number of cases with outcome	Outcome	IPD	Cut-off ranges	Median interval of low MC value in cases (d) or months (m)	Median interval of high MC cases in days (d) or months (m)	Kaplan-Meier curve	Log rank test	Cox proportional hazard regression model, univariable (95% confidence interval)	ROC and AUC	Other statistical tests
Apocrine gland anal sac adenocarcinoma	N/A	Morello et al. [81]	24	PFI	–	<16.5, ≥16.5	283 d	273 d	–	p = 0.81	–	–	–
	N/A	Pradel et al. [91]	24	PFI	–	<8, ≥8	760 d	419 d	–	p = 0.048	–	–	–
	N/A	Schlag et al. [103]	–	–	–	–	–	–	–	–	–	–	–
	Early stage	Skorupski et al. [114]	–	–	–	–	–	–	–	–	–	–	–
	With lymph node metastasis	Tanis et al. [119]	48	PFI	–	<8, ≥8	188 d	111 d	–	p = 0.83	–	–	–
Glial tumors	N/A	Merickel et al. [74]	–	–	–	–	–	–	–	–	–	–	–
Hemangio-sarcoma	Any other than skin	Ogilvie et al. [86]	46	PFI	–	Score 0: ≤10 Score 1: 11-20 Score 2: 21-20 Score 3: >30	–	–	–	p = 0.044	–	–	–
	Nonvisceral: skin, eye, bone, muscle	Schultheiss [106]	–	–	–	–	–	–	–	–	–	–	–
	Cutaneous	Nobrega et al. [84]	–	–	–	–	–	–	–	–	–	–	–
	Splenic	Kim et al. [54]	–	–	–	–	–	–	–	–	–	–	–
	Splenic	Moore et al. [80]	–	–	–	–	–	–	–	–	–	–	–
	Subcutaneous & intramuscular	Shiu et al. [109]	71	PFI	–	–	–	–	–	–	HR = 1.13; p = 0.060	–	–
	Falciform fat	Mendez et al. [73]	4	PFI	Yes	–	–	–	–	–	–	–	–
Insulinoma	N/A	Cleland et al. [23]	–	–	–	–	–	–	–	–	–	–	–
	N/A	Dunn et al. [32]	–	–	–	–	–	–	–	–	–	–	–
Lymphoma	Burkitt-like	Aresu et al. [3]	13	PFI	–	–	–	–	–	–	p > 0.05	–	–
	Multicentric	Dobson et al. [28]	–	–	–	–	–	–	–	–	–	–	–
	Multicentric	Kiupel et al. [56]	71	PFI	–	–	–	–	–	–	All: p = 0.137<br>0-9 vs. 10-12: HR = 1.597, p = 0.390<br>0-9 vs. >12: HR = 17.36, p = 0.020	–	–
	Indolent	Flood-Knapik et al. [39]	–	–	–	–	–	–	–	–	–	–	–

Tumor type/group	(Sub)type / location	Article	Number of cases with outcome	Outcome	IPD	Cut-off ranges	Median interval of low MC value in cases (d) or months (m)	Median interval of high MC cases in days (d) or months (m)	Kaplan-Meier curve	Log rank test	Cox proportional hazard regression model, univariable (95% confidence interval)	ROC and AUC	Other statistical tests
	Diffuse small B-cell	Hughes et al. [51]	–	–	–	–	–	–	–	–	–	–	–
	Diffuse large B-cell	Sierra Matiz et al. [110]	–	–	–	–	–	–	–	–	–	–	–
	Any	Valli et al. [125]	–	–	–	–	–	–	–	–	–	–	–
	Small intestinal	Yamazaki et al. [134]	–	–	–	–	–	–	–	–	–	–	–
Mammary tumors	Malignant	Canadas et al. [16]	–	–	–	–	–	–	–	–	–	–	–
	Malignant	Carvalho et al. [17] (2016)	–	–	–	–	–	–	–	–	–	–	–
	Malignant	Carvalho et al. [18] (2019)	–	–	–	–	–	–	–	–	–	–	–
	Malignant	Chen et al. [20]	–	–	–	–	–	–	–	–	–	–	–
	Malignant	Dutra et al. [33]	–	–	–	–	–	–	–	–	–	–	–
	Malignant	Liu et al. [64]	–	–	–	–	–	–	–	–	–	–	–
	Malignant	Ressel et al. [95]	–	–	–	–	–	–	–	–	–	–	–
	Malignant	Santos et al. [101]	85	PFI	–	0-9, 10-19, ≥20	0-9: 18.6 m	10-19: 18.9 m<br>≥20: 16.7 m	–	NS (p > 0.05)	–	–	Percentage recurrence or distant metastasis (2 years):<br>0-9: 27.8% (10/36)<br>10-19: 24% (6/25)<br>≥20: 41.7% (10/24)
	Carcinoma	Santos et al. [102]	59	PFI	–	Score 1:<9, Score 2: 9-17, Score 3: >17	–	–	–	Score 1 vs. 2 and 3: NS (p > 0.05)<br>Score 1 and 2 vs. 3: NS (p > 0.05	–	–	–
						Tertiles (values unknown)	–	–	–	NS (p > 0.05	–	–	–
	Any	Dolka et al. [29] (2018)	–	–	–	–	–	–	–	–	–	–	–
	Any	Dolka et al. [30] (2016)	–	–	–	–	–	–	–	–	–	–	–
	Any	Mainenti et al. [67]	–	–	–	–	–	–	–	–	–	–	–
	Any	Misdorp and Hart [76]	–	–	–	–	–	–	–	–	–	–	–
	Neuroendocrine Carcinoma	Nakagaki et al. [82]	–	–	–	–	–	–	–	–	–	–	–
Mast cell tumor	Cutaneous	Berlato et al. [9] (2013)	–	–	–	–	–	–	–	–	–	–	–
	Cutaneous	Berlato et al. [8] (2018)	–	–	–	–	–	–	–	–	–	–	–
	Cutaneous	Elston et al. [36]	–	–	–	–	–	–	–	–	–	–	–
	Cutaneous	Gregorio et al. [45]	–	–	–	–	–	–	–	–	–	–	–

Tumor type/group	(Sub)type / location	Article	Number of cases with outcome	Outcome	IPD	Cut-off ranges	Median interval of low MC value in cases (d) or months (m)	Median interval of high MC cases in days (d) or months (m)	Kaplan-Meier curve	Log rank test	Cox proportional hazard regression model, univariable (95% confidence interval)	ROC and AUC	Other statistical tests
	Cutaneous	Krick et al. [58]	–	–	–	–	–	–	–	–	–	–	–
	Cutaneous	Preziosi et al. [92]	32	PFI	–	Score 0: 0<br>Score 1: 0-2<br>Score 2: >2	–	–	–	NS (p >0.05)	–	–	–
	Cutaneous	Romansik et al. [98]	–	–	–	–	–	–	–	–	–	–	–
	Cutaneous	Skor et al. [113]	52	PFI	–	<2, 2-7, >7	–	–	–	p = 0.005	–	–	–
	Cutaneous	Thamm et al. [120]	74?	PFI	–	–	–	–	–	p < 0.0001	HR = 1.034 (1.019 – 1.049)	–	–
	Cutaneous	Vascellari et al. [128]	–	–	–	–	–	–	–	–	–	–	–
	Cutaneous, multiple (≥3)	O’Connell et al. [85]	57	PFI	–	<5, ≥5	7.0 m	1.0 m	–	–	HR = 2.52, p = 0.071	–	–
	Cutaneous, Kiupel high grade, stage I	Moore et al. [79]	–	–	–	–	–	–	–	–	–	–	–
	Cutaneous and subcutaneous	Horta et al. [50]	–	–	–	–	–	–	–	–	–	–	–
	Cutaneous and subcutaneous	Knight et al. [57]	88 (subcutaneous)	PFI	–	–	–	–	Yes	p < 0.0001	HR = 6.2, p = 0.0001	–	–
			51 (dermal)	PFI	–	–	–	–	Yes	p = 0.0002	HR = 11.1, p = 0.004	–	–
	Cutaneous and subcutaneous	Thompson et al. [121]	22	PFI	–	–	–	–	–	–	HR = 1.05 (1.01 – 1.09), p = 0.01	–	–
	Cutaneous and subcutaneous, in Pugs	McNiel et al. [70]	–	–	–	–	–	–	–	–	–	–	–
	Subcutaneous	Bostock et al. [12]	–	–	–	–	–	–	–	–	–	–	–
	Subcutaneous	Gill et al. [40]	43	PFI	–	–	–	–	–	–	HR = 1.852 (1.061 – 3.235), p = 0.0302	–	–
				PFI	–	–	–	–	–	–	HR = 3.573 (0.988 – 12.924), p = 0.0522	–	–
	Subcutaneous	Newman et al. [83]	–	–	–	–	–	–	–	–	–	–	–
	Subcutaneous	Thompson et al. [122]	306	PFI	–	0, 1-4, >4	–	–	–	–	HR = 1.89 (1.06 – 3.41), p = 0.03<br>HR = 12.42 (6.53 – 23.62), p < 0.01	–	–
						≤4, >4	–	–	–	–	HR = 6.55 (3.42 – 12.50), p < 0.01	–	–
	Subcutaneous	Thompson et al. [123]	–	–	–	–	–	–	–	–	–	–	–
	Skin	Donnelly et al. [31]	–	–	–	–	–	–	–	–	–	–	–
	Pinna	Schwab et al. [107]	–	–	–	–	–	–	–	–	–	–	–
	Cutaneous & mucocutaneous, <12 months old	Rigas et al. [96]	16	PFI	Yes	–	–	–	–	–	–	–	–

Tumor type/group	(Sub)type / location	Article	Number of cases with outcome	Outcome	IPD	Cut-off ranges	Median interval of low MC value in cases (d) or months (m)	Median interval of high MC cases in days (d) or months (m)	Kaplan-Meier curve	Log rank test	Cox proportional hazard regression model, univariable (95% confidence interval)	ROC and AUC	Other statistical tests
	High-risk	Pecceu et al. [88]	69	PFI	–	≤5, >5	–	–	–	–	HR = 1.80, p < 0.001	–	–
	Grade 3; cutaneous, subcutaneous & mucocutaneous	Hume et al. [52]	20	PFI	–	≤5, >5	198 d	37 d	–	p = 0.03	–	–	–
	intramuscular	Robinson et al. [97]	6	PFI	Yes	–	–	–	–	–	–	–	–
	Oral mucosa	Elliott et al. [35]	21	PFI	–	< or > median	347 d	120 d	–	p = 0.015	–	–	–
						<5, >5	210 d	90 d	Yes	p = 0.001	–	–	–
						<7, >7	210 d	90 d	–	p = 0.001	–	–	–
	Not reported	Van Lelyveld et al. [126]	–	–	–	–	–	–	–	–	–	–	–
Melanocytic tumors / melanoma	Cutaneous (exclusive paw pad)	Laprie et al. [60]	–	–	–	–	–	–	–	–	–	–	–
	Cutaneous	Laver et al. [62]	87	PFI	–	≤17, >17	1374 d	679 d	Yes	–	Categorial values: HR = 3.2 (1.2 – 8.6), p = 0.017 Continuous values: HR = 1.026 (1.007 – 1.046), p = 0.008	–	–
	Cutaneous	Silvestri et al. [111]	77	PFI	–	–	–	–	–	–	NS (p > 0.05)	–	–
	Cutaneous and oral	Martinez et al. [68]	–	–	–	–	–	–	–	–	–	–	–
	Cutaneous and oral	Millanta et al. [75]	–	–	–	–	–	–	–	–	–	–	–
	Cutaneous and oral	Porcellato et al. [90]	–	–	–	–	–	–	–	–	–	–	–
	Skin, lip, oral	Bostock [11]	–	–	–	–	–	–	–	–	–	–	–
	Skin, lip, nail bed	Schultheiss [105]	–	–	–	–	–	–	–	–	–	–	–
	Intraocular	Giuliano et al. [42]	–	–	–	–	–	–	–	–	–	–	–
	Ocular (intraocular and scleral)	Wilcock and Peiffer [130]	–	–	–	–	–	–	–	–	–	–	–
	Oral	Bergin et al. [7]	–	–	–	–	–	–	–	–	–	–	–
	Oral	Baja et al. [5]	64	PFI	–	<4, ≥4	–	–	–	–	HR = 1.885 (0.790 – 5.571), p = 0.1939	–	–
	Oral	Camerino et al. [15]	–	–	–	–	–	–	–	–	–	–	–
	Oral	Hahn et al. [47]	32	PFI	–	≤3, >3	653/786	79/93	–	p < 0.01	–	–	–
	Oral	Prouteau et al. [93]	–	–	–	–	–	–	–	–	–	–	–
	Oral	Ramos-Vara et al. [94]	–	–	–	–	–	–	–	–	–	–	–
	Oral	Vargas et al. [127]	–	–	–	–	–	–	–	–	–	–	–
	Oral	Wei et al. [129]	–	–	–	–	–	–	–	–	–	–	–
	Oral, osteo-cartilaginous differentiation	Sanchez et al. [100]	–	–	–	–	–	–	–	–	–	–	–

Tumor type/group	(Sub)type / location	Article	Number of cases with outcome	Outcome	IPD	Cut-off ranges	Median interval of low MC value in cases (d) or months (m)	Median interval of high MC cases in days (d) or months (m)	Kaplan-Meier curve	Log rank test	Cox proportional hazard regression model, univariable (95% confidence interval)	ROC and AUC	Other statistical tests
	Non-ocular (cutaneous and oral)	Spangler and Kass [116]	–	–	–	–	–	–	–	–	–	–	–
Esophageal sarcoma	Spirocera-induced	Pazzi et al. [87]	–	–	–	–	–	–	–	–	–	–	–
Osteo-sarcoma	Appendicular, small-breed dogs	Amsellem et al. [2]	Unknown	PFI	–	–	–	–	–	–	p > 0.05	–	–
	Appendicular	Guim et al. [46]	–	–	–	–	–	–	–	–	–	–	–
	Appendicular	Moore et al. [77]	–	–	–	–	–	–	–	–	–	–	–
	Appendicular	Saam et al. [99]	–	–	–	–	–	–	–	–	–	–	–
	Appendicular	Schott et al. [104]	–	–	–	–	–	–	–	–	–	–	–
	Mandibular	Coyle et al. [25]	–	–	–	–	–	–	–	–	–	–	–
	Surface	Cook et al. [24]	11	PFI	–	<4, ≥4	497 d	350 d	–	p = 0.29	–	–	–
	Any	Kirpensteijn et al. [55]	99	PFI	–	–	–	–	–	–	–	–	Multivariable HR = 1.06 (1.02 – 1.10), p = 0.002
	Any	Loukopoulos et al. [65]	–	–	–	–	–	–	–	–	–	–	–
Pheochromocytoma	N/A	Zini et al. [135]	–	–	–	–	–	–	–	–	–	–	–
Pulmonary neoplasia	Carcinoma	McNiel et al. [69]	67	PFI	–	Scores:<br>1: 1-10<br>2: 11-20<br>3: 21-30<br>4: ≥31	–	–	–	–	Score 1 vs. 2: HR = 2.64 (1.26-5.53)<br>Score 1 vs. 3: HR = 1.40 (0.52-3.73)<br>Score 1 vs. 4: HR = 2.73 (0.88-8.48)	–	"significantly associated"
	Any primary	Able et al. [1]	54	PFI	–	–	–	–	–	–	HR: 1.03 (1.009-1.060), p = 0.008	–	–
	Any primary	McPhetridge [71]	–	–	–	–	–	–	–	–	–	–	–
Renal cell carcinoma	N/A	Carvalho et al. [19]	–	–	–	–	–	–	–	–	–	–	–
	N/A	Edmondson et al. [34]	–	–	–	–	–	–	–	–	–	–	–
Salivary gland neoplasia	N/A	Hammer et al. [48]	–	–	–	–	–	–	–	–	–	–	–
Sarcomas, non-osteogenic	Skin	Bray et al. [14]	–	–	–	–	–	–	–	–	–	–	–
	Skin	Ettinger et al. [38]	–	–	–	–	–	–	–	–	–	–	–
	Skin	Heller et al. [49]	–	–	–	–	–	–	–	–	–	–	–
	Skin	Kuntz et al. [59]	–	–	–	–	–	–	–	–	–	–	–
	Skin (extraoral)	Simon et al. [112]	–	–	–	–	–	–	–	–	–	–	–
	Cutaneous	Chiti et al. [22]	–	–	–	–	–	–	–	–	–	–	–
	Subcutaneous	McSporran [72]	–	–	–	–	–	–	–	–	–	–	–
	Skin	Bostock and Dye [13]	–	–	–	–	–	–	–	–	–	–	–
	High grade (MC ≥9)	Crownshaw et al. [26]	41	PFI	–	–	–	–	–	–	HR = 1.115 (1.009 – 1.233), p = 0.033	–	–
	High grade, any location	Selting et al. [108]	39	PFI	–	<20, ≥20	216 d	239 d	–	p = 0.717	–	–	–

Tumor type/group	(Sub)type / location	Article	Number of cases with outcome	Outcome	IPD	Cut-off ranges	Median interval of low MC value in cases (d) or months (m)	Median interval of high MC cases in days (d) or months (m)	Kaplan-Meier curve	Log rank test	Cox proportional hazard regression model, univariable (95% confidence interval)	ROC and AUC	Other statistical tests
						<20, 20-49, ≥50	216 d	328 d<br>126 d	–	p = 0.093	–	–	–
	Abdominal visceral	Linden et al. [63]	22	PFI	–	<5, 5-10, >10	–	–	–	p < 0.001	–	–	–
	Gastrointestinal sarcomas	Del Alcazar [27]	–	–	–	–	–	–	–	–	–	–	–
	Gastrointestinal stromal tumors	Berger et al. [6]	–	–	–	–	–	–	–	–	–	–	–
	Gastrointestinal stromal tumors	Gillespie et al. [41]	–	–	–	–	–	–	–	–	–	–	–
	Hemangiopericytoma, skin	Graves et al. [44]	–	–	–	–	–	–	–	–	–	–	–
	Myxosarcoma, cutaneous	Iwaki et al. [53]	–	–	–	–	–	–	–	–	–	–	–
	Peripheral nerve sheath tumor, skin	Boos et al. [10]	–	–	–	–	–	–	–	–	–	–	–
	Perivascular wall tumor, cutaneous	Chiti et al. [21]	–	–	–	–	–	–	–	–	–	–	–
	Perivascular wall tumor, cutaneous	Stefanello et al. [118]	–	–	–	–	–	–	–	–	–	–	–
	Smooth muscle tumors, any location	Avallone et al. [4]	–	–	–	–	–	–	–	–	–	–	–
	Intestinal sarcomas	Maas et al. [66]	61	PFI	–	–	–	–	–	–	–	–	No significant importance
Splenic tumors (exclusive hemangiosarcoma)	Stromal sarcoma	Moore et al. [78]	–	–	–	–	–	–	–	–	–	–	–
	"Fibrohistiocytic nodules"	Spangler and Kass [117]	–	–	–	–	–	–	–	–	–	–	–
	Mesenchymal (non-angiomatous, non-lymphomatous)	Spangler et al. [115]	–	–	–	–	–	–	–	–	–	–	–
	Stromal sarcoma (non-angiomatous, non-myogenic)	Wittenberns et al. [132]	–	–	–	–	–	–	–	–	–	–	–
Squamous cell carcinoma	Nasal planum	Lascelles et al. [61]	–	–	–	–	–	–	–	–	–	–	–
	Dermal	Willcox et al. [131]	–	–	–	–	–	–	–	–	–	–	–
Synovial sarcoma	N/A	Vail et al. [124]	34	PFI	–	–	–	–	Yes	p < 0.001	–	–	–
Thymic tumors	Epithelial	Yale et al. [133]	30	PFI	–	–	–	–	–	–	p > 0.10	–	–

IPD, individual patient data; PFI, progression-free interval; RFI, recurrence-free interval; MFI, metastasis-free interval; N/A, not available; - , not available; NS, not significant ($p > 0.05$)) if actual p-value is not provided in the manuscript; HR, hazard ratio; ROC, Receiver-Operating-Characteristics curve; AUC, area under the ROC curve; TP, true positives (MC above cut-off and presence of outcome event); TN, true negatives (MC below cut-off and lack of outcome event); FP, false positives (MC above cut-off and lack of outcome event); FN, false negatives (MC below cut-off and presence of outcome event); Sen, sensitivity; Spe, specificity

Metastasis and Recurrence

Supplemental Table S7. Summary of the prognostic value of the mitotic count (MC) with regard to metastasis (Met) and recurrence (Rec) rate.

Tumor type/group	(Sub)type / location	Article	Number of cases / tumors with outcome	Met and/or Rec	IPD	Cut-off ranges	Rate of occurrence (%) or median interval (in days) of low MC cases	Rate of occurrence (%) or median interval (in days) of high MC cases	Kaplan-Meier curve	Log rank test	Cox proportional hazard regression model (univariate, unless stated otherwise)	ROC und AUC	Other statistical tests
Apocrine gland anal sac adenocarcinoma	N/A	Morello et al. [81]	–	–	–	–	–	–	–	–	–	–	–
	N/A	Pradel et al. [91]	39	Lymph node met	–	–	–	–	–	–	–	–	“not significantly associated”
	N/A	Schlag et al. [103]	107	Met	–	–	–	–	–	–	–	–	p = 0.90
	Early stage	Skorupski et al. [114]	20	Met, Rec	–	–	–	–	–	–	–	–	“not significantly associated”
	With lymph node metastasis	Tanis et al. [119]	–	–	–	–	–	–	–	–	–	–	–
Glial tumors	N/A	Merickel et al. [74]	–	–	–	–	–	–	–	–	–	–	–
Hemangio-sarcoma	Any other than skin	Ogilvie et al. [86]	–	–	–	–	–	–	–	–	–	–	–
	Nonvisceral: skin, eye, bone, muscle	Schultheiss [106]	–	–	–	–	–	–	–	–	–	–	–
	Cutaneous	Nobrega et al. [84]	–	–	–	–	–	–	–	–	–	–	–
	Splenic	Kim et al. [54]	–	–	–	–	–	–	–	–	–	–	–
	Splenic	Moore et al. [80]	–	–	–	–	–	–	–	–	–	–	–
	Subcutaneous & intramuscular	Shiu et al. [109]	–	–	–	–	–	–	–	–	–	–	–
	Falciform fat	Mendez et al. [73]	–	–	–	–	–	–	–	–	–	–	–
Insulinoma	N/A	Cleland et al. [23]	25	Recurrence of hypoglycemia	–	–	–	–	–	–	HR = 1.2 (1.1 – 1.3), p < 0.001	–	–
	N/A	Dunn et al. [32]	7	Recurrence of hypoglycaemic signs	Yes	–	–	–	–	–	–	–	–
Lymphoma	Burkitt-like	Aresu et al. [3]	–	–	–	–	–	–	–	–	–	–	–
	Multicentric	Dobson et al. [28]	43	Rec	–	–	–	–	–	–	HR: 1.006 (0.988-1-025), p = 0.502	–	–
	Multicentric	Kiupel et al. [56]	–	–	–	–	–	–	–	–	–	–	–
	Indolent	Flood-Knapik et al. [39]	–	–	–	–	–	–	–	–	–	–	–
	Diffuse small B-cell	Hughes et al. [51]	–	–	–	–	–	–	–	–	–	–	–

Tumor type/group	(Sub)type / location	Article	Number of cases / tumors with outcome	Met and/or Rec	IPD	Cut-off ranges	Rate of occurrence (%) or median interval (in days) of low MC cases	Rate of occurrence (%) or median interval (in days) of high MC cases	Kaplan-Meier curve	Log rank test	Cox proportional hazard regression model (univariate, unless stated otherwise)	ROC und AUC	Other statistical tests
	Diffuse large B-cell	Sierra Matiz et al. [110]	–	–	–	–	–	–	–	–	–	–	–
	Any	Valli et al. [125]	–	–	–	–	–	–	–	–	–	–	–
	Small intestinal	Yamazaki et al. [134]	–	–	–	–	–	–	–	–	–	–	–
Mammary tumors	Malignant	Canadas et al. [16]	–	–	–	–	–	–	–	–	–	–	–
	Malignant	Carvalho et al. [17] (2016)	–	–	–	–	–	–	–	–	–	–	–
	Malignant	Carvalho et al. [18] (2019)	–	–	–	–	–	–	–	–	–	–	–
	Malignant	Chen et al. [20]	–	–	–	–	–	–	–	–	–	–	–
	Malignant	Dutra et al. [33]	–	–	–	–	–	–	–	–	–	–	–
	Malignant	Liu et al. [64]	–	–	–	–	–	–	–	–	–	–	–
	Malignant	Ressel et al. [95]	–	–	–	–	–	–	–	–	–	–	–
	Malignant	Santos et al. [101] (2013)	85	Met and rec	–	0-9, 10-19, ≥20	0-9: 27.8% (10/36)	10-19: 24% (6/25)<br>≥20: 41.7% (10/24)	–	–	–	–	–
	Carcinoma	Santos et al. [102] (2013)	–	–	–	–	–	–	–	–	–	–	–
	Any	Dolka et al. [29] (2018)	57	Met (2 years post mastectomy)	–	<1, ≥1	8.3%	57.1%	–	–	–	–	Crude odds ratio: 14.67 (3.40 – 63.42), p < 0.001
						<2, ≥2	21.2%	50.0%	–	–	–	–	Crude odds ratio: 3.70 (0.89 – 15.35), p = 0.071
	Any	Dolka et al. [30] (2016)	–	–	–	–	–	–	–	–	–	–	–
	Any	Mainenti et al. [67]	–	–	–	–	–	–	–	–	–	–	–
	Any	Misdorp and Hart [76]	–	–	–	–	–	–	–	–	–	–	–
	Neuroendocrine Carcinoma	Nakagaki et al. [82]	4	Lymph node met	Yes	–	–	–	–	–	–	–	–
Mast cell tumor	Cutaneous	Berlato et al. [9] (2013)	–	–	–	–	–	–	–	–	–	–	–
	Cutaneous	Berlato et al. [8] (2018)	–	–	–	–	–	–	–	–	–	–	–
	Cutaneous	Elston et al. [36]	–	–	–	–	–	–	–	–	–	–	–
	Cutaneous	Gregorio et al. [45]	–	–	–	–	–	–	–	–	–	–	–
	Cutaneous	Krick et al. [58]	64	Lymph node met	–	–	–	–	–	–	–	–	Odds ratio: 1.0 (0.8 – 1.3), p = 0.8
	Cutaneous	Preziosi et al. [92]	–	–	–	–	–	–	–	–	–	–	–
	Cutaneous	Romansik et al. [98]	148	Rec	–	–	–	–	–	–	–	–	Wilcoxon-Mann-Whitney test: p = 0.97

Tumor type/group	(Sub)type / location	Article	Number of cases / tumors with outcome	Met and/or Rec	IPD	Cut-off ranges	Rate of occurrence (%) or median interval (in days) of low MC cases	Rate of occurrence (%) or median interval (in days) of high MC cases	Kaplan-Meier curve	Log rank test	Cox proportional hazard regression model (univariate, unless stated otherwise)	ROC und AUC	Other statistical tests
			148	Met	–	–	–	–	–	–	–	–	Wilcoxon-Mann-Whitney test: p < 0.0001
	Cutaneous	Skor et al. [113]	–	–	–	–	–	–	–	–	–	–	–
	Cutaneous	Thamm et al. [120]	–	–	–	–	–	–	–	–	–	–	–
	Cutaneous	Vascellari et al. [128]	–	–	–	–	–	–	–	–	–	–	–
	Cutaneous, multiple	O'Connell et al. [85]	–	–	–	–	–	–	–	–	–	–	–
	Cutaneous, Kiupel high grade, stage I	Moore et al. [79]	–	–	–	–	–	–	–	–	–	–	–
	Cutaneous and subcutaneous	Horta et al. [50]	–	–	–	–	–	–	–	–	–	–	–
	Cutaneous and subcutaneous	Knight et al. [57]	–	–	–	–	–	–	–	–	–	–	–
	Cutaneous and subcutaneous	Thompson et al. [121]	22	Rec	–	–	TN: 14<br>FN: 3<br>18%	TP: 2<br>FP: 3<br>40%	–	–	HR = 1.27 (1.00 – 1.61), p = 0.06	–	Sen: 40%<br>Spe: 82%
				Met	–	–	TN: 15<br>FN: 2<br>12%	TP: 5<br>FP: 0<br>100%	–	–	HR = 1.05 (1.01 – 1.091), p = 0.01		Sen: 71%<br>Spe: 100%
	Cutaneous and subcutaneous, in Pugs	McNiel et al. [70]	–	–	–	–	–	–	–	–	–	–	–
	Subcutaneous	Bostock et al. [12]	–	–	–	–	–	–	–	–	–	–	–
	Subcutaneous	Gill et al. [40]	–	–	–	–	–	–	–	–	–	–	–
	Subcutaneous	Newman et al. [83]	–	–	–	–	–	–	–	–	–	–	–
	Subcutaneous	Thompson et al. [122]	306	Rec	–	0, 1-4, >4	–	–	–	–	HR = 3.94 (1.19 – 13.10), p = 0.02;<br>HR = 44.23 (14.06 – 139.14), p < 0.01	–	–
						≤4, >4	–	–	–	–	HR = 11.22 (4.51 – 27-96), p < 0.01	–	–
				Met	–	0, 1-4, >4	–	–	–	–	HR = 2.96 (0.50 – 17.76), p = 0.23;<br>HR = 53.89 (10.92 – 265.87), p < 0.01	–	–
						≤4, >4	–	–	–	–	HR = 18.17 (4.54 – 72.77), p < 0.01	–	–
	Subcutaneous	Thompson et al. [123]	48 (subset from [122])	Rec	–	≤4, >4	TN: 21<br>FN: 12<br>38%	TP: 12<br>FP: 2<br>86%	–	–	–	–	Sen: 50%<br>Spe: 91%<br><br>Categorial MC values: Odds ratio = 6.00 (1.33 – 55.20), p = 0.01<br><br>Contiguous MC values: Odds ratio =

Tumor type/group	(Sub)type / location	Article	Number of cases / tumors with outcome	Met and/or Rec	IPD	Cut-off ranges	Rate of occurrence (%) or median interval (in days) of low MC cases	Rate of occurrence (%) or median interval (in days) of high MC cases	Kaplan-Meier curve	Log rank test	Cox proportional hazard regression model (univariate, unless stated otherwise)	ROC und AUC	Other statistical tests
													1.18 (1.03 – 1.40), p< 0.01
			24 (subset from [122])	Met	–	≤4, >4	TN: 12<br>FN: 5<br>29%	TP: 7<br>FP: 0<br>100%	–	–	–	–	Sen: 58%<br>Spe: 100%<br><br>Categorial MC values: Odds ratio = 9.61 (1.44 - ∞), p = 0.02<br><br>Contiguous MC values: Odds ratio = 1.79 (1.07 - ∞), p < 0.01
	Skin	Donnelly et al. [31]	90	Rec	–	–	–	–	–	–	–	–	Median MC values:<br>No recurrence: 0<br>Recurrence: 6.5<br><br>Mann-Whitney test: p < 0.001<br><br>Multivariate HR: NS (p > 0.05)
	Pinna	Schwab et al. [107]	28	Rec	–	≤5, >5	Not reached	5.5	–	p < 0.001	–	–	–
	Cutaneous & mucocutaneous, <12 months old	Rigas et al. [96]	16	Lymph node met	Yes	–	–	–	–	–	–	–	–
	High-risk	Pecceu et al. [88]	69	Met	–	≤5, >5	TN: 36<br>FN: 6<br>14%	TP: 8<br>FP: 19<br>30%	–	–	–	–	Sen: 57%<br>Spe: 65%
	Grade 3; cutaneous, subcutaneous & mucocutaneous	Hume et al. [52]	–	–	–	–	–	–	–	–	–	–	–
	Intramuscular	Robinson et al. [97]	–	–	–	–	–	–	–	–	–	–	–
	Oral mucosa	Elliott et al. [35]	–	–	–	–	–	–	–	–	–	–	–
	Not reported	Van Lelyveld et al. [126]	–	–	–	–	–	–	–	–	–	–	–
Melanocytic tumors / melanoma	Cutaneous	Laprie et al. [60]	68	Met and rec	–	–	TN: 49<br>FN: 4<br>8%	TP: 13<br>FP: 2<br>87%	–	–	–	–	Sen: 76%<br>Spe: 96%<br>Predictive value: 91% (62/68 cases accurately predicted)
	Cutaneous	Laver et al. [62]	–	–	–	–	–	–	–	–	–	–	–
	Cutaneous (exclusive paw pad)	Silvestri et al. [111]	–	–	–	–	–	–	–	–	–	–	–

Tumor type/group	(Sub)type / location	Article	Number of cases / tumors with outcome	Met and/or Rec	IPD	Cut-off ranges	Rate of occurrence (%) or median interval (in days) of low MC cases	Rate of occurrence (%) or median interval (in days) of high MC cases	Kaplan-Meier curve	Log rank test	Cox proportional hazard regression model (univariate, unless stated otherwise)	ROC und AUC	Other statistical tests
	Cutaneous and oral	Martinez et al. [68]	–	–	–	–	–	–	–	–	–	–	–
	Cutaneous and oral	Millanta et al. [75]	–	–	–	–	–	–	–	–	–	–	–
	Cutaneous and oral	Porcellato et al. [90]	–	–	–	–	–	–	–	–	–	–	–
	Skin, lip, oral	Bostock [11]	–	–	–	–	–	–	–	–	–	–	–
	Skin, lip, nail bed	Schultheiss [105]	–	–	–	–	–	–	–	–	–	–	–
	Intraocular	Giuliano et al. [42]	–	–	–	–	–	–	–	–	–	–	–
	Ocular (intraocular and scleral)	Wilcock and Peiffer [130]	61	–	–	<2, >4	TN: 52<br>FN: 0<br>0%	TP: 4<br>FP: 5<br>44%	–	–	–	–	Sen: 100%<br>Spe: 91%
	Oral	Bergin et al. [7]	–	–	–	–	–	–	–	–	–	–	–
	Oral	Baja et al. [5]	–	–	–	–	–	–	–	–	–	–	–
	Oral	Camerino et al. [15]	–	–	–	–	–	–	–	–	–	–	–
	Oral	Hahn et al. [47]	–	–	–	–	–	–	–	–	–	–	–
	Oral	Prouteau et al. [93]	–	–	–	–	–	–	–	–	–	–	–
	Oral	Ramos-Vara et al. [94]	–	–	–	–	–	–	–	–	–	–	–
	Oral	Vargas et al. [127]	–	–	–	–	–	–	–	–	–	–	–
	Oral	Wei et al. [129]	–	–	–	–	–	–	–	–	–	–	–
	Oral, osteo-cartilaginous differentiation	Sanchez et al. [100]	10	Rec	Yes	–	–	–	–	–	–	–	4 cases with highest MC had recurrence after 60 days,<br>3 cases with lowest MC had no recurrence
	Non-ocular (cutaneous and oral)	Spangler and Kass [116]	–	–	–	–	–	–	–	–	–	–	–
Esophageal sarcoma	Spirocera-induced	Pazzi et al. [87]	–	–	–	–	–	–	–	–	–	–	–
Osteo-sarcoma	Appendicular, small-breed dogs	Amsellem et al. [2]	–	–	–	–	–	–	–	–	–	–	–
	Appendicular	Guim et al. [46]	–	–	–	–	–	–	–	–	–	–	–
	Appendicular	Moore et al. [77]	–	–	–	–	–	–	–	–	–	–	–
	Appendicular	Saam et al. [99]	–	–	–	–	–	–	–	–	–	–	–
	Appendicular	Schott et al. [104]	85, three observers (A-C)	Met, 10 HPF	–	–	–	–	–	–	A: HR = 1.00 (0.99 – 1.01), p = 0.266	–	–
											B: HR = 1.01 (0.99 – 1.03), p = 0.109		
											C: HR = 1.00 (0.98 – 1.01), p = 0.979		
				Met, 3 HPF	–	–	–	–	–	–	A: HR = 1.02 (0.99 – 1.05), p = 0.114	–	–
											B: HR = 1.05 (1.00 – 1.09), p = 0.016		

Tumor type/group	(Sub)type / location	Article	Number of cases / tumors with outcome	Met and/or Rec	IPD	Cut-off ranges	Rate of occurrence (%) or median interval (in days) of low MC cases	Rate of occurrence (%) or median interval (in days) of high MC cases	Kaplan-Meier curve	Log rank test	Cox proportional hazard regression model (univariate, unless stated otherwise)	ROC und AUC	Other statistical tests
											C: HR = 1.01 (0.96 – 1.05), p = 0.750		
	Mandibular	Coyle et al. [25]	43	Met	–	≤20, >20	637 d	511 d	–	p = 0.815	–	–	–
					–	≤40, >40	697 d	201 d	–	p = 0.053	–	–	–
	Surface	Cook et al. [24]	–	–	–	–	–	–	–	–	–	–	–
	Any	Kirpensteijn et al. [55]	–	–	–	–	–	–	–	–	–	–	–
	Any	Loukopoulos et al. [65]	Unknown	Met	–	–	–	–	–	–	–	–	“significantly associated” (p > 0.05)
Pheochromocytoma	N/A	Zini et al. [135]	–	–	–	–	–	–	–	–	–	–	–
Pulmonary neoplasia	Carcinoma	McNiel et al. [69]	–	–	–	–	–	–	–	–	–	–	–
	Any primary	Able et al. [1]	–	–	–	–	–	–	–	–	–	–	–
	Any primary	McPhetridge [71]	–	–	–	–	–	–	–	–	–	–	–
Renal cell carcinoma	N/A	Carvalho et al. [19]	–	–	–	–	–	–	–	–	–	–	–
	N/A	Edmondson et al. [34]	–	–	–	–	–	–	–	–	–	–	–
Salivary gland neoplasia	N/A	Hammer et al. [48]	–	–	–	–	–	–	–	–	–	–	–
Sarcomas, non-osteogenic	Skin	Bray et al. [14]	350	Rec	–	0-9, 10-19, >19	–	–	–	–	–	–	Chi-squared test: x² = 2.8, p = 0.005<br><br>“distinguished dogs whose tumors did not recur >2 years after surgery”<br><br>Multivariate HR: NS (p > 0.05)
			54	Met	–	0-9, >10	–	–	–	–	–	–	p = 0.009
	Skin	Ettinger et al. [38]	54	Met	–	0-9, ≥10	–	–	–	–	–	–	p = 0.003
	Skin	Heller et al. [49]	87	Rec	–	≤9, ≥10	TN: 41<br>FN: 20<br>33%	TP: 12<br>FP: 14<br>46%	–	–	–	–	Krukal-Wallis test:<br>Contiguous values: p = 0.1456<br>Categorial values: p = 0.2393<br><br>Sen: 38%<br>Spe: 75%
				Met	–	≤9, ≥10	TN: 43<br>FN: 18<br>30%	TP: 9<br>FP: 17<br>35%	–	–	–	–	Krukal-Wallis test:<br>Contiguous values: p = 0.5393<br>Categorial values: p = 0.6393

Tumor type/group	(Sub)type / location	Article	Number of cases / tumors with outcome	Met and/or Rec	IPD	Cut-off ranges	Rate of occurrence (%) or median interval (in days) of low MC cases	Rate of occurrence (%) or median interval (in days) of high MC cases	Kaplan-Meier curve	Log rank test	Cox proportional hazard regression model (univariate, unless stated otherwise)	ROC und AUC	Other statistical tests
													Sen: 33%<br>Spe: 72%
	Skin	Kuntz et al. [59]	75	Met	–	<10, 10-19, >19 resp. <20, ≥20			–	–	"significant"	–	Multivariate HR: ≥20 was 5x more likely for TRM than <20 (95%CI: 0.075 – 0.594)
	Skin (extraoral)	Simon et al. [112]	27	Rec	–	0-9, 10-19, ≥20	0-9: 3/22 (14%)	10-19: 2/2 (100%)<br>≥20: 2/3 (66%)	–	–	–	–	Chi-squared test: p = 0.017
				Met	–	0-9, 10-19, ≥20	–	–	–	–	–	–	Chi-squared test: NS (p > 0.05)
	(Sub)cutaneous, with recurrence	Chiti et al. [22]	23	Rec	Yes	–	–	–	–	–	–	–	–
	Subcutaneous	McSporran [72]	86	Rec	–	0-5, 6-9, >9	–	–	–	p = 0.002; 0-5 vs. 6-9: p > 0.05; 0-5 vs. >9: p = 0.0004; 6-9 vs. >9: p > 0.05	–	–	–
	Skin	Bostock and Dye [13]	160	Rec	–	0-8, ≥9	30/120 (25%)	25/40 (63%)	–	–	–	–	p < 0.025
				Met	–	0-8, ≥9	2/120 (1.6%)	6/40 (15%)	–	–	–	–	p < 0.05
	High grade (MC ≥9)	Crownshaw et al. [26]	–	–	–	–	–	–	–	–	–	–	–
	High grade, any location	Selting et al. [108]	39	Rec	–	<20, ≥20	–	–	–	p = 0.732	–	–	–
						<20, 20-49, ≥50	–	–	–	p = 0.937	–	–	–
				Met	–	<20, ≥20	216 d	503 d	–	p = 0.345	–	–	–
						<20, 20-49, ≥50	216 d	724 d<br>N/A	–	p = 0.094	–	–	–
	Abdominal visceral	Linden et al. [63]	–	–	–	–	–	–	–	–	–	–	–
	Gastrointestinal sarcomas	Del Alcazar [27]	43	Met	–	–	–	–	–	–	–	–	≤9: OR: 0.19 (0.038-0.93), p = 0.04
	Gastrointestinal stromal tumors	Berger et al. [6]	–	–	–	–	–	–	–	–	–	–	–

Tumor type/group	(Sub)type / location	Article	Number of cases / tumors with outcome	Met and/or Rec	IPD	Cut-off ranges	Rate of occurrence (%) or median interval (in days) of low MC cases	Rate of occurrence (%) or median interval (in days) of high MC cases	Kaplan-Meier curve	Log rank test	Cox proportional hazard regression model (univariate, unless stated otherwise)	ROC und AUC	Other statistical tests
	Gastrointestinal stromal tumors	Gillespie et al. [41]	–	–	–	–	–	–	–	–	–	–	–
	Hemangiopericytoma, skin	Graves et al. [44]	21	Rec	–	–	–	–	–	–	–	–	Without recurrence: Mean MC: 4.7<br>With recurrence Mean MC: 2.8<br>One-way analysis of variance: NS (p > 0.05)
	Myxosarcoma, cutaneous	Iwaki et al. [53]	21	Rec	–	<10, 10-19, >19	<10: 4/20 (20%)	10-19: 3/3 (100%)<br>>19: 4/6 (67%)	–	–	–	–	–
						<10, ≥10	339 d	119 d	–	–	–	–	–
				Met	–	<10, 10-19, >19	<10: 3/20 (15%)	10-19: 2/3 (66%)<br>>19: 3/6 (50%)	–	–	–	–	–
	Peripheral nerve sheath tumor, skin	Boos et al. [10]	54	Rec	–	<1, 1, >2	<1: 2/18 (11%)	1: 9/27 (33%)<br>>2: 6/9 (67%)	–	–	–	–	–
	Perivascular wall tumor, cutaneous	Chiti et al. [21]	102	RFI	–	–	–	–	–	–	HR = 1.085 (1.051 – 1.12), p < 0.001	–	–
	Perivascular wall tumor, cutaneous	Stefanello et al. [118]	53	Rec and met	–	–	–	–	–	–	HR: 1.001 (0.95 – 1.06), Wald test: 0.017, p = 0.9	–	–
	Smooth muscle tumors, any location	Avallone et al. [4]	25	Met	–	–	–	–	–	–	–	–	“correlated”
	Intestinal sarcomas	Maas et al. [66]	–	–	–	–	–	–	–	–	–	–	–
Splenic tumors (exclusive hemangiosarcoma)	“Fibrohistiocytic nodules”	Moore et al. [78]	–	–	–	–	–	–	–	–	–	–	–
	“Fibrohistiocytic nodules”	Spangler and Kass [117]	–	–	–	–	–	–	–	–	–	–	–
	Mesenchymal (non-angiomatous, non-lymphomatous)	Spangler et al. [115]	–	–	–	–	–	–	–	–	–	–	–
	Stromal sarcoma (non-angiomatous, non-myogenic)	Wittenberns et al. [132]	–	–	–	–	–	–	–	–	–	–	–
Squamous cell carcinoma	Nasal planum	Lascelles et al. [61]	12	Rec	–	–	–	–	–	–	–	–	“no apparent relationship”
	Dermal	Willcox et al. [131]	–	–	–	–	–	–	–	–	–	–	–

Tumor type/group	(Sub)type / location	Article	Number of cases / tumors with outcome	Met and/or Rec	IPD	Cut-off ranges	Rate of occurrence (%) or median interval (in days) of low MC cases	Rate of occurrence (%) or median interval (in days) of high MC cases	Kaplan-Meier curve	Log rank test	Cox proportional hazard regression model (univariate, unless stated otherwise)	ROC und AUC	Other statistical tests
Synovial sarcoma	N/A	Vail et al. [124]	–	–	–	–	–	–	–	–	–	–	–
Thymic tumors	Epithelial	Yale et al. [133]	30	Met, rec	–	–	–	–	–	–	–	–	“not associated”

Met, metastasis; Rec, recurrence; HR, hazard ratio; ROC, Receiver-Operating-Characteristics curve; AUC, area under the ROC curve; TP, true positives (MC above cut-off and presence of outcome event); TN, true negatives (MC below cut-off and lack of outcome event); FP, false positives (MC above cut-off and lack of outcome event); FN, false negatives (MC below cut-off and presence of outcome event); Sen, sensitivity; Spe, specificity; N/A, not available; - , not available; HR, hazard ratio; OR, odds ratio; NS, not significant ($p > 0.05$) if actual p-value is not provided in the manuscript

References

1. Able H, Wolf-Ringwall A, Rendahl A, et al. Computed tomography radiomic features hold prognostic utility for canine lung tumors: An analytical study. *PloS one*. 2021;16**:** e0256139. doi: 10.1371/journal.pone.0256139
2. Amsellem PM, Selmic LE, Wypij JM, et al. Appendicular osteosarcoma in small-breed dogs: 51 cases (1986-2011). *J Am Vet Med Assoc*. 2014;245**:** 203-210. doi: 10.2460/javma.245.2.203
3. Aresu L, Agnoli C, Nicoletti A, et al. Phenotypical Characterization and Clinical Outcome of Canine Burkitt-Like Lymphoma. *Front Vet Sci*. 2021;8**:** 647009. doi: 10.3389/fvets.2021.647009
4. Avallone G, Pellegrino V, Muscatello LV, et al. Canine smooth muscle tumors: A clinicopathological study. *Vet Pathol*. 2022;59**:** 244-255. doi: 10.1177/03009858211066862
5. Baja AJ, Kelsey KL, Rusl, er DM, Gieger TL, Nolan MW. A retrospective study of 101 dogs with oral melanoma treated with a weekly or biweekly 6 Gy × 6 radiotherapy protocol. *Vet Comp Oncol*. 2022. doi: 10.1111/vco.12815
6. Berger EP, Johannes CM, Jergens AE, et al. Retrospective evaluation of toceranib phosphate (Palladia®) use in the treatment of gastrointestinal stromal tumors of dogs. *J Vet Intern Med*. 2018;32**:** 2045-2053. doi: 10.1111/jvim.15335
7. Bergin IL, Smedley RC, Esplin DG, Spangler WL, Kiupel M. Prognostic evaluation of Ki67 threshold value in canine oral melanoma. *Vet Pathol*. 2011;48**:** 41-53. doi: 10.1177/0300985810388947
8. Berlato D, Murphy S, Laberke S, Rasotto R. Comparison of minichromosome maintenance protein 7, Ki67 and mitotic index in the prognosis of intermediate Patnaik grade cutaneous mast cell tumours in dogs. *Vet Comp Oncol*. 2018;16**:** 535-543. doi: 10.1111/vco.12412
9. Berlato D, Murphy S, Monti P, et al. Comparison of mitotic index and Ki67 index in the prognostication of canine cutaneous mast cell tumours. *Vet Comp Oncol*. 2015;13**:** 143-150. doi: 10.1111/vco.12029
10. Boos GS, Bassuino DM, Wurster F, et al. Retrospective canine skin peripheral nerve sheath tumors data with emphasis on histologic, immunohistochemical and prognostic factors. *Pesquisa Veterinaria Brasileira*. 2015;35**:** 965-974. doi: 10.1590/S0100-736X2015001200005
11. Bostock DE. Prognosis after surgical excision of canine melanomas. *Vet Pathol*. 1979;16**:** 32-40.
12. Bostock DE, Crocker J, Harris K, Smith P. Nucleolar organiser regions as indicators of post-surgical prognosis in canine spontaneous mast cell tumours. *Br J Cancer*. 1989;59**:** 915-918. doi: 10.1038/bjc.1989.193
13. Bostock DE, Dye MT. Prognosis after surgical excision of canine fibrous connective tissue sarcomas. *Vet Pathol*. 1980;17**:** 581-588. doi: 10.1177/030098588001700507
14. Bray JP, Polton GA, McSporran KD, Bridges J, Whitbread TM. Canine soft tissue sarcoma managed in first opinion practice: outcome in 350 cases. *Vet Surg*. 2014;43**:** 774-782. doi: 10.1111/j.1532-950X.2014.12185.x
15. Camerino M, Giacobino D, Manassero L, et al. Prognostic impact of bone invasion in canine oral malignant melanoma treated by surgery and anti-CSPG4 vaccination: A retrospective study on 68 cases (2010-2020). *Vet Comp Oncol*. 2022;20**:** 189-197. doi: 10.1111/vco.12761
16. Canadas A, França M, Pereira C, et al. Canine Mammary Tumors: Comparison of Classification and Grading Methods in a Survival Study. *Vet Pathol*. 2019;56**:** 208-219. doi: 10.1177/0300985818806968

17. Carvalho MI, Pires I, Prada J, Lobo L, Queiroga FL. Ki-67 and PCNA Expression in Canine Mammary Tumors and Adjacent Nonneoplastic Mammary Glands: Prognostic Impact by a Multivariate Survival Analysis. *Vet Pathol*. 2016;53**:** 1138-1146. doi: 10.1177/0300985816646429
18. Carvalho MI, Pires I, Prada J, et al. Assessing the interleukin 35 immunoexpression in malignant canine mammary tumors: Association with clinicopathological parameters and prognosis. *Anticancer Research*. 2019;39**:** 2077-2083. doi: 10.21873/anticanres.13319
19. Carvalho S, Stoll AL, Priestnall SL, et al. Retrospective evaluation of COX-2 expression, histological and clinical factors as prognostic indicators in dogs with renal cell carcinomas undergoing nephrectomy. *Vet Comp Oncol*. 2017;15**:** 1280-1294. doi: 10.1111/vco.12264
20. Chen YC, Chen YY, Liao JW, Chang SC. Expression and prognostic value of c-met in canine mammary tumours. *Vet Comp Oncol*. 2018;16**:** 670-676. doi: 10.1111/vco.12439
21. Chiti LE, Ferrari R, Boracchi P, et al. Prognostic impact of clinical, haematological, and histopathological variables in 102 canine cutaneous perivascular wall tumours. *Vet Comp Oncol*. 2021;19**:** 275-283. doi: 10.1111/vco.12673
22. Chiti LE, Ferrari R, Roccabianca P, et al. Surgical Margins in Canine Cutaneous Soft-Tissue Sarcomas: A Dichotomous Classification System Does Not Accurately Predict the Risk of Local Recurrence. *Animals (Basel)*. 2021;11. doi: 10.3390/ani11082367
23. Cleland NT, Morton J, Delisser PJ. Outcome after surgical management of canine insulinoma in 49 cases. *Vet Comp Oncol*. 2021;19**:** 428-441. doi: 10.1111/vco.12628
24. Cook MR, Lorbach J, Husb, et al. A retrospective analysis of 11 dogs with surface osteosarcoma. *Vet Comp Oncol*. 2022;20**:** 82-90. doi: 10.1111/vco.12741
25. Coyle VJ, Rassnick KM, Borst LB, et al. Biological behaviour of canine mandibular osteosarcoma. A retrospective study of 50 cases (1999-2007). *Vet Comp Oncol*. 2015;13**:** 89-97. doi: doi: 10.1111/vco.12020
26. Crownshaw AH, McEntee MC, Nolan MW, Gieger TL. Evaluation of variables associated with outcomes in 41 dogs with incompletely excised high-grade soft tissue sarcomas treated with definitive-intent radiation therapy with or without chemotherapy. *J Am Vet Med Assoc*. 2020;256**:** 783-791. doi: 10.2460/javma.256.7.783
27. Del Alcazar CM, Mahoney JA, Dittrich K, Stefanovski D, Church ME. Outcome, prognostic factors and histological characterization of canine gastrointestinal sarcomas. *Vet Comp Oncol*. 2021;19**:** 578-586. doi: 10.1111/vco.12696
28. Dobson JM, Blackwood LB, McInnes EF, et al. Prognostic variables in canine multicentric lymphosarcoma. *J Small Anim Pract*. 2001;42**:** 377-384. doi: doi: 10.1111/j.1748-5827.2001.tb02485.x
29. Dolka I, Czopowicz M, Gruk-Jurka A, Wojtkowska A, Sapierzyński R, Jurka P. Diagnostic efficacy of smear cytology and Robinson's cytological grading of canine mammary tumors with respect to histopathology, cytomorphometry, metastases and overall survival. *PloS one*. 2018;13**:** e0191595. doi: 10.1371/journal.pone.0191595
30. Dolka I, Król M, Sapierzyński R. Evaluation of apoptosis-associated protein (Bcl-2, Bax, cleaved caspase-3 and p53) expression in canine mammary tumors: An immunohistochemical and prognostic study. *Res Vet Sci*. 2016;105**:** 124-133. doi: 10.1016/j.rvsc.2016.02.004
31. Donnelly L, Mullin C, Balko J, et al. Evaluation of histological grade and histologically tumour-free margins as predictors of local recurrence in completely excised canine mast cell tumours. *Vet Comp Oncol*. 2015;13**:** 70-76. doi: 10.1111/vco.12021
32. Dunn JK, Bostock DE, Herrtage ME, Jackson KF, Walker MJ. Insulin-secreting tumours of the canine pancreas: Clinical and pathological features of 11 cases. *Journal of Small Animal Practice*. 1993;34**:** 325-331. doi: 10.1111/j.1748-5827.1993.tb02704.x

33. Dutra AP, Azevedo Júnior GM, Schmitt FC, Cassali GD. Assessment of cell proliferation and prognostic factors in canine mammary gland tumors. *Arquivo Brasileiro de Medicina Veterinária e Zootecnia*. 2008;60**:** 1403-1412.
34. Edmondson EF, Hess AM, Powers BE. Prognostic significance of histologic features in canine renal cell carcinomas: 70 nephrectomies. *Vet Pathol*. 2015;52**:** 260-268. doi: 10.1177/0300985814533803
35. Elliott JW, Cripps P, Blackwood L, Berlato D, Murphy S, Grant IA. Canine oral mucosal mast cell tumours. *Vet Comp Oncol*. 2016;14**:** 101-111. doi: 10.1111/vco.12071
36. Elston LB, Sueiro FA, Cavalcanti JN, Metze K. The importance of the mitotic index as a prognostic factor for survival of canine cutaneous mast cell tumors: a validation study. *Vet Pathol*. 2009;46**:** 362-364, author reply 364-365. doi: 10.1354/vp.46-2-362
37. Esplin DG. Survival of dogs following surgical excision of histologically well-differentiated melanocytic neoplasms of the mucous membranes of the lips and oral cavity. *Vet Pathol*. 2008;45**:** 889-896. 10.1354/vp.45-6-889
38. Ettinger SN, Scase TJ, Oberthaler KT, et al. Association of argyrophilic nucleolar organizing regions, Ki-67, and proliferating cell nuclear antigen scores with histologic grade and survival in dogs with soft tissue sarcomas: 60 Cases (1996-2002). *Journal of the American Veterinary Medical Association*. 2006;228**:** 1053-1062. doi: 10.2460/javma.228.7.1053
39. Flood-Knapik KE, Durham AC, Gregor TP, Sánchez MD, Durney ME, Sorenmo KU. Clinical, histopathological and immunohistochemical characterization of canine indolent lymphoma. *Vet Comp Oncol*. 2013;11**:** 272-286. doi: 10.1111/j.1476-5829.2011.00317.x
40. Gill V, Leibman N, Monette S, Craft DM, Bergman PJ. Prognostic Indicators and Clinical Outcome in Dogs with Subcutaneous Mast Cell Tumors Treated with Surgery Alone: 43 Cases. *J Am Anim Hosp Assoc*. 2020;56**:** 215-225. doi: 10.5326/JAAHA-MS-6960
41. Gillespie V, Baer K, Farrelly J, Craft D, Luong R. Canine gastrointestinal stromal tumors: immunohistochemical expression of CD34 and examination of prognostic indicators including proliferation markers Ki67 and AgNOR. *Vet Pathol*. 2011;48**:** 283-291. doi: 10.1177/0300985810380397
42. Giuliano EA, Chappell R, Fischer B, Dubielzig RR. A matched observational study of canine survival with primary intraocular melanocytic neoplasia. *Veterinary Ophthalmology*. 1999;2**:** 185-190. doi: 10.1046/j.1463-5224.1999.00080.x
43. Goldschmidt M, Peña L, Rasotto R, Zappulli V. Classification and grading of canine mammary tumors. *Vet Pathol*. 2011;48**:** 117-131. 10.1177/0300985810393258
44. Graves GM, Bjorling DE, Mahaffey E. Canine hemangiopericytoma: 23 cases (1967-1984). *J Am Vet Med Assoc*. 1988;192**:** 99-102.
45. Gregório H, Raposo T, Queiroga FL, Pires I, Pena L, Prada J. High COX-2 expression in canine mast cell tumours is associated with proliferation, angiogenesis and decreased overall survival. *Veterinary and comparative oncology*. 2017;15**:** 1382-1392. doi: 10.1111/vco.12280
46. Guim TN, Bianchi MV, De Lorenzo C, et al. Relationship Between Clinicopathological Features and Prognosis in Appendicular Osteosarcoma in Dogs. *Journal of Comparative Pathology*. 2020;180**:** 91-99. doi: 10.1016/j.jcpa.2020.09.003
47. Hahn KA, DeNicola DB, Richardson RC, Hahn EA. Canine oral malignant melanoma: Prognostic utility of an alternative staging system. *Journal of Small Animal Practice*. 1994;35**:** 251-256.
48. Hammer A, Getzy D, Ogilvie G, Upton M, Klausner J, Kisseberth WC. Salivary gland neoplasia in the dog and cat: survival times and prognostic factors. *J Am Anim Hosp Assoc*. 2001;37**:** 478-482. doi: 10.5326/15473317-37-5-478
49. Heller DA, Stebbins ME, Reynolds TL, Hauck ML. A retrospective study of 87 cases of canine soft tissue sarcomas. *Int J Appl Res Vet Med*. 2005;3**:** 81-87.

50. Horta RS, Lavalle GE, Monteiro LN, Souza MCC, Cassali GD, Araújo RB. Assessment of Canine Mast Cell Tumor Mortality Risk Based on Clinical, Histologic, Immunohistochemical, and Molecular Features. *Vet Pathol*. 2018;55**:** 212-223. doi: 10.1177/0300985817747325
51. Hughes KL, Ehrhart EJ, Rout ED, et al. Diffuse Small B-Cell Lymphoma: A High-Grade Malignancy. *Vet Pathol*. 2021;58**:** 912-922. doi: 10.1177/0300985820985221
52. Hume CT, Kiupel M, Rigatti L, Shofer FS, Skorupski KA, Sorenmo KU. Outcomes of dogs with grade 3 mast cell tumors: 43 cases (1997-2007). *Journal of the American Animal Hospital Association*. 2011;47**:** 37-44. doi: 10.5326/JAAHA-MS-5557
53. Iwaki Y, Lindley S, Smith A, Curran KM, Looper J. Canine myxosarcomas, a retrospective analysis of 32 dogs (2003-2018). *BMC Veterinary Research*. 2019;15. doi: 10.1186/s12917-019-1956-z
54. Kim SE, Liptak JM, Gall TT, Monteith GJ, Woods JP. Epirubicin in the adjuvant treatment of splenic hemangiosarcoma in dogs: 59 cases (1997-2004). *J Am Vet Med Assoc*. 2007;231**:** 1550-1557. doi: 10.2460/javma.231.10.1550
55. Kirpensteijn J, Kik M, Rutteman GR, Teske E. Prognostic significance of a new histologic grading system for canine osteosarcoma. *Vet Pathol*. 2002;39**:** 240-246. doi: 10.1354/vp.39-2-240
56. Kiupel M, Teske E, Bostock D. Prognostic factors for treated canine malignant lymphoma. *Vet Pathol*. 1999;36**:** 292-300.
57. Knight BJ, Wood GA, Foster RA, Coomber BL. Beclin-1 is a novel predictive biomarker for canine cutaneous and subcutaneous mast cell tumors. *Vet Pathol*. 2022;59**:** 46-56. doi: 10.1177/03009858211042578
58. Krick EL, Kiupel M, Durham AC, Thaiwong T, Brown DC, Sorenmo KU. Investigating associations between proliferation indices, c-kit, and lymph node stage in canine mast cell tumors. *Journal of the American Animal Hospital Association*. 2017;53**:** 258-264. doi: 10.5326/JAAHA-MS-6265
59. Kuntz CA, Dernell WS, Powers BE, Devitt C, Straw RC, Withrow SJ. Prognostic factors for surgical treatment of soft-tissue sarcomas in dogs: 75 cases (1986-1996). *J Am Vet Med Assoc*. 1997;211**:** 1147-1151.
60. Laprie C, Abadie J, Amardeilh MF, Net JL, Lagadic M, Delverdier M. MIB-1 immunoreactivity correlates with biologic behaviour in canine cutaneous melanoma. *Vet Dermatol*. 2001;12**:** 139-147. doi: 10.1046/j.1365-3164.2001.00236.x
61. Lascelles BD, Parry AT, Stidworthy MF, Dobson JM, White RA. Squamous cell carcinoma of the nasal planum in 17 dogs. *Vet Rec*. 2000;147**:** 473-476. doi: 10.1136/vr.147.17.473
62. Laver T, Feldhaeusser BR, Robat CS, et al. Post-surgical outcome and prognostic factors in canine malignant melanomas of the haired skin: 87 cases (2003-2015). *Canadian Veterinary Journal*. 2018;59**:** 981-987.
63. Linden D, Liptak JM, Vinayak A, et al. Outcomes and prognostic variables associated with primary abdominal visceral soft tissue sarcomas in dogs: A Veterinary Society of Surgical Oncology retrospective study. *Veterinary and comparative oncology*. 2019;17**:** 265-270. doi: 10.1111/vco.12456
64. Liu JL, Chang KC, Lo CC, Chu PY, Liu CH. Expression of autophagy-related protein Beclin-1 in malignant canine mammary tumors. *BMC Vet Res*. 2013;9**:** 75. doi: 10.1186/1746-6148-9-75
65. Loukopoulos P, Robinson WF. Clinicopathological relevance of tumour grading in canine osteosarcoma. *J Comp Pathol*. 2007;136**:** 65-73. doi: 10.1016/j.jcpa.2006.11.005
66. Maas CP, ter Haar G, van der Gaag I, Kirpensteijn J. Reclassification of small intestinal and cecal smooth muscle tumors in 72 dogs: clinical, histologic, and immunohistochemical evaluation. *Vet Surg*. 2007;36**:** 302-313. doi: 10.1111/j.1532-950X.2007.00271.x

67. Mainenti M, Rasotto R, Carnier P, Zappulli V. Oestrogen and progesterone receptor expression in subtypes of canine mammary tumours in intact and ovariectomised dogs. *Vet J*. 2014;202**:** 62-68. doi: 10.1016/j.tvjl.2014.06.003
68. Martínez CM, Peñafiel-Verdú C, Vilafranca M, et al. Cyclooxygenase-2 expression is related with localization, proliferation, and overall survival in canine melanocytic neoplasms. *Vet Pathol*. 2011;48**:** 1204-1211. doi: 10.1177/0300985810396517
69. McNiel EA, Ogilvie GK, Powers BE, Hutchison JM, Salman MD, Withrow SJ. Evaluation of prognostic factors for dogs with primary lung tumors: 67 cases (1985-1992). *J Am Vet Med Assoc*. 1997;211**:** 1422-1427.
70. McNiel EA, Prink AL, O'Brien TD. Evaluation of risk and clinical outcome of mast cell tumours in pug dogs. *Vet Comp Oncol*. 2006;4**:** 2-8. doi: 10.1111/j.1476-5810.2006.00085.x
71. McPhetridge JB, Scharf VF, Regier PJ, et al. Distribution of histopathologic types of primary pulmonary neoplasia in dogs and outcome of affected dogs: 340 cases (2010-2019). *J Am Vet Med Assoc*. 2021;260**:** 234-243. doi: 10.2460/javma.20.12.0698
72. McSporran KD. Histologic grade predicts recurrence for marginally excised canine subcutaneous soft tissue sarcomas. *Vet Pathol*. 2009;46**:** 928-933. doi: 10.1354/vp.08-VP-0277-M-FL
73. Mendez SE, Sykes Crumplar SE, Durham AC. Primary Hemangiosarcoma of the Falciform Fat in Seven Dogs (2007-2015). *J Am Anim Hosp Assoc*. 2020;56**:** 120-126. doi: 10.5326/jaaha-ms-6967
74. Merickel JL, Pluhar GE, Rendahl A, O'Sullivan MG. Prognostic histopathologic features of canine glial tumors. *Vet Pathol*. 2021;58**:** 945-951. doi: 10.1177/03009858211025795
75. Millanta F, Fratini F, Corazza M, Castagnaro M, Zappulli V, Poli A. Proliferation activity in oral and cutaneous canine melanocytic tumours: correlation with histological parameters, location, and clinical behaviour. *Res Vet Sci*. 2002;73**:** 45-51. doi: 10.1016/s0034-5288(02)00041-3
76. Misdorp W, Hart AA. Prognostic factors in canine mammary cancer. *J Natl Cancer Inst*. 1976;56**:** 779-786. doi: 10.1093/jnci/56.4.779
77. Moore AS, Dernell WS, Ogilvie GK, et al. Doxorubicin and BAY 12-9566 for the treatment of osteosarcoma in dogs: a randomized, double-blind, placebo-controlled study. *J Vet Intern Med*. 2007;21**:** 783-790. doi: 10.1892/0891-6640(2007)21[783:dabftt]2.0.co;2
78. Moore AS, Frimberger AE, Sullivan N, Moore PF. Histologic and immunohistochemical review of splenic fibrohistiocytic nodules in dogs. *J Vet Intern Med*. 2012;26**:** 1164-1168. doi: 10.1111/j.1939-1676.2012.00986.x
79. Moore AS, Frimberger AE, Taylor D, Sullivan N. Retrospective outcome evaluation for dogs with surgically excised, solitary Kiupel high-grade, cutaneous mast cell tumours. *Vet Comp Oncol*. 2020;18**:** 402-408. doi: 10.1111/vco.12565
80. Moore AS, Rassnick KM, Frimberger AE. Evaluation of clinical and histologic factors associated with survival time in dogs with stage II splenic hemangiosarcoma treated by splenectomy and adjuvant chemotherapy: 30 cases (2011-2014). *J Am Vet Med Assoc*. 2017;251**:** 559-565. doi: 10.2460/javma.251.5.559
81. Morello EM, Cino M, Giacobino D, et al. Prognostic value of ki67 and other clinical and histopathological factors in canine apocrine gland anal sac adenocarcinoma. *Animals*. 2021;11. doi: 10.3390/ani11061649
82. Nakagaki KYR, Nunes MM, Garcia APV, De Brot M, Cassali GD. Neuroendocrine Carcinomas of the Canine Mammary Gland: Histopathological and Immunohistochemical Characteristics. *Front Vet Sci*. 2020;7**:** 621714. doi: 10.3389/fvets.2020.621714

83. Newman SJ, Mrkonjich L, Walker KK, Rohrbach BW. Canine subcutaneous mast cell tumour: diagnosis and prognosis. *J Comp Pathol*. 2007;136**:** 231-239. doi: 10.1016/j.jcpa.2007.02.003
84. Nóbrega DF, Sehaber VF, Madureira R, Bracarense APFRL. Canine Cutaneous Haemangiosarcoma: Biomarkers and Survival. *Journal of Comparative Pathology*. 2019;166**:** 87-96. doi: 10.1016/j.jcpa.2018.10.181
85. O'Connell K, Thomson M. Evaluation of prognostic indicators in dogs with multiple, simultaneously occurring cutaneous mast cell tumours: 63 cases. *Vet Comp Oncol*. 2013;11**:** 51-62. doi: 10.1111/j.1476-5829.2011.00301.x
86. Ogilvie GK, Powers BE, Mallinckrodt CH, Withrow SJ. Surgery and doxorubicin in dogs with hemangiosarcoma. *J Vet Intern Med*. 1996;10**:** 379-384. doi: 10.1111/j.1939-1676.1996.tb02085.x
87. Pazzi P, Kavkovsky A, Shipov A, Segev G, Dvir E. Spirocerca lupi induced oesophageal neoplasia: Predictors of surgical outcome. *Vet Parasitol*. 2018;250**:** 71-77. doi: 10.1016/j.vetpar.2017.11.013
88. Pecceu E, Serra Varela JC, Handel I, Piccinelli C, Milne E, Lawrence J. Ultrasound is a poor predictor of early or overt liver or spleen metastasis in dogs with high-risk mast cell tumours. *Vet Comp Oncol*. 2020;18**:** 389-401. doi: 10.1111/vco.12563
89. Peña L, De Andrés PJ, Clemente M, Cuesta P, Pérez-Alenza MD. Prognostic value of histological grading in noninflammatory canine mammary carcinomas in a prospective study with two-year follow-up: relationship with clinical and histological characteristics. *Vet Pathol*. 2013;50**:** 94-105. 10.1177/0300985812447830
90. Porcellato I, Brachelente C, Cappelli K, et al. FoxP3, CTLA-4, and IDO in Canine Melanocytic Tumors. *Vet Pathol*. 2021;58**:** 42-52. doi: 10.1177/0300985820960131
91. Pradel J, Berlato D, Dobromylskyj M, Rasotto R. Prognostic significance of histopathology in canine anal sac gland adenocarcinomas: Preliminary results in a retrospective study of 39 cases. *Vet Comp Oncol*. 2018;16**:** 518-528. doi: 10.1111/vco.12410
92. Preziosi R, Sarli G, Paltrinieri M. Prognostic value of intratumoral vessel density in cutaneous mast cell tumors of the dog. *J Comp Pathol*. 2004;130**:** 143-151. doi: 10.1016/j.jcpa.2003.10.003
93. Prouteau A, Chocteau F, de Brito C, et al. Prognostic value of somatic focal amplifications on chromosome 30 in canine oral melanoma. *Vet Comp Oncol*. 2020;18**:** 214-223. doi: 10.1111/vco.12536
94. Ramos-Vara JA, Beissenherz ME, Miller MA, et al. Retrospective study of 338 canine oral melanomas with clinical, histologic, and immunohistochemical review of 129 cases. *Vet Pathol*. 2000;37**:** 597-608. doi: 10.1354/vp.37-6-597
95. Ressel L, Puleio R, Loria GR, et al. HER-2 expression in canine morphologically normal, hyperplastic and neoplastic mammary tissues and its correlation with the clinical outcome. *Res Vet Sci*. 2013;94**:** 299-305. doi: 10.1016/j.rvsc.2012.09.016
96. Rigas K, Biasoli D, Polton G, et al. Mast cell tumours in dogs less than 12 months of age: a multi-institutional retrospective study. *J Small Anim Pract*. 2020;61**:** 449-457. doi: 10.1111/jsap.13181
97. Robinson WP, Elliott J, Baines SJ, Owen L, Shales CJ. Intramuscular mast cell tumors in 7 dogs. *Can Vet J*. 2017;58**:** 931-935.
98. Romansik EM, Reilly CM, Kass PH, Moore PF, London CA. Mitotic index is predictive for survival for canine cutaneous mast cell tumors. *Vet Pathol*. 2007;44**:** 335-341. doi: 10.1354/vp.44-3-335

99. Saam DE, Liptak JM, Stalker MJ, Chun R. Predictors of outcome in dogs treated with adjuvant carboplatin for appendicular osteosarcoma: 65 cases (1996-2006). *J Am Vet Med Assoc*. 2011;238**:** 195-206. doi: 10.2460/javma.238.2.195
100. Sánchez J, Ramirez GA, Buendia AJ, et al. Immunohistochemical characterization and evaluation of prognostic factors in canine oral melanomas with osteocartilaginous differentiation. *Vet Pathol*. 2007;44**:** 676-682. doi: 10.1354/vp.44-5-676
101. Santos AA, Lopes CC, Ribeiro JR, et al. Identification of prognostic factors in canine mammary malignant tumours: a multivariable survival study. *BMC Vet Res*. 2013;9**:** 1. doi: 10.1186/1746-6148-9-1
102. Santos M, Correia-Gomes C, Marcos R, et al. Value of the Nottingham Histological Grading Parameters and Nottingham Prognostic Index in Canine Mammary Carcinoma. *Anticancer Res*. 2015;35**:** 4219-4227.
103. Schlag AN, Johnson T, Vinayak A, Kuvaldina A, Skinner OT, Wustefeld-Janssens BG. Comparison of methods to determine primary tumour size in canine apocrine gland anal sac adenocarcinoma. *J Small Anim Pract*. 2020;61**:** 185-189. doi: 10.1111/jsap.13104
104. Schott CR, Tatiersky LJ, Foster RA, Wood GA. Histologic Grade Does Not Predict Outcome in Dogs with Appendicular Osteosarcoma Receiving the Standard of Care. *Vet Pathol*. 2018;55**:** 202-211. doi: 10.1177/0300985817747329
105. Schultheiss PC. Histologic features and clinical outcomes of melanomas of lip, haired skin, and nail bed locations of dogs. *J Vet Diagn Invest*. 2006;18**:** 422-425. doi: 10.1177/104063870601800422
106. Schultheiss PC. A retrospective study of visceral and nonvisceral hemangiosarcoma and hemangiomas in domestic animals. *J Vet Diagn Invest*. 2004;16**:** 522-526. doi: 10.1177/104063870401600606
107. Schwab TM, Popovitch C, DeBiasio J, Goldschmidt M. Clinical outcome for MCTs of canine pinnae treated with surgical excision (2004-2008). *J Am Anim Hosp Assoc*. 2014;50**:** 187-191. doi: 10.5326/JAAHA-MS-6039
108. Selting KA, Powers BE, Thompson LJ, et al. Outcome of dogs with high-grade soft tissue sarcomas treated with and without adjuvant doxorubicin chemotherapy: 39 cases (1996-2004). *J Am Vet Med Assoc*. 2005;227**:** 1442-1448. doi: 10.2460/javma.2005.227.1442
109. Shiu KB, Flory AB, Anderson CL, et al. Predictors of outcome in dogs with subcutaneous or intramuscular hemangiosarcoma. *J Am Vet Med Assoc*. 2011;238**:** 472-479. doi: 10.2460/javma.238.4.472
110. Sierra Matiz OR, Santilli J, Anai LA, et al. Prognostic significance of Ki67 and its correlation with mitotic index in dogs with diffuse large B-cell lymphoma treated with 19-week CHOP-based protocol. *J Vet Diagn Invest*. 2018;30**:** 263-267. doi: 10.1177/1040638717743280
111. Silvestri S, Porcellato I, Mechelli L, Menchetti L, Rapastella S, Brachelente C. Tumor Thickness and Modified Clark Level in Canine Cutaneous Melanocytic Tumors. *Vet Pathol*. 2019;56**:** 180-188. doi: 10.1177/0300985818798094
112. Simoes JP, Schoning P, Butine M. Prognosis of canine mast cell tumors: a comparison of three methods. *Vet Pathol*. 1994;31**:** 637-647. doi: 10.1177/030098589403100602
113. Skor O, Fuchs-Baumgartinger A, Tichy A, Kleiter M, Schwendenwein I. Pretreatment leukocyte ratios and concentrations as predictors of outcome in dogs with cutaneous mast cell tumours. *Vet Comp Oncol*. 2017;15**:** 1333-1345. doi: 10.1111/vco.12274
114. Skorupski KA, Alarcón CN, de Lorimier LP, LaDouceur EEB, Rodriguez CO, Rebhun RB. Outcome and clinical, pathological, and immunohistochemical factors associated with prognosis for dogs with early-stage anal sac adenocarcinoma treated with surgery alone: 34 cases (2002-2013). *J Am Vet Med Assoc*. 2018;253**:** 84-91. doi: 10.2460/javma.253.1.84

115. Spangler WL, Culbertson MR, Kass PH. Primary mesenchymal (nonangiomatous/nonlymphomatous) neoplasms occurring in the canine spleen: anatomic classification, immunohistochemistry, and mitotic activity correlated with patient survival. *Vet Pathol*. 1994;31**:** 37-47. doi: 10.1177/030098589403100105
116. Spangler WL, Kass PH. The histologic and epidemiologic bases for prognostic considerations in canine melanocytic neoplasia. *Vet Pathol*. 2006;43**:** 136-149. doi: 10.1354/vp.43-2-136
117. Spangler WL, Kass PH. Pathologic and prognostic characteristics of splenomegaly in dogs due to fibrohistiocytic nodules: 98 cases. *Vet Pathol*. 1998;35**:** 488-498. doi: 10.1177/030098589803500603
118. Stefanello D, Avallone G, Ferrari R, Roccabianca P, Boracchi P. Canine cutaneous perivascular wall tumors at first presentation: clinical behavior and prognostic factors in 55 cases. *J Vet Intern Med*. 2011;25**:** 1398-1405. doi: 10.1111/j.1939-1676.2011.00822.x
119. Tanis JB, Simlett-Moss AB, Ossowksa M, et al. Canine anal sac gland carcinoma with regional lymph node metastases treated with sacculectomy and lymphadenectomy: Outcome and possible prognostic factors. *Vet Comp Oncol*. 2022;20**:** 276-292. doi: 10.1111/vco.12774
120. Thamm DH, Weishaar KM, Charles JB, Ehrhart EJ, 3rd. Phosphorylated KIT as a predictor of outcome in canine mast cell tumours treated with toceranib phosphate or vinblastine. *Vet Comp Oncol*. 2020;18**:** 169-175. doi: 10.1111/vco.12525
121. Thompson JJ, Morrison JA, Pearl DL, et al. Receptor Tyrosine Kinase Expression Profiles in Canine Cutaneous and Subcutaneous Mast Cell Tumors. *Vet Pathol*. 2016;53**:** 545-558. doi: 10.1177/0300985815610388
122. Thompson JJ, Pearl DL, Yager JA, Best SJ, Coomber BL, Foster RA. Canine subcutaneous mast cell tumor: characterization and prognostic indices. *Vet Pathol*. 2011;48**:** 156-168. doi: 10.1177/0300985810387446
123. Thompson JJ, Yager JA, Best SJ, et al. Canine subcutaneous mast cell tumors: cellular proliferation and KIT expression as prognostic indices. *Vet Pathol*. 2011;48**:** 169-181. doi: 10.1177/0300985810390716
124. Vail DM, Powers BE, Getzy DM, et al. Evaluation of prognostic factors for dogs with synovial sarcoma: 36 cases (1986-1991). *J Am Vet Med Assoc*. 1994;205**:** 1300-1307.
125. Valli VE, Kass PH, San Myint M, Scott F. Canine lymphomas: association of classification type, disease stage, tumor subtype, mitotic rate, and treatment with survival. *Vet Pathol*. 2013;50**:** 738-748. doi: 10.1177/0300985813478210
126. van Lelyveld S, Warland J, Miller R, et al. Comparison between Ki-67 index and mitotic index for predicting outcome in canine mast cell tumours. *J Small Anim Pract*. 2015;56**:** 312-319. doi: 10.1111/jsap.12320
127. Vargas THM, Pulz LH, Ferro DG, et al. Galectin-3 Expression Correlates with Post-surgical Survival in Canine Oral Melanomas. *J Comp Pathol*. 2019;173**:** 49-57. doi: 10.1016/j.jcpa.2019.10.003
128. Vascellari M, Giantin M, Capello K, et al. Expression of Ki67, BCL-2, and COX-2 in canine cutaneous mast cell tumors: association with grading and prognosis. *Vet Pathol*. 2013;50**:** 110-121. doi: 10.1177/0300985812447829
129. Wei BR, Halsey CH, Hoover SB, et al. Agreement in Histological Assessment of Mitotic Activity Between Microscopy and Digital Whole Slide Images Informs Conversion for Clinical Diagnosis. *Acad Pathol*. 2019;6**:** 2374289519859841. doi: 10.1177/2374289519859841
130. Wilcock BP, Peiffer RL, Jr. Morphology and behavior of primary ocular melanomas in 91 dogs. *Vet Pathol*. 1986;23**:** 418-424. doi: 10.1177/030098588602300411

131. Willcox JL, Marks SL, Ueda Y, Skorupski KA. Clinical features and outcome of dermal squamous cell carcinoma in 193 dogs (1987-2017). *Vet Comp Oncol*. 2019;17**:** 130-138. doi: 10.1111/vco.12461
132. Wittenberns BM, Thamm DH, Palmer EP, Regan DP. Canine Non-Angiogenic, Non-Myogenic Splenic Stromal Sarcoma: a Retrospective Clinicopathological Analysis and Investigation of Podoplanin as a Marker of Tumour Histogenesis. *J Comp Pathol*. 2021;188**:** 1-12. doi: 10.1016/j.jcpa.2021.07.006
133. Yale AD, Priestnall SL, Pittaway R, Taylor AJ. Thymic epithelial tumours in 51 dogs: Histopathologic and clinicopathologic findings. *Vet Comp Oncol*. 2022;20**:** 50-58. doi: 10.1111/vco.12705
134. Yamazaki H, Sasai H, Tanaka M, et al. Assessment of biomarkers influencing treatment success on small intestinal lymphoma in dogs. *Vet Comp Oncol*. 2021;19**:** 123-131. doi: 10.1111/vco.12653
135. Zini E, Nolli S, Ferri F, et al. Pheochromocytoma in Dogs Undergoing Adrenalectomy. *Vet Pathol*. 2019;56**:** 358-368. doi: 10.1177/0300985818819174

Risk of Bias

Supplemental Table S8. Risk of bias of the studies on the mitotic index (MI) based on four domains (study population, outcome assessment, MI methods, and data analysis).

Study	Study population	Outcome assessment	MI method	Data analysis	Overall
Misdorp and Hart [1]	○	○	○	⊖	⊖
Phillips et al. [2]	○	○	○	⊖	⊖
Sarli et al.[3]	○	⊕	○	○	○
Simoes et al.[4]	⊕	○	○	⊖	⊖
Yamamoto et al.[5]	⊖	○	○	⊖	⊖

⊕, low risk of bias; ○, moderate risk of bias; ⊖, high risk of bias

Prognostic Value

Supplemental Table S9. Summary of the prognostic association of the mitotic index determined by the evaluated studies.

**Study**	**Tumor type and case number**	**Outcome assessment**	**Association with outcome?**			
			**Survival time**	**Relapse-free interval**	**Metastasis**	**Recurrence**
Misdorp and Hart [1]	Osteosarcoma, skeletal (n = 97)	Postmortem	–	–	No (p = 0.98)	–
Phillips et al. [2]	Lymphoma, multicentric (n = 41)	Not reported	No (p = 0.66)	No (p = 0.56)	–	–
Sarli et al.[3]	Mammary tumors, malignant (n = 60)	Regular clinical examinations	No (p > 0.39)	–	–	–
Simoes et al.[4]	Mast cell tumors of the skin (n = 87)	Survey	–	–	No (p > 0.05)	Yes (p ≤ 0.05)
Yamamoto et al.[5]	Aortic body tumors (n = 13)	Postmortem	–	–	No (p > 0.05)	–

References

1. Misdorp W, Hart AA. Some prognostic and epidemiologic factors in canine osteosarcoma. *J Natl Cancer Inst*. 1979;62**:** 537-545. doi: 10.1093/jnci/62.3.537
2. Phillips BS, Kass PH, Naydan DK, Winthrop MD, Griffey SM, Madewell BR. Apoptotic and proliferation indexes in canine lymphoma. *J Vet Diagn Invest*. 2000;12**:** 111-117. doi: 10.1177/104063870001200202
3. Sarli G, Preziosi R, Benazzi C, Castellani G, Marcato PS. Prognostic value of histologic stage and proliferative activity in canine malignant mammary tumors. *J Vet Diagn Invest*. 2002;14**:** 25-34. doi: 10.1177/104063870201400106
4. Simoes JP, Schoning P, Butine M. Prognosis of canine mast cell tumors: a comparison of three methods. *Vet Pathol*. 1994;31**:** 637-647. doi: 10.1177/030098589403100602
5. Yamamoto S, Fukushima R, Hirakawa A, Abe M, Kobayashi M, Machida N. Histopathological and immunohistochemical evaluation of malignant potential in canine aortic body tumours. *J Comp Pathol*. 2013;149**:** 182-191. doi: 10.1016/j.jcpa.2012.12.007
